# Trend estimation for complex survey designs of water chemistry indicators from Sierra Nevada Lakes

**DOI:** 10.1007/s10661-018-6963-1

**Published:** 2018-09-19

**Authors:** L. A. H. Starcevich, T. McDonald, A. Chung-MacCoubrey, A. Heard, J. Nesmith, T. Philippi

**Affiliations:** 1 Western EcoSystems Technology, Inc., 2725 NW Walnut Blvd., Corvallis, 97330 USA; 2 National Park Service Inventory and Monitoring Program, Klamath Network, 1250 Siskiyou Blvd, Ashland, OR 97520 USA; 3 National Park Service Inventory and Monitoring Program, Sierra Nevada Network, 47050 Generals Highway, Three Rivers, CA 93271 USA; 4 National Park Service Inventory and Monitoring Division, c/o Cabrillo National Monument, 1800 Cabrillo Memorial Dr., San Diego, CA 92106 USA

**Keywords:** Complex survey design, Trend analysis, Weight

## Abstract

Surveys for long-term monitoring programs managing natural resources often incorporate sampling design complexity. However, design weights are often ignored in trend models of data from complex sampling designs. Generalized random tessellation stratified samples of a simulated population of lakes are selected with various levels of survey design complexity, and three trend approaches are compared. We compare an unweighted trend model, linear regression models of the trend in design-based estimates of annual status, and a probability-weighted iterative generalized least squares (PWIGLS) approach with a linearization variance. The bias and confidence interval coverage of the trend estimate and the size and power of the trend test are used to evaluate weighted and unweighted approaches. We find that the unweighted approach often outperforms the other trend approaches by providing high power for trend detection and nominal confidence interval coverage of the true trend regression parameter. We also find that variance composition and revisit design structure affect the performance of the PWIGLS estimator. When a subpopulation exhibiting an extreme trend is sampled disproportionately to its occurrence in the population, the unweighted approach may produce biased estimates of trend with poor confidence interval coverage. We recommend considering variance composition and potential subpopulation trends when selecting sampling designs and trend analysis approaches.

**Electronic supplementary material:**

The online version of this article (10.1007/s10661-018-6963-1) contains supplementary material, which is available to authorized users.

## Introduction

The National Park Service (NPS) has invested substantial effort into the development of the Vital Signs monitoring program for key indicators at each park. A critical aspect of the Vital Signs program is the use of probabilistic sampling designs to identify monitoring locations (Fancy et al. 2009). Randomization provides the basis for inference to a larger population and a means for quantifying the precision of estimates derived from sample measurements (Lohr 2009). Sampling designs often incorporate sampling complexity such as stratification, cluster sampling, and unequal probability sampling for both statistical and practical efficiency. Additional design complexity may be introduced to monitoring designs with the temporal revisit pattern of sites. Revisit designs consist of panels of sites that are always visited during the same sampling occasion (Urquhart and Kincaid 1999). Panels may be visited annually, on a regular alternating basis, or only a single time. Temporal revisit designs may incorporate the same annual effort to provide a larger total sample of unique sites than if the same sites were visited every year. For model-based analysis such as trend analysis, design complexity is often ignored. When subpopulations exhibiting different trends are surveyed disproportionally to their population distribution, inference that ignores the design complexity may result in biased estimates of trend (Asparouhov 2006).

Unbiased trend estimation and powerful trend detection are necessary aspects of a robust long-term monitoring program (Fancy et al. 2009). If a trend test has low power, then natural resources managers may fail to detect a detrimental trend. Alternatively, a trend may be erroneously detected, triggering an unnecessary mitigation effort. Either error may be costly and squanders the field work, laboratory processing, data entry, and analysis effort required to complete a trend analysis. In this research, we compare several approaches for trend analysis and assess the impact of ignoring design complexity in model-based trend analyses under a range of survey design scenarios. Our goal is to provide guidance to attain unbiased trend estimation and powerful trend testing at nominal significance levels when design complexity is used.

Data are unbalanced when every site is not visited on every occasion (Urquhart et al. 1993; Urquhart and Kincaid 1999). The trend model of Piepho and Ogutu (2002) maintains nominal test size when data are unbalanced, but this approach does not account for unequal design weights due to sampling design complexity. Nonparametric trend detection methods such as the Regional Kendall Tau (Helsel and Frans 2006) require that all sampled sites are visited annually and cannot incorporate data collected from a panel design. A reasonable approach might be to model the trend in annual design-based estimates of some parameter of interest, similar to methods used by Brus and de Gruijter (2011). Modeling approaches that incorporate design weights, such as the probability-weighted iterative generalized least square (PWIGLS) approach (Pfeffermann et al. 1998; Skinner and Holmes 2003), have generally incorporated very simple fixed- and random-effect models that do not specifically examine population-level trend. Furthermore, the size of clusters (defined here as the year-level measurements within each sampled site) examined in the literature for probability-weighted approaches often greatly exceed the number of years needed for effective management of natural resources.

The purpose of this project is to examine potential trend analysis techniques for ecological data collected from complex sampling designs and panel designs for monitoring programs. A secondary goal, similar to that set by Pfeffermann et al. (1998), is to identify trend analysis approaches for complex survey designs that can provide unbiased and precise trend estimation and powerful trend testing with standard statistical software, such as the R statistical software (R Core Team 2016) environment. The three methods examined here include a linear mixed model that does not incorporate design weights and represents a naïve analysis under complex sampling (Piepho and Ogutu 2002), simple and weighted linear regression models on design-based estimates of annual status (Brus and de Gruijter 2011), and PWIGLS (Pfeffermann et al. 1998; Asparouhov 2006). We used NPS Vital Signs monitoring data and simulated data sets to assess performance of these three approaches for trend detection with complex survey data.

## Methods

An ideal statistical method for assessing trend would be applicable to data from simple and complex sampling designs, would be appropriate for both balanced and unbalanced by the temporal revisit design, and could be implemented in widely-used statistical software such as R. The methods investigated here are developed assuming that probabilistic sampling has been used to select the sites in the sample and the inclusion probability for each site is known and accurate. Therefore, any design weighting adjustments for nonresponse or frame error are assumed to have been applied prior to the trend analysis. For more information on adjusting design weights for nonsampling error, see Lessler and Kalsbeek (1992) and Starcevich et al. (2016).

Here, we consider the generalized random tessellation stratified (GRTS) sampling design (Stevens and Olsen 2003, 2004) for selecting survey locations, but other spatially balanced designs are available (see Robertson et al. 2013). GRTS sampling is useful to study because it has been implemented by the NPS Vital Signs monitoring program at various parks (Heard et al. 2012; Irvine et al. 2016; McKinney et al. 2012). GRTS works by generating a set of spatially-balanced sample points and is particularly appealing when sample sizes are small relative to the extent of the sampling frame. GRTS designs can incorporate many types of sampling complexity and is flexible enough to sample either discrete (e.g., lakes), linear (e.g., streams), or infinite (e.g., polygons) resources (Stevens and Olsen 2004). A GRTS sample draw results in a list of sites that, when ordered, are spatially balanced over the study area. Importantly, analysis tools are available in the spsurvey package (Kincaid and Olsen 2015) in R Core Team (2016) to obtain annual design-based estimates of status (Kincaid and Olsen 2015).

We assume that spatially balanced sampling does not contribute to design complexity any more than standard random sampling. The mechanism behind spatial balance is a probabilistic sample, so we treat GRTS samples no differently than standard random samples. Tools exist that incorporate sampling weights from GRTS surveys and take advantage of the spatial balance for more precise design-based estimation (Kincaid and Olsen 2015), but we assume that model-based analyses require no additional tools to account for the spatial balance of the sample.

Temporal revisit designs provide a process for allocating sampling effort over time to increase the overall number of sites visited and to maintain site-level replication at regular time intervals. For example, using the notation of McDonald (2003), a [1-0,1-3] revisit design is a serially alternating augmented design consisting of a single panel visited annually and four panels visited 1 year and then rested for the subsequent 3 years before entering the sample again. Each year, the annual panel and one of the four alternating panels are visited. If sites are visited on an alternating basis, the inclusion probability of a site over time (referred to as a “panel inclusion probability” here) may also be incorporated to account for the unsurveyed panels in a given year.

For monitoring programs that employ GRTS sampling and a panel design, the standard sampling procedure is to select a sample of sites then impose the revisit design on the selected sites. Defining panels as contiguous sets of sites in the GRTS order generates panels that are themselves spatially balanced subsamples of the population. For example, implementing a [1-0,1-3] revisit design with 20 annual sites could be implemented by selecting a GRTS sample of 50 sites and allocating the first 10 sites from the GRTS list in the annual [1-0] panel. Sites 11–20 from the GRTS list would be assigned to the first of the four [1-3] alternating panels, sites 21–30 from the list would be assigned to the second alternating panel, sites 31–40 would be assigned to the third alternating panel, and sites 41–50 would be assigned to the fourth alternating panel.

### Trend analysis methods

In this paper, we focus on GRTS samples with various degrees of design complexity allocated to various temporal revisit schemes. We are primarily interested in detecting trend from the resulting samples and propose three trend analyses that are applicable to a range of complex surveys. Each trend analysis approach is applied to a set of simulation scenarios for a range of sampling designs, revisit designs, sample sizes, monitoring periods, trend magnitudes, and variance structure. We assess the performance of each trend approach by evaluating relative bias and confidence interval coverage of the trend estimate and the size (i.e., the observed type I error rate) and statistical power of the trend test. We begin by describing each of the three trend analysis approaches and the corresponding tests for trend, then we outline the simulation approach, and finally we report the results and provide interpretation for how the simulation results might inform natural resource managers at the survey design and analysis phases.

#### Approach 1: unweighted linear mixed model (PO)

The linear mixed model described by Piepho and Ogutu (2002) is a flexible trend model that does not incorporate design weights. For annual samples of *s* sites and *n* years in the monitoring period, the general linear mixed model proposed by Piepho and Ogutu (2002) for unreplicated data (i.e., one outcome of interest per site and year) is as follows:

$$ {y}_{ij}={\gamma}_0+{x}_j{\gamma}_1+{b}_j+{a}_i+{x}_j{t}_i+{e}_{ij} $$

where:

*i*: 1, …, *s*;
*j*: 1, …, *n*;
*y*_*ij*_: outcome of interest for the *i*th site in the *j*th year;
*x*_*j*_: location-shifted year variable representing the *j*th year, not necessarily consecutive (e.g., 0, 1, 3, 4, etc.);
*γ*_0_ and *γ*_1_: fixed intercept and slope, respectively, of linear trend in time;
*b*_*j*_: random effect of the *j*th year, independent and identically distributed as *N*(0, ${\sigma}_b^2$);
*a*_i_ and *t*_*i*_: random intercept and slope, respectively, of the *i*th site, jointly distributed as bivariate normal with means of 0, respective variances of ${\sigma}_a^2$ and ${\sigma}_t^2$ , and covariance σ_*at*_; and
*e*_*ij*_: unexplained error, independent and identically distributed as *N*(0, ${\sigma}_e^2$).

This model, referred to here as the “PO” approach, represents a standard mixed-effects linear model containing random effects for year- and site-level intercepts and a random site-level slope to account for variation among site-level trends. Note that *y*_*ij*_ may represent a logarithmically-transformed outcome of interest. This transformation is appropriate when exponential trend is modeled or when heterogeneity is present in the residuals from the trend model of an untransformed outcome. Site-specific trend slopes are obtained by adding the population-level fixed slope effect to the site-level random slope effect, by *γ*_1*i*_ = *γ*_1_ + *t*_*i*_. Parameter estimation for the PO model is conducted with restricted maximum likelihood estimation (McCulloch et al. 2008). The PO approach tests for trend in balanced and unbalanced data by comparing the Wald statistic for the mean trend coefficient (i.e., *γ*_1_/se(*γ*_1_)) against a *t*-distribution having degrees of freedom computed by the method of Satterthwaite (1946). The Giesbrecht and Burns (1985) approximation for Sattterthwaite degrees of freedom maintained nominal test size (i.e., of size α) for trend tests, but other approximations such as that of Kenward and Roger (1997) have performed well in simulations (Hu 2002) but may have inflated test size for small samples (Gomez et al. 2005). Because the model does not incorporate survey weights, this approach constitutes a naïve analysis when complex sampling designs have been implemented and provides a basis for assessing bias when design weights are ignored.

When stratified GRTS sampling has been implemented, trends on individual strata (or subpopulations) can be estimated using a separate-slope model that includes fixed effects for the year, stratum, and their interaction. The stratum-level separate-slopes trend model is:

$$ {y}_{hij}={\gamma}_0+{x}_j{\gamma}_1+{\delta}_{h0}+{x}_j{\delta}_{h1}+{b}_j+{a}_i+{x}_j{t}_i+{e}_{ij}, $$

where *h* = 1, …, *H* indexes stratum, *δ*_*h*0_ is the additional (fixed) effect of stratum *h*, and *δ*_*h*1_ is the additional (fixed) effect on slope of the response through time in stratum *h* relative to stratum 1. Note that, in some cases, the residual error variance may vary by stratum levels. This variance heterogeneity should be modeled if present so that model assumptions are met (Pinheiro and Bates 2000). The naïve analysis approach to trend estimation under a stratified design ignores stratum-specific design weights. The population-level trend estimates under this approach take into account variable stratum sizes by weighting stratum-specific parameters by their stratum size. To see this, we note that the trend estimate in stratum 1 (the reference stratum) is *γ*_1_, the estimated trend in stratum *h* > 1 is *γ*_1_ + *δ*_*h*1_, and the non-design weighted estimator of population-level trend is:

$$ {\gamma}_1^{\ast }=\frac{N_1}{N}{\gamma}_1+\frac{N_2}{N}\left(\ {\gamma}_1+{\delta}_{21}\right)+\dots +\frac{N_H}{N}\left(\ {\gamma}_1+{\delta}_{H1}\right)={\gamma}_1+\left({\sum}_{h=2}^H{N}_h{\delta}_{h1}/N\right) $$

where *N*_*h*_ is the population size of the *h*th stratum and $N=\sum \limits_{h=1}^H{N}_h$. This population-level estimator of trend is a simple linear combination of parameters from the stratified trend model, and a standard error for the population-level trend estimate can be obtained by standard methods (i.e., pre- and post-multiply the estimated variance-covariance matrix by the vector of weights). We tested for presence of population-level trend by comparing the Wald *t* test (${\gamma}_1^{\ast }/\mathrm{se}\left({\gamma}_1^{\ast}\right)\Big)$ to a *t*-distribution having degrees of freedom equal to the sum of the Satterthwaite degrees of freedom from the stratum-level trend tests.

#### Approach 2: regression modeling of design-based estimates (SLRDB and WLRDB)

The second approach estimates population-level trend by applying linear regression to annual design-based status estimates. Annual status may be the mean, total, proportion, or other indicator of the condition of a resource during a time period. In this approach, design weights to estimate status are applied prior to model-based inference of trend in order to mitigate the effects of design complexity on analysis.

In our simulation, we considered detecting trend in the mean of a resource. We computed a design-based estimate of the mean in year *j* using the Horvitz-Thompson estimator (Horvitz and Thompson 1952; Cordy 1993):

$$ {\widehat{\mu}}_j=\raisebox{1ex}{$\sum \limits_{i=1}^{n_j}\frac{y_{ij}}{\pi_{ij}}$}\!\left/ \!\raisebox{-1ex}{$\sum \limits_{i=1}^{n_j}\frac{1}{\pi_{ij}}$}\right. $$

where *n*_*j*_ is the sample size in year *j*, and *π*_*ij*_ is the inclusion probability evaluated at site *i* during year *j*.

We assume sampling takes place over time and that some type of panel rotation scheme has been implemented. To account for panel rotation, we decompose the inclusion probability into *π*_*ij*_ = *π*_*i*_*π*_*j*∣*i*_ where *π*_*i*_ is the original design weight from the GRTS sample draw and *π*_*j*∣*i*_ is the inclusion probability for site *i* during year *j*. We characterize *π*_*j*∣*i*_ as the panel-level inclusion probability, which represents the probability that a sampled site is visited in a particular year during the monitoring period. For example, a revisit design containing a panel visited annually and four panels visited once every 4 years (i.e., design [(1-0), (1-3)] in the notation of McDonald (2003)) would have panel inclusion probabilities of 1 and 0.25, respectively. We obtained annual estimates of resource status $\left({\widehat{\mu}}_j\right)$using the survey data analysis tools available in the R package *spsurvey* (Kincaid and Olsen 2015).

We calculated the variance of annual status estimate using the GRTS neighborhood variance estimator for spatially-balanced samples (Stevens and Olsen 2003; Stevens and Olsen 2004). The neighborhood variance estimator for a mean is:

$$ {\widehat{V}}_{\mathrm{NBH}}\left({\widehat{\mu}}_j\right)={\left[{\sum}_i\frac{1}{\pi_{ij}^{\ast }}\right]}^2\sum \limits_i\sum \limits_{i^{\prime}\ne i}{v}_{i{i}^{\prime }}{\left[\frac{y_{ij}}{\pi_{ij}^{\ast }}-\sum \limits_{k\in {D}_i}{v}_{ik}\frac{y_{kj}}{\pi_{kj}^{\ast }}\ \right]}^2 $$

where *D*_*i*_ is the local neighborhood surrounding site *i,* and *v*_*ii’*_ are weights chosen to reflect the behavior of the pairwise inclusion function for GRTS, constrained so that Σ_*i*_ *v*_*ii′*_ = Σ_*i*′_ *v*_*ii′*_ = 1*.* In the case of stratified sampling, the stratum-level means and variances are calculated independently then combined for population-level annual estimates.

To estimate trend, we first fitted the simple linear regression model:

$$ {\widehat{\mu}}_j={\varphi}_0+{x}_j{\varphi}_1+{\varepsilon}_{ij} $$

where *φ*_0_ and *φ*_1_ are (fixed) intercept and slope parameters, respectively, *x*_*j*_ is the year (same as in the previous section), and *ε*_*ij*_ is the normally-distributed unexplained error. We tested trend by comparing the Wald *t* test of the estimated slope ${\widehat{\varphi}}_1/\widehat{\mathrm{se}}\left({\widehat{\varphi}}_1\right)$ to a *t*-distribution with *n*–2 degrees of freedom. We refer to this trend approach as “simple linear regression of design-based estimates,” or SLRDB. In addition, we implemented a weighted least squares approach using weights equal to the inverse of the status estimator’s neighborhood variance estimate, i.e., $1/{\widehat{V}}_{\mathrm{NBH}}\left({\widehat{\mu}}_j\right)$. This weighted approach gave more estimation value to more precise estimates of ${\widehat{\mu}}_j$. We refer to this trend approach as “weighted linear regression of design-based estimates” (WLRDB).

Note that assumptions of the simple linear regression on status include independence of outcomes. While temporal replication at sites is required for trend estimation, high serial correlation among annual status estimates will upset the statistical properties of this trend test. We did not correct for serial correlation in ${\widehat{\mu}}_j$ because the example data set we chose did not exhibit high annual correlation among estimates. If high correlation is present, we recommend applying time series models such as AR(1) or other structures for var(*ε*_*ij*_) or pre-whitening (Yue et al. 2002) prior to regression analysis.

#### Approach 3: probability-weighted iterative generalized least squares

The third trend approach we implemented to detect trend under complex survey designs was a PWIGLS approach (Pfeffermann et al. 1998; Skinner and Holmes 2003). We extend the approach of the PWIGLS models proposed by Pfeffermann et al. (1998) to reflect the mixed model of the PO approach and apply sample weights to obtain regression estimators that are unbiased for their associated population-level parameters. This approach views the design which contains sampling over time as consisting of two stages, one for selection of the sites (stage one) and a second for sampling sites through time. By realizing that sites can be viewed as stage-1 sampling units and that temporal revisits can be viewed as stage-2 sampling units, the methods for complex two-stage designs from Pfeffermann et al. (1998) can be applied. The rest of this section describes the two-stage model of Pfeffermann et al. (1998).

The two-stage model of Pfeffermann et al. (1998) is:

$$ {y}_{ij}={x}_{ij}\beta +{z}_{ij}{u}_i+{z}_{\mathrm{o} ij}{v}_{ij} $$

where *x*_*ij*_, *z*_*ij*_, and *z*_*0ij*_ are fixed row vectors containing covariates such as year or stratum indicators, *β* is a fixed vector of regression parameters, and *u*_*i*_ and υ_*ij*_ are mutually independent random vectors containing effects for the site (stage-1) and time (stage-2), respectively. Random vectors *u*_*i*_ and υ_*ij*_ are assumed to follow multivariate normal distributions, i.e., *u*_*i*_ ~ *N*(0, Ω) and *υ*_*ij*_ ~ *N*(0, *σ*^2^).

To illustrate the PWIGLS model, we note that the PO model is a special case. To see this, we define the *u*_*i*_ vector to contain the site-level random intercepts and slopes (i.e., *a*_*i*_ and *t*_*i*_ of the PO approach), the *z*_*ij*_ vector to be (1, *x*_*ij*_) where *x*_*ij*_ is the year value, and *υ*_*ij*_ to contain the random intercept and residual (*b*_*j*_ and *e*_*ij*_ of the PO approach). If *a*_*i*_ and *t*_*i*_ are modeled as correlated, the PWIGLS model is equivalent to the PO model.

We incorporated design weights into the PWIGLS model as follows. We defined the site-based (stage-1) sampling weight to be *w*_*i*_. These site-based weights come from the initial spatial design, and in this paper are GRTS design weights. We define the temporal (stage-2) sampling weight to be *w*_*j|i*_. These temporal sampling weights come from the panel revisit scheme and are equal to the inverse of the panel inclusion probability, i.e., *w*_*j*∣*i*_  = 1/*π*_*j*∣*i*_. The total design weight for site *i* during the *j*th year is *w*_*ij*_ = *w*_*i*_ * w_*j|i*_. We follow the “Step A only” approach advocated by Pfeffermann et al. (1998) with the design-adjusted weight and apply the standard IGLS algorithm. The data transformation is achieved by replacing *z*_*ij*_ with ${w}_i^{-1/2}{z}_{ij}$, *z*_0*ij*_ with ${w}_{ij}^{-1/2}{z}_{0 ij}$.

Pfeffermann et al. (1998) provide additional adjustments for PWIGLS estimators that incorporate a modification called “Step B” for variance components estimation and scaling of second-stage weights to reduce bias and improve consistency. However, the initial modification without scaling (A-only method) is found in simulation to yield precise and unbiased estimates of both fixed effects and variance components when sampling is invariant, so this additional step was not incorporated. Pfeffermann et al. (1998) and Asparouhov (2006) applied their methods to mixed models with simple structures and did not address trend estimation specifically. In this work, we extend the PWIGLS approach to include the more complex trend model of the PO approach and compare the results for a range of sampling design factors. We also examined a range of scaling methods for design weights (Asparouhov 2006), and these methods are described in the Electronic supplementary material (ESM) 1.

The standard error of the regression coefficient for trend was obtained with the linearization variance estimator (Pfeffermann et al. 1998; Skinner et al. 1989), which incorporates design weights to calculate a variance-covariance matrix for the estimated fixed effects based on Taylor series linearization. Trend was assessed with a Wald *t* test and Satterthwaite degrees of freedom. Confidence intervals were constructed from the standard error obtained from the linearization variance estimator and were based on a *t*-distribution with Satterthwaite degrees of freedom. Initial simulations indicated that degrees of freedom from the PWIGLS methods were inflated, so the degrees of freedom from the PO approach were used for trend testing and confidence interval construction.

As with the PO model, a separate-slopes model was used when a subpopulation trend was modeled. The relative proportions of the strata were used to obtain an estimate of the population-level trend. Stratum structure was incorporated into the linearization variance estimator to approximate the standard error of the population-level trend. Degrees of freedom for trend testing were calculated as the sum of the degrees of freedom from the two stratum-level trend coefficients (analogous to the calculation of ${\gamma}_1^{\ast }$ in the discussion of “Approach 1: unweighted linear model (PO)”). R code used to calculate estimates of trend for all trend analysis approaches is provided in ESM 2.

### Pilot data

**Fig. 1:**
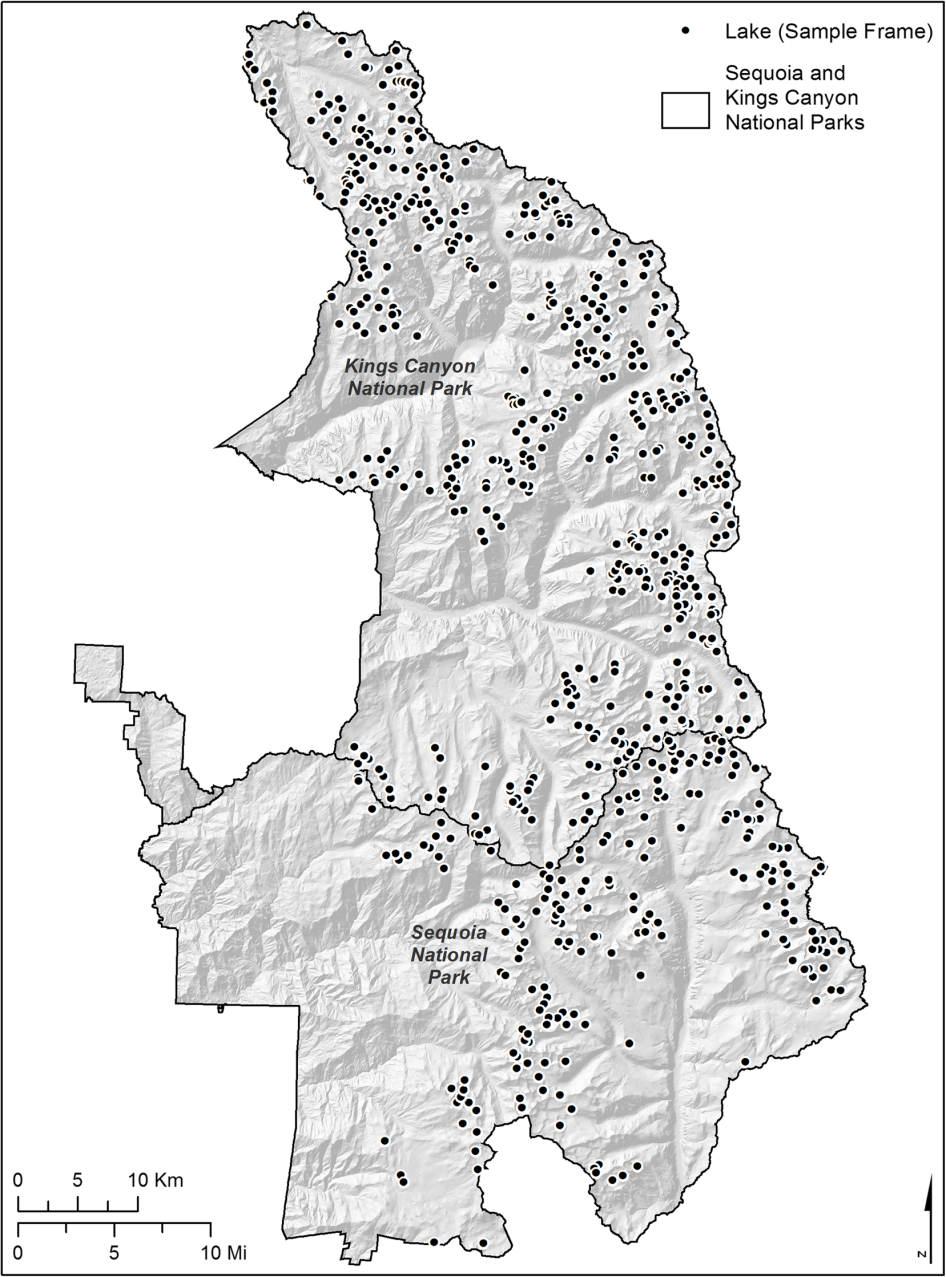
Sampling frame of 684 lakes in Sequoia and Kings Canyon National Parks

We obtained pilot data for use in our simulations from annual surveys of mountain lakes in the Sierra Nevada range (Heard et al. 2012). Annual surveys of a population of 684 mountain lakes in Sequoia and Kings Canyon National Parks (SEKI, Fig. 1) measured chemistry indicators to determine if lake chemistry changed over time. For survey efficiency and safety, the lake chemistry survey utilized unequal sampling based on estimated survey costs for each lake and down-weighted the probability of sampling remote and difficult-to-access lakes. The SEKI lake chemistry surveys were conducted from 2008 to 2013, and the panel revisit scheme was [1-0,1-3]. We focused on acid neutralizing capacity (ANC) because this indicator was less susceptible to detection limit issues and demonstrated a variance composition similar to several other lake chemistry indicators. Nonsampling error, such as nonresponse error and detection limit error, is an important analytical issue that cannot be ignored (Lessler and Kalsbeek 1992). However, in this research, we assume complete data and focus on the impact of complex sampling design weights.

A natural-log transformation was applied to the ANC data to better meet model assumptions of the PO model. The natural-log transformation was required to meet the assumption of variance homogeneity for all indicators examined from this water chemistry data set. This transformation also provides a useful interpretation of trend as a percent annual change. Therefore, we proceed assuming that a natural-log transformation is appropriate for the outcome of interest and interpretation will assume this transformation is applied. The coefficients from the PO model were used to simulate populations exhibiting similar mean and variance characteristics but with known trend. Our investigation of partial autocorrelation plots indicated little serial correlation in residuals from the PO model, so no serial correlation was included in our simulated populations.

The sources of variation for ecological indicators could affect the statistical properties of our trend detection procedures (Urquhart and Kincaid 1999). One estimator may do well when year-to-year variation is high, while another may perform poorly. To investigate the impact of variance composition on the trend approaches, we constructed a modified ANC-like variable which had the same mean but different variance composition than the original ANC. We constructed the modified ANC variable (Table 1) to contain the same random-slope variance, residual variance, and total variance as the original, but lower year-to-year variation. We reduced year-to-year variation in the original ANC (0.0157) to just 0.0001 in the modified version. We added the reduction in year-to-year variation (i.e., 0.0156) to the site-to-site variance of the modified variable in order to maintain the same level of overall variance. The original ANC indicator represents “high” year-to-year variation and the modified ANC indicator represents “low” year-to-year variation.

**Table 1:**
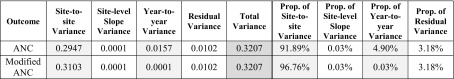
Simulation variance components

Light shading indicates the variance components that differ between the two outcomes and the dark shading emphasizes that total variance is equal for the two outcomes

### Simulation

We implemented Monte Carlo simulations based on the PO model fitted to pilot data and compared the ability of our three approaches to detect trend. For each combination of sample design, sample size, temporal revisit design, monitoring period, and trend magnitude (Table 2), we simulated ANC and modified ANC from the PO model and applied all approaches.

**Table 2:** Simulation inputs and levels

Simulation input	Levels
GRTS sampling designs	Equiprobable, stratified, and unequal probability sampling
Annual sample sizes	20, 35, and 50 sites
Revisit designs	[1-0], [1-0,1-3], and [1-3]
Monitoring periods	12 and 24 years
Annual trends	0%, 1% (24 years), and 2% (12 years)
Subpopulation trends	0 and 4%
Variance structure	ANC, modified ANC

In the simulation exercise, we investigated equiprobable, stratified, and unequal probability sampling designs. Based on previous studies (Berg et al. 2005; Nanus et al. 2009) and observed values of ANC in the SEKI pilot data, we simulated stratified sampling from high- and low-elevation lakes, defining the cutoff between the strata at 3100 m. The high-elevation stratum contained 598 lakes (88% of population) and the low-elevation stratum contained 84 lakes (12% of population). We allocated 60% of total sample size to the high stratum, 40% to low elevation lakes to simulate disproportionate sampling of more accessible low-elevation lakes.

**Fig. 2:**
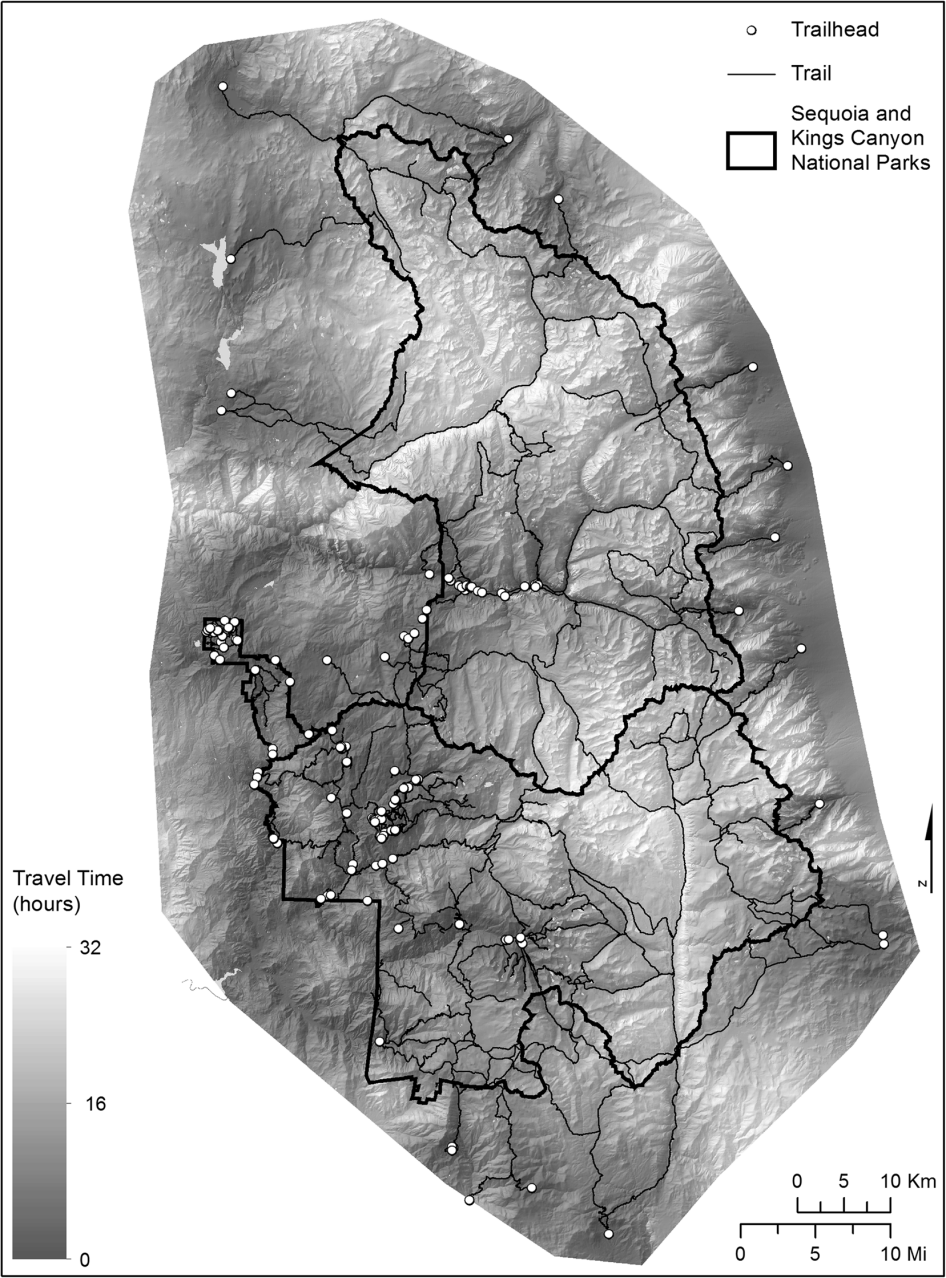
Cost surface model of travel hours to each lake in the sampling frame of 684 lakes in Sequoia-Kings Canyon National Park. From Heard et al. (2012)

We simulated an unequal probability design by implementing the cost surface the travel time estimates of Heard et al. (2012) which were calculated as a function of slope, land cover, elevation, and distance from trailheads (Fig. 2). To draw our unequal probability samples, we divided travel time estimates into six cost classes and allocated sample size in a way that disproportionally sampled more lower-cost classes. Ordered from low- to high-cost classes, the relative proportions of the cost classes in the population are 6, 13, 29, 25, 18, and 8%. We allocated sampling effort as 20, 34, 34, 5, 5, and 2%. This allocation represents a higher proportion of lakes sampled in the lower cost classes than is represented in the population as a whole. The unequal probability design was similar in nature to the stratified sample design because travel time was positively correlated with the elevation (Pearson correlation coefficient = 0.72, *p* value < 0.0001), but the allocation of sampling effort was designed to be more disproportionate relative to the population.

We examined three revisit designs: [1-0], [1-0,1-3], and [1-3]. These revisit designs represent, respectively, a single panel of sites visited every year, an annual panel of sites combined with four panels visited on an alternating schedule, and a set of four panels visiting on an alternating schedule with no annual panel. We simulated samples of 20, 35, and 50 sites each year. Therefore, these annual sample sizes represent the size of each panel in the [1-0] design and each of the four panels in the [1-3] design. The annual panel [1-0,1-3] always contained 10 sites, so the alternating panels always included annual samples of 10, 25, and 40 sites to correspond to the annual total sample sizes of 20, 35, and 50 sites, respectively. Under stratified sampling, the annual panel always contained six sites in the high-elevation stratum and four sites in the low-elevation stratum.

We simulated populations by imposing known trend on pilot data estimates in each successive year, then adding estimated variance components. We imposed annual trends of 2% over 12 years and 1% over 24 years on pilot data, each resulting in net increases in mean ANC of 27%. Trends of 0% were simulated to assess trend test size. For some simulation scenarios, we generated higher trends of 4% at high-elevation lakes to assess the impact of an extreme trend at an undersampled subpopulation. We drew GRTS samples from each simulated population and allocated sites sequentially down the list to panels according to the revisit design.

We applied all trend analysis approaches to 500 simulated populations in each design scenario and summarized results across these iterations. We assessed the relative bias of the trend coefficient, the 90% confidence interval coverage (proportion of confidence intervals containing that contained the true trend coefficient), trend test size (proportion of iterations for which a trend was detected when no trend was present), and trend test power (proportion of iterations for which a trend was accurately detected). We computed relative bias of the trend coefficient as the proportional difference of the trend coefficient compared with the true trend coefficient, or $\left(\widehat{\gamma_1}-{\gamma}_1\right)/{\gamma}_1$. We considered a trend estimate to be biased if the true trend coefficient was overestimated or underestimated by 5% or more. Confidence interval coverage was calculated as the proportion of iterations for which the 90% confidence interval of the estimator contained the true trend regression parameter value. We tested trend at a significance level of 0.10 but allowed test size up to 0.13 to represent a roughly nominal trend test to account for simulation variation.

## Results

Complete simulation results, including the results for the six PWIGLS scaling methods, are provided in ESM 3. The results for the six scaling methods were very similar for the simulation scenarios examined in this research, so the results of the “A-only” method of Pfeffermann et al. (1998) are reported as the PWIGLS results for clarity.

### Relative bias of trend coefficient

**Fig. 3:**
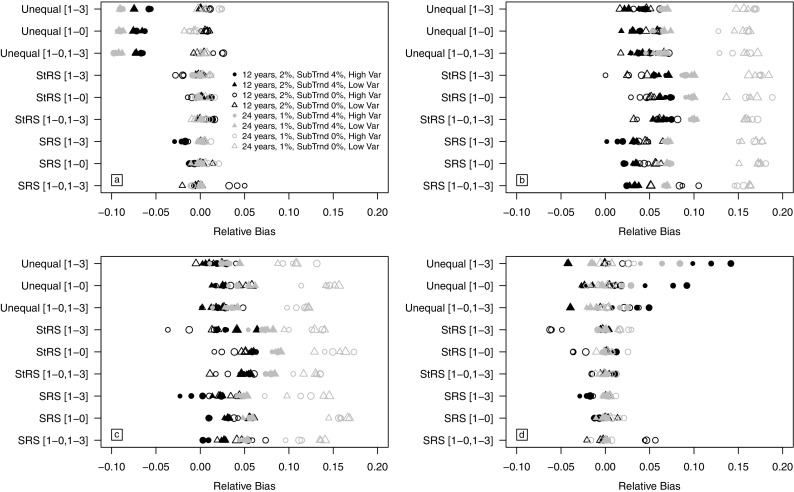
Mean relative bias of the trend regression coefficient for all simulation scenarios for **a** the PO approach, **b** the SLRDB approach, **c** the WLRDB approach, and **d** the PWIGLS approach. Scenarios include 12- or 24-year monitoring periods with 2 or 1% annual trends, respectively; annual subpopulation trends ("SubTrnd")  of 0 or 4%; and low (“low var”) or high (“high var”) year-to-year variation. Plotting symbol size increases with sample size

For most simulation scenarios, the PO and PWIGLS trend approaches resulted in unbiased estimates of the ANC trend regression coefficient (Fig. 3a, d). For the ANC outcome, relative bias is closest to zero for the PO and PWIGLS methods when simple random sampling was used. The SLRDB and WLRDB approaches tended to overestimate trend, but the relative bias of the trend coefficient was smaller for the shorter monitoring period. Estimates of the ANC trend regression coefficient were generally unbiased for both stratified and unequal probability GRTS sampling, for both monitoring periods, and for the PO and PWIGLS methods when a subpopulation trend was not present. However, trend was underestimated by about 6% when the PWIGLS method was used with the [1-3] monitoring design and stratified sampling for a 12-year monitoring period exhibiting a 2% annual trend. When a 24-year trend was estimated, the SLRDB trend estimate overestimated the true trend coefficient by 12.11 to 18.84% for an overall population trend of 1% and by 4.55 to 10.27% when an additional subpopulation trend of 4% was simulated. Bias was reduced for the WLRDB approach, but only when the subpopulation trend of 4% was simulated (i.e., for larger trend values) for 83% of the simulation scenarios. Estimates of the ANC trend regression coefficient were generally unbiased for both stratified and unequal probability GRTS sampling, both monitoring periods, and for the PO and PWIGLS methods when a subpopulation trend was not present.

Relative bias in the trend coefficient was similar for the modified ANC variable, except estimates from the PWIGLS approach were unbiased for all simulation scenarios (Fig. 3d). PO estimates were unbiased for all scenarios except for when a subpopulation trend was present in an undersampled subpopulation, and the SLRDB and WLRDB approaches were positively biased by as much as 18% for 24-year monitoring periods. In contrast, the PWIGLS estimates were unbiased for all simulation scenarios for the modified ANC variable with low year-to-year variation. The WLRDB trend estimate was less biased than the SLRDB trend estimates for all but one (99.5%) of the simulations.

When unequal probability GRTS sampling was used and a subpopulation trend was present, the WLRDB approach produced the least biased estimates of the 12-year ANC trend (Fig. 3c). The PO method underestimated trend by 5.15 to 7.91% (Fig. 3a) and the PWIGLS methods overestimated by 4.49 to 14.15% for method A (Fig. 3d). When a subpopulation trend of 4% was induced over 24 years for the ANC variable (Fig. 3a), the PO method underestimated the true trend regression coefficient by 8.56 to 9.75% for unequal probability sampling. In contrast, the WLRDB approach was unbiased for all revisit designs except a slight bias of 5.26% for an annual sample size of 50 sites with a [1-0] revisit design and equiprobable GRTS sampling. The PWIGLS approach was unbiased for the [1-0] and [1-0,1-3] revisit designs. The PO estimate of the modified ANC trend coefficient for unequal probability sampling underestimated the true value by 6.55 to 7.97% for 12 years and by 8.54 to 9.58% for 24 years when a subpopulation trend of 4% was modeled (Fig. 3a).

### Trend confidence interval coverage

**Fig. 4:**
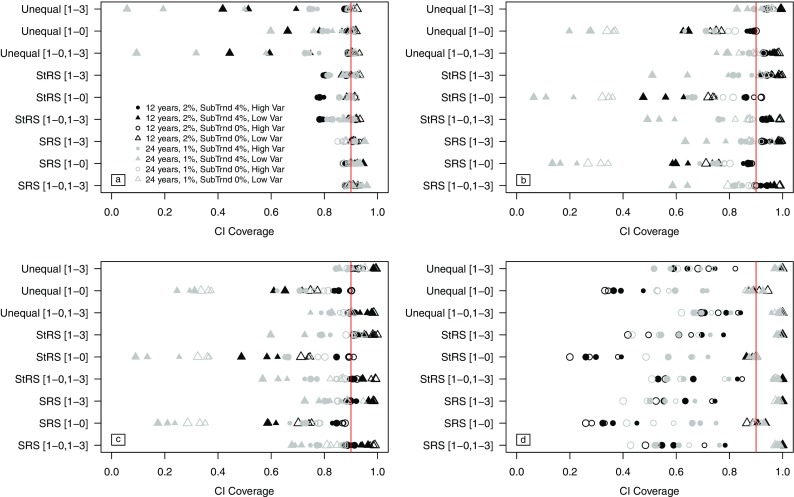
Mean confidence interval coverage of the trend regression coefficient for all simulation scenarios for **a** the PO approach, **b** the SLRDB approach, **c** the WLRDB approach, and **d** the PWIGLS approach. Nominal (90%) confidence is indicated by the vertical line at 0.90. We assume a type I error rate of 0.10. Scenarios include 12- or 24-year monitoring periods with 2 or 1% annual trends, respectively; annual subpopulation trends ("SubTrnd") of 0 or 4%; and low (“low var”) or high (“high var”) year-to-year variation. Plotting symbol size increases with sample size

Overall, confidence interval coverage for the ANC trend regression coefficient was closest to 90% for the PO trend approach (Fig. 4a). The SLRDB and WLRDB approaches performed well for the 12-year monitoring period, with the WLRDB approach providing slightly more nominal coverage. For the 24-year time period, the SLRDB and WLRDB approaches performed better for the [1-0,1-3] and [1-3] revisit designs. The PWIGLS approach provided poor coverage for the ANC trend regression coefficient ranging from 36 to 81%.

For the modified ANC outcome, the PO model demonstrated nominal confidence interval coverage for nearly all scenarios incorporating a 12-year monitoring period (Fig. 4a). The SLRDB and WLRDB approaches demonstrated nominal confidence interval coverage of the modified ANC trend coefficient in most cases, but coverage was poorest for the [1-0] revisit design and for the longer monitoring period. Modified ANC confidence interval coverage for the PWIGLS estimates of trend provided the best coverage of the true modified ANC trend, ranging from 86 to 94% across all simulations scenarios in which the [1-0] revisit design was used.

When a subpopulation trend in ANC was present, confidence interval coverage under the PO method was consistently below 90% for stratified sampling across both monitoring periods and for unequal probability sampling in the 24-year monitoring period (Fig. 4a). When a subpopulation trend was present for a 12-year monitoring program, the WLRDB approach provided nominal confidence interval coverage for both stratified and unequal probability GRTS sampling when the [1-0,13] and [1-3] designs were used. When a subpopulation trend was present for a 24-year monitoring program, confidence interval coverage for the [1-0,1-3] and [1-3] revisit designs was closest to nominal for the SLRDB approach (Fig. 4b) when unequal probability sampling was used, but no approach provided nominal confidence interval coverage for stratified GRTS sampling.

For the modified ANC variable, confidence interval coverage from the PO model was exceptionally low when unequal probability sampling was used and a subpopulation trend was present (Fig. 4a). However, coverage was near nominal for the PWIGLS approach. As with the ANC data, confidence intervals from the SLRDB and WLRDB approaches covered the true modified ANC trend coefficient less frequently for the 24-year monitoring period than for the 12-year trend period.

### Test size

Test size is evaluated as the proportion of iterations for which the null hypothesis of no significant trend is erroneously rejected. Therefore, we assess trend test size only for simulations in which no population- or subpopulation-level trends are present. Trend test size is compared with an alpha value of 0.10, the predetermined type I error rate.

**Fig. 5:**
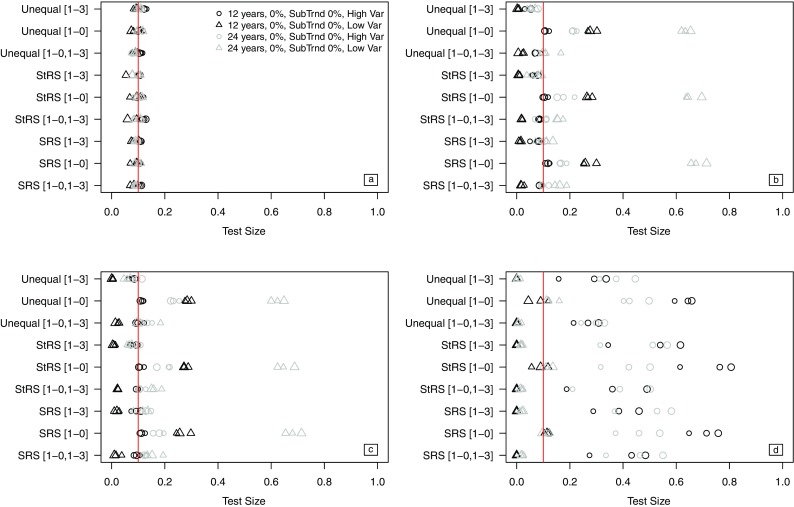
Mean test size of the trend test (type I error = 0.10) for all simulation scenarios for **a** the PO approach, **b** the SLRDB approach, **c** the WLRDB approach, and **d** the PWIGLS approach. Nominal (0.10) test size is indicated by the vertical line at 0.10. Scenarios include 12- or 24-year monitoring periods with 0% annual trend and low (“low var”) or high (“high var”) year-to-year variation. Plotting symbol size increases with sample

For the ANC variable, the size of the trend test based on the *t*-distribution from the PO model ranged between 0.07 and 0.14 for all simulation scenarios (Fig. 5a). The SLRDB and WLRDB approaches yielded generally nominal trend tests for all revisits designs over 12 years and for 24-year monitoring periods when the [1-0,1-3] and [1-3] revisit designs were used. Tests of ANC trend from PWIGLS models were nearly always inflated, with test size values as high as 0.81.

For the modified ANC variable, the size of trend tests from the PO model ranged from 0.05 to 0.11 for monitoring periods of 12 years and from 0.07 to 0.12 for monitoring periods of 24 years (Fig. 5a). The trend tests from the SLRDB and WLRDB approaches achieved nominal levels when a [1-0,1-3] or [1-3] revisit design was used. However, test size was slightly inflated for the [1-0,1-3] revisit design over a 24-year period. Nearly every scenario for the modified ANC variable using the PWIGLS approaches resulted in trend testing at significance level 0.10, but test size was often close to 0 when the revisit designs [1-0,1-3] or [1-3] were used.

### Test power

Test power was assessed only for scenarios exhibiting nominal test size since inflated test size leads to overly-optimistic power approximation. This restricted the power analysis to the PO approach for all scenarios, the SLRDB and WLRDB approaches for all scenarios except a 24-year monitoring cycle and a [1-0] revisit design, and the PWIGLS approaches when the year-to-year variation was low (the modified ANC variable). In general, the highest power for PO trend tests with nominal test size was obtained with the [1-0,1-3] revisit design over a 12-year monitoring period and with a [1-3] revisit design over a 24-year monitoring period. The [1-0] revisit design provided the highest power for trend tests based on the SLRDB and WLRDB approach, but in many cases nominal test size was only attained when the revisit design consisted of a larger set of unique sites. The power to detect trends in the modified ANC indicator with the PWIGLS approach was generally highest with the [1-0] revisit design; test size was inflated for the ANC indicator so power was not assessed.

The power of the *t* test for a 12-year annual trend of 2% was consistently below 0.8 for trend tests from the PO and linear regression models with nominal test size. However, test power exceeded 0.90 when a subpopulation trend was present for the 12-year monitoring period. For a 1% annual trend over a 24-year period, trend test power was slightly higher for the SLRDB approach (range: 0.77 to 0.85) than for tests based on the PO model (range, 0.75 to 0.82) when simple random sampling was used and a [1-0,1-3] revisit design was used. However, the power of 24-year trend tests based on the PO model (range, 0.70 to 0.98) was considerably higher than that observed for the SLRDB approach (range, 0.59 to 0.87) or the WLRDB approach (range, 0.61 to 0.79) when stratified sampling or unequal probability sampling were used. Power to detect a 24-year trend with a large subpopulation trend exceeded 0.996 for all simulation scenarios.

Power to detect trends in the modified ANC variable was uniformly high for scenarios in which nominal test size was attained, with power of at least 0.98 for the test from the PO model and at least 0.78 for the trend tests from the SLRDB and WLRDB approaches for a 24-year monitoring period and annual samples of at least 35 sites. Power from the PWIGLS approach with nominal test size exceeded 0.83 for 12-year trend tests when at least 35 sites were surveyed per year and exceeded 0.91 for all 24-year data sets.

### Variance components estimation

Because variance components are not estimated in the SLRDB and WLRDB approaches, the bias of variance components was only calculated for the PO and PWIGLS methods. The ANC variance components estimates from the PO model were generally unbiased. Estimates of site-to-site variance component $\left({\sigma}_a^2\right)$ from the PO model were unbiased for all simulation scenarios and both outcomes of interest. Estimates of the ANC random site-level slope $\left({\sigma}_t^2\right)$ from the PO model were unbiased when no subpopulation trend was present except when stratified random sampling was used over a 12-year monitoring period. Estimates of year-to-year variation $\left({\sigma}_b^2\right)$ in the ANC outcome from the PO model were unbiased for equiprobable and unequal probability sampling, but year-to-year variance estimates for stratified sampling underestimated the true variance by − 53.33 to − 47.55%. Estimates of residual variation in the ANC outcome were unbiased for the PO method except when stratified sampling was used (range, 61.45 to 80.26%). In contrast, variance component estimated with the PWIGLS approaches were severely overestimated for nearly all scenarios and both outcomes of interest. The exception occurred for estimates of residual variance estimation which were unbiased for the modified ANC variable for all approaches.

## Discussion

Simulations were used to compare measures of trend detection and estimation for a range of complex sampling design elements and variance compositions with three trend analysis methods. The performance of the modeling approaches was assessed by examining the bias and confidence interval coverage of the trend estimate, trend test size and power, and bias of variance components estimates. Several general patterns emerged from these simulations that provide guidance for sampling design, trend detection, and trend analysis.

Overall, trend detection with two-sided hypothesis tests of no trend was preferable with a *t* test based on the PO model for the examined scenarios because tests maintained nominal test size and demonstrated high power for a two-sided trend test. Even for the cases when PO confidence interval coverage was low due to undersampled subpopulation trends, the trend *t* test from the PO approach maintained nominal test size and high power to detect a nonzero trend. For the simulation inputs examined in this work, the Wald *t* test obtained from the PO model should provide a powerful two-sided test of trend. We observed that the highest power to detect trends with the PO approach were generally obtained with the [1-0,1-3] revisit design over a 12-year monitoring period and with the [1-3] revisit design over a 24-year monitoring period. This result demonstrates the reduced benefit of the annual panel that arises from increasing site-level replication over time (Urquhart et al. 1993). Starcevich et al. (2018) also demonstrated the improvement in power from using revisit designs with more unique sites over a 12-year monitoring period.

In most scenarios, the estimate of the trend regression coefficient from the PO approach was unbiased. However, design complexity resulting in disproportionate sampling relative to the population (e.g., due to easy access) when a subpopulation trend was present led to poor confidence interval coverage and underestimation of the trend regression coefficient by as much as 10% when a larger subpopulation trend was present and design complexity was ignored. This magnitude of bias may not be ecologically significant for some indicators; in these cases, the conservative approach would be to ignore the design weights and proceed with the PO approach. However, this bias might influence management decisions for some indicators, so the impact of the weighted vs unweighted approaches should be considered when determining which approach to use. Combining a subpopulation trend with unequal probability sampling was used to create a “worst-case” scenario of undersampling a subpopulation that exhibit a substantially different trend. Note that undersampling of a subpopulation could also occur when small sample sizes are drawn with stratified random sampling or equiprobable sampling; these results do not necessarily imply a problem inherent with unequal probability sampling. The simulation exercise demonstrates that bias occurs when the sampling design weights are ignored for the undersampled subpopulation exhibiting a substantially different trend.

For an outcome with relatively small year-to-year variance (0.03% of the total variation in this investigation), the PWIGLS method provided more accurate trend estimates with better confidence interval coverage than the unweighted PO method when the [1-0] revisit design was used. Confidence interval coverage was near 1 for the [1-0,1-3] and [1-3] designs, indicating that the linearization variance overestimates the standard error of the trend regression coefficient for those revisit designs. This variance inflation may be due to the reduced site-level temporal replication when panel designs are used. For an outcome with large year-to-year variance component (5% of the total variation in this investigation), unequal probability sampling relative to a category defining the subpopulation, implementation of a [1-3] revisit design, and estimation with the WLRDB approach were found to provide both unbiased estimates of trend and near-nominal confidence interval coverage. Note that Brus and de Gruijter (2011) also found the serially alternating revisit design to be most accurate among the five revisit designs they examined for a 10-year monitoring period.

Variance composition and fixed effects modeling can provide some insight into the best sampling design and trend analysis approach. Without the benefit of a simulation, a trend analyst must consider a priori that subpopulation trends may be present in a population and ensure that sample sizes are adequate and spatially dense enough to represent those subpopulations. Then a trend analysis incorporating a separate-slope PO model may be used to determine if statistically significant subpopulation trends are present and to estimate variance components. Note that a large random slope variance may indicate the presence of subpopulations exhibiting different trends. Assumptions of equal variation must be assessed and accounted for in the variance structure if necessary (Pinheiro and Bates 2000).

The PO model was found to provide nominal confidence interval coverage for unequal probability sampling and a [1-0] revisit design for a 12-year sampling period but not for a 24-year sampling period when year-to-year variation was high and a subpopulation was present. In this case, a hybrid approach might be incorporated. If a subpopulation trend is detected at 12 years, three new panels can be added to form a [1-3] revisit design for the next 12 years. The WLRDB approach, which appears to perform best when a larger number of total sites are visited, may then be applied to the 24-year time series. Note that a large initial GRTS sample (or sample with large oversample) should be drawn so that the sample of sites is easily expanded while maintaining spatial balance and tractable design weights. While this amended revisit design was not tested in this research, this adaptation is likely to result in higher power than if the [1-0] revisit design was used due to the increase in the number of sites in the sample. Ultimately, examination of modified designs in a simulation study would provide the best basis for determining the best course of action midway through a monitoring program.

The variance components estimates from the PWIGLS methods greatly overestimated the true parameter values for all variance components except the residual variation in the equiprobable case. This result is likely because the step-B approach of Pfeffermann et al. (1998) was not used. Variance component estimation also inflated the degrees of freedom from the PWIGLS modeling results requiring trend testing based on degrees of freedom from the unweighted PO model. Estimates of variance components for power analysis and degrees of freedom for trend testing should be obtained from the unweighted model (PO) as also found by Asparouhov (2006).

When a subpopulation trend was simulated and the PO model applied, bias in the random-slope variance component was minimized when stratified sampling was used, but not when equiprobable or unequal probability sampling was used. This result is likely due to the inclusion of a stratum fixed effect in the trend model of the mean for stratified sampling. Explicit modeling of the subpopulation trends with the separate-mean model reduced unexplained variation among site-level trends for more accurate estimation. Identifying these subpopulations and including their classification in the mean structure of the trend model will improve random-slope variance component estimation for equiprobable and unequal probability sampling designs also.

When equiprobable sampling and an alternating panel design are used, the PWIGLS method differs from the PO method only by panel weighting and the implementation of the linearization variance. Comparisons of relative bias and confidence interval coverage indicate no benefit of panel weighting when panel structure is used with equiprobable sampling. In the equiprobable case, the revisit structure merely represents data imbalance which is handled well by the PO method when Satterthwaite degrees of freedom are used (Piepho and Ogutu 2002). Therefore, weighting an equiprobable sample to account for panel weights appears unnecessary.

**Fig. 6:**
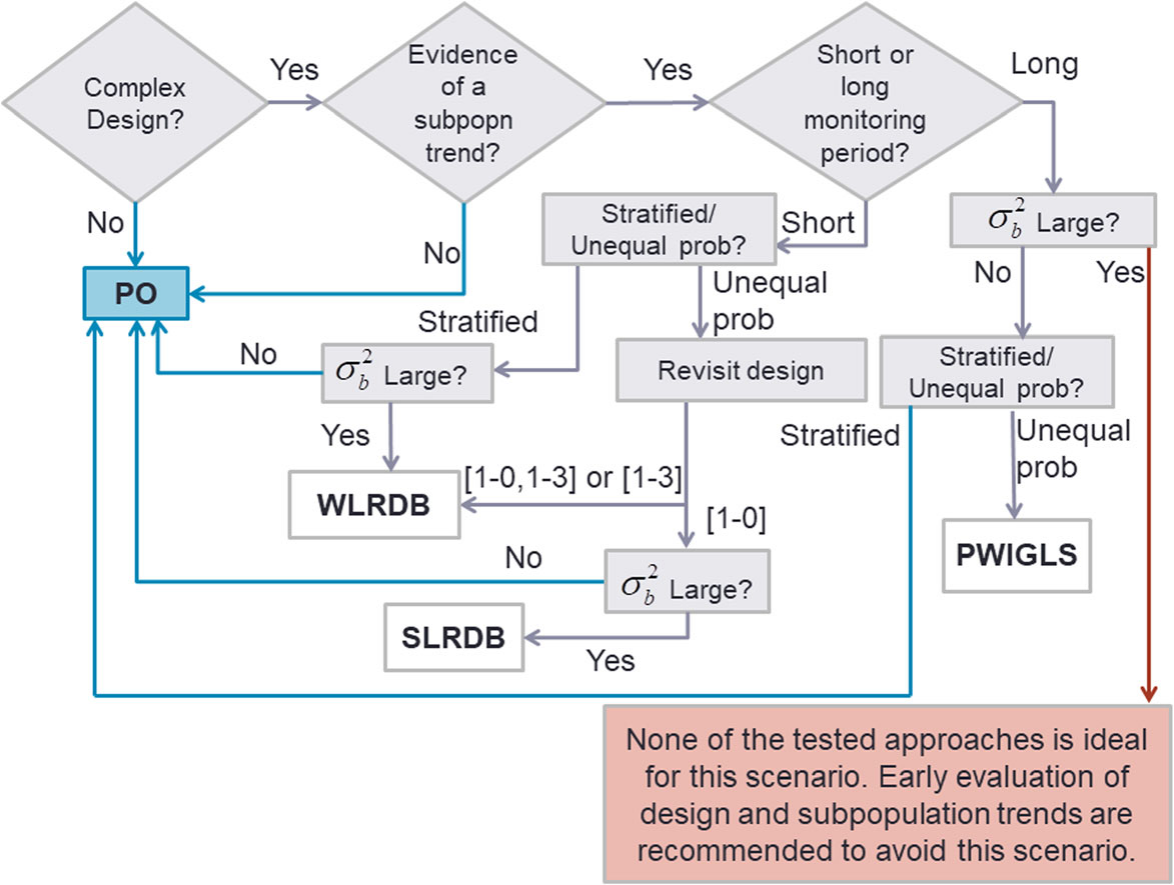
Flowchart of recommended approaches to trend analysis for data collected from complex sample designs. Analytical approaches include PO, WLRDB, SLRDB, and PWIGLS

The results of the simulation are summarized in the following flowchart (Fig. 6). Our results demonstrated that the unweighted PO approach is appropriate for many scenarios involving complex designs. Within the scenarios we examined, a key piece of information for determining the appropriate trend analysis method is whether there is evidence of a subpopulation trend. Thus, subpopulations must have been adequately sampled to allow identification of subpopulation trends, or other ecological knowledge may be used to infer whether subpopulation trends exist. If subpopulation trends exist and complex sampling design was used (i.e., not an equiprobable design), then the length of the monitoring period, the sampling design, the revisit design, and the variance composition must be considered when determining the appropriate trend analysis approach. To better understand the impact of weighting on trend inference, we recommend comparing results of weighted and unweighted approaches.

Note that the flowchart summarizes the broad patterns demonstrated in the simulation results but may omit more detailed patterns in the interest of general recommendations. Also note that the recommendations may differ for other continuous outcomes exhibiting different mean structure or variance structure than was examined in this simulation study. Our study was limited to data that benefitted from a logarithmic transformation. We did not address issues related to nonsampling error such as minimum detection limit data or nonresponse mechanisms that cannot be remedied with only the data from accessible sites. Therefore, the results of this simulation exercise may provide general recommendations but additional study for a wider range of indicators would be useful for broader inference.

We recommend that natural resource managers prioritize elements of design complexity and then use the results of this research to select the remaining design elements. Elements of survey design must often be optimized to provide inference for multiple and competing goals. The more restrictive aspects of the design (e.g., a revisit design that reduces trampling impacts at a site) should be determined first so that the remaining design elements can be chosen to optimize inference. For example, if stratified or unequal probability sampling is planned for a 12-year monitoring period and a subpopulation trend is suspected, then incorporating a [1-0] revisit design allows the PO or SLRDB trend approaches to be used. If a [1-3] revisit design is desired for a long-term monitoring program, then indicators with low year-to-year variation should be chosen. Note that identifying indicators with low year-to-year variation is always recommended for long-term monitoring when powerful trend detection is desired (Urquhart and Kincaid 1999).

## Conclusions

For the range of trend magnitudes, sample sizes, and monitoring periods explored in this research, the PO model, which does not incorporate design weights, performed very well, even for some scenarios with sampling and revisit design complexity. When sampling design complexity is required, avoiding additional complexity from revisit designs provides the most nominal confidence interval coverage for the PWIGLS approach. Alternately, collecting data from a [1-3] design and applying the WLRDB approach for trend estimation may provide robust trend inference.

The PWIGLS approach implemented here was very sensitive to variance composition. Nominal confidence interval coverage is obtained for the trend in the modified ANC variable only when the [1-0] design was used but rarely for the ANC variable with large year-to-year variation. Even relatively small year-to-year variation greatly impacts the power to detect trend (Urquhart and Kincaid 1999). The PO approach performed well when the year-to-year variation was high, with the exception of scenarios involving undersampled subpopulations with an extreme trend. The PWIGLS approach may be improved by incorporating step-B approach (Pfeffermann et al. 1998) to improve variance components estimates. This adaptation may also improve PWIGLS trend coefficient standard errors from the linearization variance that employ the inflated variance components estimates.

We demonstrated that unmodeled design complexity can lead to biased trend inference and poor trend tests under some approaches. In most cases explored here, however, the unweighted approach provided powerful trend tests of nominal test size and unbiased trend estimates with nominal confidence interval coverage. Accurate identification of subpopulations undergoing dramatic changes is necessary for unbiased trend inference at the population level. Attention to these details at the survey design stage will allow natural resource managers the flexibility to respond with the appropriate sampling design, temporal revisit design, and trend analysis approach.

## Electronic supplementary material

ESM 1 (PDF 621 kb)

ESM 2 (PDF 216 kb)

ESM 3 (PDF 3585 kb)
