# Supplementary material for: Trend estimation for complex survey designs of water chemistry indicators from Sierra Nevada Lakes
Source: Environ Monit Assess. 2018 Sep 19;190(10):596. doi: 10.1007/s10661-018-6963-1 (PMC6153522; doi:10.1007/s10661-018-6963-1)
Supplement: Supplementary file 1 — (PDF 621 kb) [file 10661_2018_6963_MOESM1_ESM.pdf]

Supplement A: PWIGLS scaling methods

The PWIGLS weighting approach advocated by Pfeffermann et al. (1998) directly applies the sampling design weight to the random effects design weight and employs the standard IGLS algorithm. Referred to as the "A-only" method, the random effects design matrix is adjusted by replacing z_{ij} with $w_i^{-1/2} z_{ij}$, z_{0ij} with $w_{ij}^{-1/2} z_{0ij}$. In addition to the A-only method, scaling methods and alternative weighting approaches were examined for the PWIGLS approach (Pfeffermann et al. 1998, Asparouhov 2006). Scaling standardizes the design weights to reduce small-sample bias introduced by inflated weights. In standard scaling procedures, the scaling factor is applied to the design weight across both stages of sampling, impacting both w_i *and* $w_{j|i}$. In some situations, the stage-one and stage-two effects are independent. A sampling mechanism is considered "invariant" when the random first-stage cluster effects are independent of the second-stage individual design weights given the covariate information at each level (Asparouhov 2006). In this application, invariance is achieved when the probability that a site is sampled in a given year is independent of the site's random intercept and slope. In other words, the temporal revisit design is applied equally among the sample of sites. In this case, scaling is only used at the year-level and not at the site-level.

Scaling factors for site- and year-level weights in the PWIGLS approach are defined in Table 1. Define the mean year-level design weight as $\bar{w}_i = \frac{1}{n_i}\sum_j w_{ij}$, where n_i is the number of years in which site i was surveyed. The effective sample size is defined as in Potthoff et al. (1992) and describes the effective number of years surveyed at site i based on design weights. Let s_{0i} be the effective sample size of years for site i such that $s_{0i} = \sum_j w_{ij}^2 / \left(\sum_j w_{ij}\right)^2$. Let the "effective mean site-level design weight" be defined as $\tilde{w}_i = \frac{1}{s_{0i}}\sum_j w_{ij}$.

The combinations of weighting and scaling methods were then used to obtain 6 different PWIGLS estimates. The A-only method for PWIGLS (Table 1) does not employ scaling for site-level or year-level design weights (Pfeffermann et al. 1998). The A method scales panel weights with the mean site-level design weight, $\bar{w}_i$, and the invariant version, AI, adjusts the scaling at the site level. Similarly, the B and BI methods scale with the effective mean site weight, $\tilde{w}_i$. Method C scales only at the year level with the inverse of the average year-level weight and is commonly used for mean estimation. Note that, when the second-stage selection probabilities for years within a site are the same for all years within a panel and this holds for all panels, methods A and B are equivalent and methods AI and BI methods are equivalent under the current setting. This result holds because the

panel inclusion probabilities always sum to the number of sites within a panel which is equal to the effective sample size under the same conditions. This situation would occur when samples are fully enumerated each year and sample sizes within each panel do not change, regardless of the monitoring period length. However, this scenario is unlikely given accessibility issues, changing budgets, and other factors that affect annual sampling effort.

Table 1: Scaling factors for each PWIGLS method

PWIGLS Method	Site Scaling Factor	Year Scaling Factor	Description
A-only	1	1	No scaling for either site-level or year-level design weights.
A	1	$\bar{w}_i$	Scaling for both site-level and year-level design weights with the mean site-level design weight.
AI	$1/\bar{w}_i$	$\bar{w}_i$	Scaling for only the year-level design weights with the mean site-level design weight.
B	1	$\tilde{w}_i$	Scaling for both site-level and year-level design weights with the effective mean site-level design weight.
BI	$1/\tilde{w}_i$	$\tilde{w}_i$	Scaling for only the year-level design weights with the effective mean site-level design weight.
C	1	$\sum_{ij} w_{ij} \Big/ \sum_{i} n_i$	Scaling for both site-level and year-level design weights with the grand mean of design weights across sites.

Asparouhov (2006) found that the AI method was best for minimizing the bias in estimates of the mean (i.e. fixed effects such as the trend coefficient), regardless of the cluster size and informative index. Method BI resulted in the most accurate residual error variance, and the unweighted analysis (represented by the Piepho and Ogutu (2002) model) is most appropriate for estimating the random effects variance components (Asparouhov 2006). The simulation results for the six PWIGLS scaling methods are provided in Supplement C.
