# Supplementary material for: Trend estimation for complex survey designs of water chemistry indicators from Sierra Nevada Lakes
Source: Environ Monit Assess. 2018 Sep 19;190(10):596. doi: 10.1007/s10661-018-6963-1 (PMC6153522; doi:10.1007/s10661-018-6963-1)
Supplement: Supplementary file 2 — (PDF 216 kb) [file 10661_2018_6963_MOESM2_ESM.pdf]

Supplement B: R code for analysis

```
TrendEsts <- function(dat, design)  {


# Function to calculate trend for the PO approach, SLRDB & WLRDB approach,
# and 6 methods of the PWIGLS approach.
# Dependent packages: lme4

# LA Starcevich 8.5.16

Sites = sort(unique(dat$Site))
Years=sort(unique(dat$Year))
yrs=sort(unique(dat$WYear))
ma = length(Sites)
mb = length(yrs)
models=c("Aonly","A","AI","B","BI","C")
modelnum = length(models)

ans = data.frame(matrix(NA,9,10))
ans[,1] = c("PO","SLRDB","WLRDB","Aonly","A","AI","B","BI","C")
names(ans) =
c("Approach","intercept","trend","SEtrend","sig2a","sig2t","sigat","sig2b","sig2e","tr
end_df")
#####################################################################################
# METHOD 1: Naive analysis -- P & O
#####################################################################################


if(design!="StRS")  fit_PO<-lmer(LogY ~ WYear + (1|Year) +(1+WYear|Site), data=dat,
           control = lmerControl(check.nobs.vs.nRE = "warning"))
if(design=="StRS")  fit_PO<-lmer(LogY ~ WYear + Elev_Stratum + (1|Year)
+(1+WYear|Site), data=dat, control = lmerControl(check.nobs.vs.nRE = "warning"))
       ans[1,2] <-fixef(fit_PO)[1]                    # mu
       ans[1,3] <- fixef(fit_PO)[2]                   # slope
       ans[1,4] <- sqrt(vcov(fit_PO)[2,2])            # SE of trend estimate
       ans[1,5] <- VarCorr(fit_PO)$Site[1,1]          # var(ai)
       ans[1,6] <- VarCorr(fit_PO)$Site[2,2]          # var(ti)
       ans[1,7] <- VarCorr(fit_PO)$Site[1,2]          # cov(ai,ti)
       ans[1,8] <- VarCorr(fit_PO)$Year[1]            # var(bj)
       ans[1,9] <- attr(VarCorr(fit_PO),"sc")^2       # residual var
       ans[1,10] <-summary(fit_PO)$coef[2,3]          # using lmerTest

print(summary(fit_PO))
##############################################################################
# Model 2: SLRDB/WLRDB
##############################################################################

# Calculate annual design-based estimates
MeanEsts<-data.frame(matrix(NA,mb,3))
MeanEsts[,1]<-yrs

for (g in 1:mb) {
dat.g<-dat[dat$Year==Years[g],]
if(design!="StRS")
       MeanEsts[g,2:3] <-cont.analysis(
              sites=data.frame(siteID=dat.g$Site, rep(TRUE,nrow(dat.g))),
              subpop= data.frame(siteID=dat.g$Site, Popn1=rep(1, nrow(dat.g))),
              design= data.frame(siteID=dat.g$Site,
              wgt= dat.g$wgt, xcoord = dat.g$xcoord, ycoord = dat.g$ycoord),
              data.cont= data.frame(siteID=dat.g$Site, Y=dat.g$Y), conf=90)$Pct[8,6:7]

if(design=="StRS")
       MeanEsts[g,2:3] <-cont.analysis(
              sites=data.frame(siteID=dat.g$Site, rep(TRUE,nrow(dat.g))),
```

```
            subpop= data.frame(siteID=dat.g$Site, Popn1=rep(1, nrow(dat.g))),
            design= data.frame(siteID=dat.g$Site,
            wgt= dat.g$wgt, xcoord = dat.g$xcoord, ycoord = dat.g$ycoord,
            stratum=dat.g$Elev_Stratum),
            data.cont= data.frame(siteID=dat.g$Site, Y=dat.g$Y), conf=90)$Pct[8,6:7]
       }
       MeanEsts[,3]<-as.numeric(MeanEsts[,3])
       names(MeanEsts)<-c("Year","Est.Mean","SE")
       MeanEsts$WYear<-MeanEsts$Year-min(MeanEsts$Year)

# Simple linear regression of design-based estimates (SLRDB)
       fit_SLRDB<-lm(log(Est.Mean) ~ WYear, data=MeanEsts)
       ans[2,2] <- coef(fit_SLRDB)[1]                              # mu
       ans[2,3] <- coef(fit_SLRDB)[2]                 # slope
       ans[2,4] <-sqrt(vcov(fit_SLRDB)[2,2])   # SE
       ans[2,5] <- ans[2,6] <- ans[2,7] <- ans[2,8] <- 0
       ans[2,9] <- (summary(fit_SLRDB)$sigma)^2
       ans[2,10] <-summary(fit_SLRDB)$df[2]

# Weighted linear regression of design-based estimates (WLRDB)
       fit_WLRDB<-lm(log(Est.Mean) ~ WYear, weights=1/(SE^2), data=MeanEsts)
       ans[3,2]<- coef(fit_WLRDB)[1]                  # mu
       ans[3,3]<- coef(fit_WLRDB)[2]                  # slope
       ans[3,4]<-sqrt(vcov(fit_WLRDB)[2,2])           # SE
       ans[3,5] <- ans[3,6] <- ans[3,7] <- ans[3,8] <- 0
       ans[3,9] <- (summary(fit_WLRDB)$sigma)^2
       ans[3,10] <-summary(fit_WLRDB)$df[2]

########################################################################################
# PWIGLS methods
########################################################################################

# Calculate Stage 2 weights
AnnualReps<-table(dat$Site)
# inverse of proportion of years sampled
Stage2wt<-data.frame(Site=names(AnnualReps),Stage2wt= mb/AnnualReps)[,-2]
names(Stage2wt)<-c("Site", "Stage2wt")
Stage2wt<-Stage2wt[!is.na(Stage2wt$Stage2wt),]
dat<-merge(dat, Stage2wt)

fit.PWIGLS<-vector(modelnum, mode="list")

for (m in 1:modelnum) {
       fit.PWIGLS.m<-PWIGLS(Z=getME(fit_PO,"Z"), dat=dat, stage1wt="wgt",
       stage2wt="Stage2wt",method=models[m],BS=BS,ML="ML", design=design,
       fitPO=fit_PO)
       ans[3+m,2]<- as.numeric(fit.PWIGLS.m[1])              # mu
       ans[3+m,3]<- as.numeric(fit.PWIGLS.m[2])              # slope
       ans[3+m,4]<-as.numeric(fit.PWIGLS.m[3])               # SE
       ans[3+m,5]<- fit.PWIGLS.m[5]                          # var(ai)
       ans[3+m,6]<- fit.PWIGLS.m[6]                          # var(ti)
       ans[3+m,7]<- fit.PWIGLS.m[7]                          # cov(ai,ti)
       ans[3+m,8]<- fit.PWIGLS.m[8]                          # var(bj)
       ans[3+m,9]<- fit.PWIGLS.m[9]                          # var(eij)
       ans[3+m,10]<- ans[1,10]                               # Using PO df
}


return(ans)
}
```

```
#######################################################################################
#######################################################################################
# Function to calculate PWIGLS
#######################################################################################
#######################################################################################
PWIGLS<-function(Z, dat, stage1wt, stage2wt, method, BS, ML, design, fitPO) {
# wrapper function for PWIGLS_ALL
# Obtains appropriate model, returns quantities for simulation
# mu, beta, SE(beta), sig2a, sig2b, sig2t, sigat, sig2e
# las 4.1.15

if(design!="StRS") {
	fit.all<-PWIGLS_ALL(Z=Z, dat=dat, stage1wt=stage1wt, stage2wt=stage2wt,
	method=method,BS=BS,ML=ML,design=design)
	fit<-fit.all[[1]]
	mu<- fixef(fit)[1]			# intercept
	beta<- fixef(fit)[2]				# slope
	df<-NA # Using PO df in main fcn
	sig2a<- VarCorr(fit)$Site[1,1]		# var(ai)
	sig2t<- VarCorr(fit)$Site[2,2]		# var(ti)
	sigat<- VarCorr(fit)$Site[1,2]		# cov(ai,ti)
	sig2b<- VarCorr(fit)$Year[1]		# var(bj)
	sig2e<- attr(VarCorr(fit),"sc")^2	# var(eij)
	pvalue_chi<-fit.all[[2]]
# Linearization variance -- Pfeffermann 1988
	varij = sig2a + sig2b + dat$WYear*sigat +(dat$WYear^2)*sig2t +sig2e
	SEbeta<-sqrt(LinearizationVar(Site=dat$Site,
		wij=dat[,stage1wt]*dat[,stage2wt],
		xij=dat$WYear,
		eij=dat$LogY-mu-(beta*dat$WYear),
		varYij=varij))
	}

if(design=="StRS") {
	fit.all<-PWIGLS_ALL(Z=Z, dat=dat, stage1wt=stage1wt, stage2wt=stage2wt,
	method=method,BS=BS,ML=ML,design=design)
	fit<-fit.all[[1]]
	mu<- fixef(fit)[1]		# intercept
	betaHigh<- fixef(fit)[2]	# slope of High stratum
	betaLow<- fixef(fit)[4]		# slope of Low stratum-slope of High stratum
	beta=betaHigh +  0.1231672*betaLow
	df<-NA # Using PO df in main fcn
	sig2a<- VarCorr(fit)$Site[1,1]		# var(ai)
	sig2t<- VarCorr(fit)$Site[2,2]		# var(ti)
	sigat<- VarCorr(fit)$Site[1,2]		# cov(ai,ti)
	sig2b<- VarCorr(fit)$Year[1]		# var(bj)
	sig2e<- attr(VarCorr(fit),"sc")^2	# var(eij)
	pvalue_chi<-fit.all[[2]]
# Linearization variance -- Pfeffermann 1988
	varij = sig2a + sig2b + dat$WYear*sigat +(dat$WYear^2)*sig2t +sig2e
	ILij = as.numeric(dat$Elev_Stratum=="Low")
	SEbeta<-sqrt(LinearizationVar_StRS(Site=dat$Site,
		wij=dat$wgt*dat$Stage2wt,
		xij=data.frame(dat$WYear,ILij),
		eij=dat$LogY-mu-(betaHigh*dat$WYear)-(fixef(fit)[3]*ILij)-
		(betaLow*dat$WYear*ILij),varYij=varij))
	}

return(data.frame(mu=mu,beta=beta,SEbeta=SEbeta,df=df,sig2a=sig2a,sig2t=sig2t,sigat=si
gat,sig2b=sig2b,sig2e=sig2e,pvalue_chi=pvalue_chi))
}
```

```
##################################################################################
##################################################################################
# Function to calculate PWIGLS by method
##################################################################################
##################################################################################

PWIGLS_ALL<-function(Z, dat, stage1wt, stage2wt, Sitename="Site", method,
BS,ML,design) {

print(c("Method = ",method))
# Use PWIGLS from Pfeffermann et al 1998 and Asparouhov 2006
# Note that PSU = Level 2 = Stage 1 ==> s2j is weighting adj factor
# Note that SSU = Level 1 = Stage 2 ==> s1j is weighting adj factor

dat$Site<-dat[,Sitename]
SitesTables<-table(dat$Site)
Sites<-unique(as.character(dat$Site))
Years<-sort(unique(dat$Year))
ma=length(Sites)
mb=length(Years)
AnnualSites<-names(SitesTables)[SitesTables==length(Years)]

Z_PWIGLS<-Z

sitecol<-length(unique(dat$Site))*2     # assume 2 random site effects
sitecolindex<-sitecol/2
d<-ncol(Z_PWIGLS)

# Adjust RE design matrix with sqrt of site design weights
for (j in 1:sitecolindex) {
       Site.j<-Sites[j]
       rows.j<-which(Z_PWIGLS[,colnames(Z_PWIGLS) %in% Site.j][,1]==1)

if(method=="PO") {
# Unweighted, no scaling
       s2j<-s1j<-1
       dat[rows.j,stage1wt]<-1
       dat[rows.j,stage2wt]<-1
       }

# Pfeffermann Step A only from Pfeffermann et al. 1998, no scaling
if(method=="Aonly") s2j<-s1j<-1

if(method=="AI") {
       n.j<-length(rows.j)
       wj.bar<-sum(dat[rows.j,stage2wt])/n.j
       s2j<-wj.bar
       s1j<-1/wj.bar
       }

if(method=="BI") {
       n0.j<-(sum(dat[rows.j,stage2wt])^2)/sum(dat[rows.j,stage2wt]^2)
       wj.tilde<-sum(dat[rows.j,stage2wt])/n0.j
       s2j<-wj.tilde
       s1j<-1/wj.tilde
       }

# Asperouhov method A = Pfeffermann Step A, scaling 2 = method in Kovacevic
if(method=="A") {
if(method=="A") {
       n.j<-length(rows.j)
       wj.bar<-sum(dat[rows.j,stage2wt])/n.j
       s2j<-1
```

```
       s1j<-1/wj.bar
       }

# Asperouhov method B = Pfeffermann Step A, scaling 1
if(method=="B") {
       n0.j<-(sum(dat[rows.j,stage2wt])^2)/sum(dat[rows.j,stage2wt]^2)
       wj.tilde<-sum(dat[rows.j,stage2wt])/n0.j
       s2j<-1
       s1j<-1/wj.tilde
       }

# Asperouhov method C
if(method=="C") {
       s2j<-1
       n.j.sum<-sum(SitesTables)  # check this
       wij.sum<-sum(dat[,stage2wt])
       s1j<-n.j.sum/wij.sum
       }

# PSU weight adj
# multiply sqrt design weight by all cols
Z_PWIGLS[rows.j,]<- Z_PWIGLS[rows.j,]/sqrt(dat[rows.j,stage1wt]*s2j)# SSU weight adj

# multiply panel wgt for year effects
if(ma>mb) Z_PWIGLS[rows.j,(sitecol+1):d]<-
Z_PWIGLS[rows.j,(sitecol+1):d]/(sqrt(dat[rows.j,stage2wt]*s1j))
if(ma<=mb) Z_PWIGLS[rows.j,1:mb]<-
Z_PWIGLS[rows.j,1:mb]/(sqrt(dat[rows.j,stage2wt]*s1j))

}  # end site loop

if(ma>mb) {
A<-Z_PWIGLS[,seq(1,sitecol-1,2)]
T<-Z_PWIGLS[,seq(2,sitecol,2)]
B<-Z_PWIGLS[,(sitecol+1):d]
}

if(ma<=mb) {
A<-Z_PWIGLS[,mb+seq(1,sitecol-1,2)]
T<-Z_PWIGLS[,mb+seq(2,sitecol,2)]
B<-Z_PWIGLS[,1:mb]
}

# Sum across rows to pick up each weight
dat$SiteWt<-rowSums(A)
dat$SlopeWt<-rowSums(T)
dat$YearWt<-rowSums(B)

if(design!="StRS") {
if(ML=="REML") fit.PWIGLS.0<-lmer(LogY ~ WYear +(-1+YearWt|Year) +(-
1+SiteWt+SlopeWt|Site), data=dat,
           control = lmerControl(check.nobs.vs.nRE = "warning"))
if(ML=="ML") fit.PWIGLS.0<-lmer(LogY ~ WYear +(-1+YearWt|Year) +(-
1+SiteWt+SlopeWt|Site), data=dat, REML=FALSE,
           control = lmerControl(check.nobs.vs.nRE = "warning"))

# Test trend with Chi square test: Pfeffermann + LaVange FE hypothesis test from
Skinner, Holt, & Smith p.247
       C<-matrix(c(0,1),1,2)
       slope<- matrix(fixef(fit.PWIGLS.0)[2],2,1)
       varslope<-vcov(fit.PWIGLS.0)
       Chi<-as.numeric(t(C%*%slope)%*%solve(C%*%varslope%*%t(C))%*%C%*%slope)
       pvalue_chi<-pchisq(Chi,1, lower.tail = FALSE)
```

```
}
if(design=="StRS") {
if(ML=="REML") fit.PWIGLS.0<-lmer(LogY ~ WYear*Elev_Stratum +(-1+YearWt|Year) +(-
1+SiteWt+SlopeWt|Site), data=dat,
           control = lmerControl(check.nobs.vs.nRE = "warning"))
if(ML=="ML") fit.PWIGLS.0<-lmer(LogY ~ WYear*Elev_Stratum +(-1+YearWt|Year) +(-
1+SiteWt+SlopeWt|Site), data=dat, REML=FALSE,
           control = lmerControl(check.nobs.vs.nRE = "warning"))

# Test trend with Chi square test: Pfeffermann + LaVange FE hypothesis test from
Skinner, Holt, & Smith p.247
       C<-matrix(c(0,1,0,0.1231672),1,4) # weight to get popn trend
       slope<- matrix(c(0,fixef(fit.PWIGLS.0)[2],0,fixef(fit.PWIGLS.0)[4]),4,1)
       varslope<-vcov(fit.PWIGLS.0)
       Chi<-as.numeric(t(C%*%slope)%*%solve(C%*%varslope%*%t(C))%*%C%*%slope)
       pvalue_chi<-pchisq(Chi,1, lower.tail = FALSE)
}

return(list(fit.PWIGLS.0,pvalue_chi))
}


# Take out all list indices
# Format results into a table for comparison?
# Look for a subpopulation trend within elevational strata!
```

```
LinearizationVar <- function (Site,wij,xij,eij,varYij) {
# Calculate the linearization variance (Skinner et al. 1989 p. 82-83)
# of the trend coefficient
# Inputs:
# Site = vector of PSU's
# wij = inclusion weight = PSU wt * SSU wt
# xij = year var for trend estimation
# eij = residual
# varYij = total variance for yij

dTdB0 = matrix(c(sum(wij/varYij),
sum(xij*wij/varYij),
sum(eij*wij*(xij^2)/(varYij^2)),
sum(xij*wij*eij/(varYij^2)),
sum(eij*wij/(varYij^2))),1,5)

dTdB1 = matrix(c(sum(xij*wij/varYij),
sum((xij^2)*wij/varYij),
sum(eij*wij*(xij^3)/(varYij^2)),
sum((xij^2)*wij*eij/(varYij^2)),
sum(xij*eij*wij/(varYij^2))),1,5)

dTdsig2t = matrix(c(sum(eij*wij*(xij^2)/(varYij^2)),
sum(wij*(xij^3)*eij/(varYij^2)),
sum((-(xij^4)*wij/(2*(varYij^2)))+(wij*(xij^4)*(eij^2)/(varYij^3))),
sum((-(xij^3)*wij/(2*(varYij^2)))+(wij*(xij^3)*(eij^2)/(varYij^3))),
sum((-(xij^2)*wij/(2*(varYij^2)))+((xij^2)*wij*(eij^2)/(varYij^3)))),1,5)

dTdsigat = matrix(c(sum(eij*xij*wij/(varYij^2)),
sum(wij*(xij^2)*eij/(varYij^2)),
sum((-(xij^3)*wij/(2*(varYij^2)))+((xij^3)*wij*(eij^2)/(varYij^3))),
sum((-(xij^2)*wij/(2*(varYij^2)))+((xij^2)*wij*(eij^2)/(varYij^3))),
sum((-wij*xij/(2*(varYij^2)))+(wij*xij*(eij^2)/(varYij^3)))),1,5)

dTdsig2abe = matrix(c(sum(eij*wij/(varYij^2)),
sum(xij*wij*eij/(varYij^2)),
sum((-(xij^2)*wij/(2*(varYij^2)))+(wij*(xij^2)*(eij^2)/(varYij^3))),
sum((-wij*xij/(2*(varYij^2)))+(wij*xij*(eij^2)/(varYij^3))),
sum((-wij/(2*(varYij^2)))+(wij*(eij^2)/(varYij^3)))) ,1,5)

I.mat = rbind(dTdB0,dTdB1,dTdsig2t,dTdsigat,dTdsig2abe)
I.mat.inv = solve(I.mat)

g = aggregate(list(g1=eij*wij/varYij,
g2 = xij*eij*wij/varYij,
g3 = (((-wij*(xij^2)/(2*varYij))) + (wij*(xij^2)*(eij^2))/(2*(varYij^2))),
g4 = (((-wij*xij/(2*varYij))) + (wij*xij*(eij^2))/(2*(varYij^2))),
g5 = (((-wij/(2*varYij))) + (wij*(eij^2))/(2*(varYij^2)))),
list(Site=Site), sum)

VarL.T = nrow(g)* var(g[,2:6])
VarL.Beta = I.mat.inv%*%VarL.T%*%I.mat.inv

return(VarL.Beta[2,2])
}
```

```
LinearizationVar_StRS <- function (Site,wij,xij,eij,varYij) {
# Calculate the linearization variance (Skinner et al. 1989 p. 82-83)
# of the trend coefficient
# Inputs:
# Site = vector of PSU's
# wij = inclusion weight = PSU wt * SSU wt
# xij = year var for trend estimation
# eij = residual
# varYij = total variance for yij

ILij<-xij[,2]
xij<-xij[,1]

dTdB0 = matrix(c(sum(wij/varYij),
sum(xij*wij/varYij),
sum(eij*wij*(xij^2)/(varYij^2)),
sum(xij*wij*eij/(varYij^2)),
sum(eij*wij/(varYij^2)),
sum(wij*ILij/varYij),
sum(xij*wij*ILij/varYij)),1,7)

dTdB1 = matrix(c(sum(xij*wij/varYij),
sum((xij^2)*wij/varYij),
sum(eij*wij*(xij^3)/(varYij^2)),
sum((xij^2)*wij*eij/(varYij^2)),
sum(xij*eij*wij/(varYij^2)),
sum(xij*wij*ILij/varYij),
sum((xij^2)*wij*ILij/varYij)),1,7)

dTdsig2t = matrix(c(sum(eij*wij*(xij^2)/(varYij^2)),
sum(wij*(xij^3)*eij/(varYij^2)),
sum((-(xij^4)*wij/(2*(varYij^2)))+(wij*(xij^4)*(eij^2)/(varYij^3))),
sum((-(xij^3)*wij/(2*(varYij^2)))+(wij*(xij^3)*(eij^2)/(varYij^3))),
sum((-(xij^2)*wij/(2*(varYij^2)))+((xij^2)*wij*(eij^2)/(varYij^3))),
sum(eij*wij*(xij^2)*ILij/(varYij^2)),
sum(wij*(xij^3)*eij*ILij/(varYij^2))),1,7)

dTdsigat = matrix(c(sum(eij*xij*wij/(varYij^2)),
sum(wij*(xij^2)*eij/(varYij^2)),
sum((-(xij^3)*wij/(2*(varYij^2)))+((xij^3)*wij*(eij^2)/(varYij^3))),
sum((-(xij^2)*wij/(2*(varYij^2)))+((xij^2)*wij*(eij^2)/(varYij^3))),
sum((-wij*xij/(2*(varYij^2)))+(wij*xij*(eij^2)/(varYij^3))),
sum(eij*xij*wij*ILij/(varYij^2)),
sum(wij*(xij^2)*eij*ILij/(varYij^2))),1,7)

dTdsig2abe = matrix(c(sum(eij*wij/(varYij^2)),
sum(xij*wij*eij/(varYij^2)),
sum((-(xij^2)*wij/(2*(varYij^2)))+(wij*(xij^2)*(eij^2)/(varYij^3))),
sum((-wij*xij/(2*(varYij^2)))+(wij*xij*(eij^2)/(varYij^3))),
sum((-wij/(2*(varYij^2)))+(wij*(eij^2)/(varYij^3))),
sum(eij*wij*ILij/(varYij^2)),
sum(xij*wij*eij*ILij/(varYij^2))) ,1,7)

dTdB2 = matrix(c(sum(wij*ILij/varYij),
sum(xij*wij*ILij/varYij),
sum(eij*wij*(xij^2)*ILij/(varYij^2)),
sum(xij*wij*eij*ILij/(varYij^2)),
sum(eij*wij*ILij/(varYij^2)),
sum(wij*ILij*ILij/varYij),
sum(xij*wij*ILij*ILij/varYij)),1,7)

dTdB3 = matrix(c(sum(xij*wij*ILij/varYij),
sum((xij^2)*wij*ILij/varYij),
```

```
sum(eij*wij*(xij^3)*ILij/(varYij^2)),
sum((xij^2)*wij*eij*ILij/(varYij^2)),
sum(xij*eij*wij*ILij/(varYij^2)),
sum(xij*wij*ILij*ILij/varYij),
sum((xij^2)*wij*ILij*ILij/varYij)),1,7)

I.mat = rbind(dTdB0,dTdB1,dTdsig2t,dTdsigat,dTdsig2abe,dTdB2,dTdB3)
I.mat.inv = solve(I.mat)

g = aggregate(list(g1 = eij*wij/varYij,
g2 = xij*eij*wij/varYij,
g3 = (((-wij*(xij^2)/(2*varYij))) + (wij*(xij^2)*(eij^2))/(2*(varYij^2))),
g4 = (((-wij*xij/(2*varYij))) + (wij*xij*(eij^2))/(2*(varYij^2))),
g5 = (((-wij/(2*varYij))) + (wij*(eij^2))/(2*(varYij^2))),
g6 = eij*wij*ILij/varYij,
g7 = xij*eij*wij*ILij/varYij),
list(Site=Site), sum)

VarL.T = nrow(g)* var(g[,2:8])
VarL.Beta = I.mat.inv%*%VarL.T%*%I.mat.inv

# Use a contrast to get the population level trend variance across two strata
C<-matrix(c(0,1,0,0.1231672),1,4) # weight to get popn trend (note beta4 is a
difference in slopes)
VarC<-C%*%VarL.Beta[c(1:2,6:7),c(1:2,6:7)]%*%t(C)
return(VarC)

}
```

ANC pilot data from Sequoia-Kings Canyon National Park

```
SEKIANC_Example <-
structure(list(Site = structure(c(1L, 1L, 1L, 1L, 1L, 1L, 2L,
2L, 2L, 2L, 2L, 3L, 3L, 3L, 3L, 3L, 3L, 4L, 4L, 4L, 4L, 4L, 4L,
5L, 5L, 5L, 5L, 5L, 5L, 7L, 7L, 8L, 8L, 9L, 9L, 10L, 10L, 11L,
11L, 12L, 12L, 13L, 13L, 15L, 15L, 16L, 16L, 17L, 18L, 19L, 20L,
21L, 22L, 23L, 24L, 25L, 26L, 27L, 28L, 29L, 31L, 32L, 33L, 34L,
35L, 36L, 37L, 38L, 39L, 40L, 41L, 42L, 43L, 44L, 46L, 47L, 48L,
49L, 51L, 51L), .Label = c("SEKI_Lake-001", "SEKI_Lake-002",
"SEKI_Lake-003", "SEKI_Lake-004", "SEKI_Lake-005", "SEKI_Lake-006",
"SEKI_Lake-007", "SEKI_Lake-008", "SEKI_Lake-009", "SEKI_Lake-010",
"SEKI_Lake-011", "SEKI_Lake-012", "SEKI_Lake-013", "SEKI_Lake-014",
"SEKI_Lake-015", "SEKI_Lake-016", "SEKI_Lake-017", "SEKI_Lake-018",
"SEKI_Lake-019", "SEKI_Lake-020", "SEKI_Lake-021", "SEKI_Lake-022",
"SEKI_Lake-023", "SEKI_Lake-024", "SEKI_Lake-025", "SEKI_Lake-026",
"SEKI_Lake-027", "SEKI_Lake-028", "SEKI_Lake-029", "SEKI_Lake-030",
"SEKI_Lake-031", "SEKI_Lake-032", "SEKI_Lake-033", "SEKI_Lake-034",
"SEKI_Lake-035", "SEKI_Lake-036", "SEKI_Lake-037", "SEKI_Lake-038",
"SEKI_Lake-039", "SEKI_Lake-040", "SEKI_Lake-041", "SEKI_Lake-042",
"SEKI_Lake-043", "SEKI_Lake-044", "SEKI_Lake-045", "SEKI_Lake-046",
"SEKI_Lake-047", "SEKI_Lake-048", "SEKI_Lake-049", "SEKI_Lake-050",
"SEKI_Lake-051", "SEKI_Lake-052", "SEKI_Lake-053", "SEKI_Lake-054",
"SEKI_Lake-055", "SEKI_Lake-056", "SEKI_Lake-057", "SEKI_Lake-058",
"SEKI_Lake-059", "SEKI_Lake-060", "SEKI_Lake-061", "SEKI_Lake-062",
"SEKI_Lake-063", "SEKI_Lake-064", "SEKI_Lake-065", "SEKI_Lake-066",
"SEKI_Lake-067", "SEKI_Lake-068", "SEKI_Lake-069", "SEKI_Lake-070",
"SEKI_Lake-071", "SEKI_Lake-072", "SEKI_Lake-073", "SEKI_Lake-074",
"SEKI_Lake-075", "SEKI_Lake-076", "SEKI_Lake-077", "SEKI_Lake-078",
"SEKI_Lake-079", "SEKI_Lake-080", "SEKI_Lake-081", "SEKI_Lake-082",
"SEKI_Lake-083", "SEKI_Lake-084", "SEKI_Lake-085", "SEKI_Lake-086",
"SEKI_Lake-087", "SEKI_Lake-088", "SEKI_Lake-089", "SEKI_Lake-090",
"SEKI_Lake-091", "SEKI_Lake-092", "SEKI_Lake-093", "SEKI_Lake-094",
"SEKI_Lake-095", "SEKI_Lake-096", "SEKI_Lake-097", "SEKI_Lake-098",
"SEKI_Lake-099", "SEKI_Lake-100", "SEKI_Lake-101", "SEKI_Lake-102",
"SEKI_Lake-103", "SEKI_Lake-104", "SEKI_Lake-105", "SEKI_Lake-106",
"SEKI_Lake-107", "SEKI_Lake-108", "SEKI_Lake-109", "SEKI_Lake-110",
"SEKI_Lake-111", "SEKI_Lake-112", "SEKI_Lake-113", "SEKI_Lake-114",
"SEKI_Lake-115", "SEKI_Lake-116", "SEKI_Lake-117", "SEKI_Lake-118",
"SEKI_Lake-119", "SEKI_Lake-120", "SEKI_Lake-121", "SEKI_Lake-122",
"SEKI_Lake-123", "SEKI_Lake-124", "SEKI_Lake-125", "SEKI_Lake-126",
"SEKI_Lake-127", "SEKI_Lake-128", "SEKI_Lake-129", "SEKI_Lake-130",
"SEKI_Lake-131", "SEKI_Lake-132", "SEKI_Lake-133", "SEKI_Lake-134",
"SEKI_Lake-135", "SEKI_Lake-136", "SEKI_Lake-137", "SEKI_Lake-138",
"SEKI_Lake-139", "SEKI_Lake-140", "SEKI_Lake-141", "SEKI_Lake-142",
"SEKI_Lake-143", "SEKI_Lake-144", "SEKI_Lake-145", "SEKI_Lake-146",
"SEKI_Lake-147", "SEKI_Lake-148", "SEKI_Lake-149", "SEKI_Lake-150",
"SEKI_Lake-151", "SEKI_Lake-152", "SEKI_Lake-153", "SEKI_Lake-154",
"SEKI_Lake-155", "SEKI_Lake-156", "SEKI_Lake-157", "SEKI_Lake-158",
"SEKI_Lake-159", "SEKI_Lake-160", "SEKI_Lake-161", "SEKI_Lake-162",
"SEKI_Lake-163", "SEKI_Lake-164", "SEKI_Lake-165", "SEKI_Lake-166",
"SEKI_Lake-167", "SEKI_Lake-168", "SEKI_Lake-169", "SEKI_Lake-170",
"SEKI_Lake-171", "SEKI_Lake-172", "SEKI_Lake-173", "SEKI_Lake-174",
"SEKI_Lake-175", "SEKI_Lake-176", "SEKI_Lake-177", "SEKI_Lake-178",
"SEKI_Lake-179", "SEKI_Lake-180", "SEKI_Lake-181", "SEKI_Lake-182",
"SEKI_Lake-183", "SEKI_Lake-184", "SEKI_Lake-185", "SEKI_Lake-186",
"SEKI_Lake-187", "SEKI_Lake-188", "SEKI_Lake-189", "SEKI_Lake-190",
"SEKI_Lake-191", "SEKI_Lake-192", "SEKI_Lake-193", "SEKI_Lake-194",
"SEKI_Lake-195", "SEKI_Lake-196", "SEKI_Lake-197", "SEKI_Lake-198",
"SEKI_Lake-199", "SEKI_Lake-200", "SEKI_Lake-201", "SEKI_Lake-202",
"SEKI_Lake-203", "SEKI_Lake-204", "SEKI_Lake-205", "SEKI_Lake-206",
"SEKI_Lake-207", "SEKI_Lake-208", "SEKI_Lake-209", "SEKI_Lake-210",
"SEKI_Lake-211", "SEKI_Lake-212", "SEKI_Lake-213", "SEKI_Lake-214",
```

```
"SEKI_Lake-215", "SEKI_Lake-216", "SEKI_Lake-217", "SEKI_Lake-218",
"SEKI_Lake-219", "SEKI_Lake-220", "SEKI_Lake-221", "SEKI_Lake-222",
"SEKI_Lake-223", "SEKI_Lake-224", "SEKI_Lake-225", "SEKI_Lake-226",
"SEKI_Lake-227", "SEKI_Lake-228", "SEKI_Lake-229", "SEKI_Lake-230",
"SEKI_Lake-231", "SEKI_Lake-232", "SEKI_Lake-233", "SEKI_Lake-234",
"SEKI_Lake-235", "SEKI_Lake-236", "SEKI_Lake-237", "SEKI_Lake-238",
"SEKI_Lake-239", "SEKI_Lake-240", "SEKI_Lake-241", "SEKI_Lake-242",
"SEKI_Lake-243", "SEKI_Lake-244", "SEKI_Lake-245", "SEKI_Lake-246",
"SEKI_Lake-247", "SEKI_Lake-248", "SEKI_Lake-249", "SEKI_Lake-250",
"SEKI_Lake-251", "SEKI_Lake-252", "SEKI_Lake-253", "SEKI_Lake-254",
"SEKI_Lake-255", "SEKI_Lake-256", "SEKI_Lake-257", "SEKI_Lake-258",
"SEKI_Lake-259", "SEKI_Lake-260", "SEKI_Lake-261", "SEKI_Lake-262",
"SEKI_Lake-263", "SEKI_Lake-264", "SEKI_Lake-265", "SEKI_Lake-266",
"SEKI_Lake-267", "SEKI_Lake-268", "SEKI_Lake-269", "SEKI_Lake-270",
"SEKI_Lake-271", "SEKI_Lake-272"), class = "factor"), Y = c(158.17,
151.02, 148.28, 124.04, 129.35, 160.28, 60.29, 37.37, 54.13,
50.52, 57.85, 39.65, 32.42, 36.79, 42.74, 45.24, 35.45, 37.82,
31.33, 41.01, 35.87, 56.77, 30.85, 38.46, 36.16, 40.38, 43.75,
40.76, 42.37, 63.09, 68.88, 91.36, 86.75, 149.98, 97.12, 49.5,
53.2, 58.2, 55.04, 128.94, 189.73, 80.65, 77.97, 54.31, 63.71,
51.16, 57.26, 52.91, 55.82, 42.22, 41.27, 90.06, 64.08, 83.02,
108.77, 45.27, 83.16, 38.95, 128.26, 14.53, 15.64, 47.96, 39.24,
69.44, 103.63, 66.63, 75.43, 53.25, 27.56, 92.14, 116.56, 34.21,
49.08, 59.61, 47.66, 23.99, 80.2, 14.82, 7.49, 15.65), Year = c(2013L,
2009L, 2010L, 2011L, 2008L, 2012L, 2009L, 2010L, 2012L, 2008L,
2013L, 2009L, 2010L, 2008L, 2012L, 2013L, 2011L, 2013L, 2011L,
2012L, 2008L, 2009L, 2010L, 2010L, 2011L, 2008L, 2009L, 2013L,
2012L, 2008L, 2012L, 2012L, 2008L, 2012L, 2008L, 2008L, 2012L,
2012L, 2008L, 2008L, 2012L, 2008L, 2012L, 2008L, 2012L, 2008L,
2012L, 2009L, 2009L, 2009L, 2009L, 2009L, 2009L, 2009L, 2009L,
2009L, 2009L, 2009L, 2010L, 2010L, 2010L, 2010L, 2010L, 2010L,
2010L, 2010L, 2010L, 2010L, 2011L, 2011L, 2011L, 2011L, 2011L,
2011L, 2011L, 2011L, 2011L, 2011L, 2008L, 2012L), WYear = c(5L,
1L, 2L, 3L, 0L, 4L, 1L, 2L, 4L, 0L, 5L, 1L, 2L, 0L, 4L, 5L, 3L,
5L, 3L, 4L, 0L, 1L, 2L, 2L, 3L, 0L, 1L, 5L, 4L, 0L, 4L, 4L, 0L,
4L, 0L, 0L, 4L, 4L, 0L, 0L, 4L, 0L, 4L, 0L, 4L, 0L, 4L, 1L, 1L,
1L, 1L, 1L, 1L, 1L, 1L, 1L, 1L, 1L, 2L, 2L, 2L, 2L, 2L, 2L, 2L,
2L, 2L, 2L, 3L, 3L, 3L, 3L, 3L, 3L, 3L, 3L, 3L, 3L, 0L, 4L),
    Panel = c(1L, 1L, 1L, 1L, 1L, 1L, 1L, 1L, 1L, 1L, 1L, 1L,
    1L, 1L, 1L, 1L, 1L, 1L, 1L, 1L, 1L, 1L, 1L, 1L, 1L, 1L, 1L,
    1L, 1L, 2L, 2L, 2L, 2L, 2L, 2L, 2L, 2L, 2L, 2L, 2L, 2L, 2L,
    2L, 2L, 2L, 2L, 2L, 3L, 3L, 3L, 3L, 3L, 3L, 3L, 3L, 3L, 3L,
    3L, 4L, 4L, 4L, 4L, 4L, 4L, 4L, 4L, 4L, 4L, 5L, 5L, 5L, 5L,
    5L, 5L, 5L, 5L, 5L, 5L, 2L, 2L), xcoord = c(367530.5077,
    367530.5077, 367530.5077, 367530.5077, 367530.5077, 367530.5077,
    346972.7465, 346972.7465, 346972.7465, 346972.7465, 346972.7465,
    360485.0803, 360485.0803, 360485.0803, 360485.0803, 360485.0803,
    360485.0803, 349828.7013, 349828.7013, 349828.7013, 349828.7013,
    349828.7013, 349828.7013, 360792.079, 360792.079, 360792.079,
    360792.079, 360792.079, 360792.079, 385954.4728, 385954.4728,
    373450.7546, 373450.7546, 360723.1753, 360723.1753, 355476.78,
    355476.78, 370678.6927, 370678.6927, 374456.832, 374456.832,
    348773.9394, 348773.9394, 355318.3521, 355318.3521, 371301.8329,
    371301.8329, 368889.9373, 349159.3927, 360942.1668, 346743.0753,
    359686.7779, 346479.4221, 363299.4485, 376304.4969, 362915.2972,
    350269.8602, 366488.6706, 376904.0391, 339565.2229, 357814.3612,
    377673.7317, 367309.9552, 354691.7723, 363862.1758, 364687.7547,
    370850.9014, 386563.9239, 364086.699, 372682.0972, 373230.9337,
    355550.6329, 381447.7036, 369414.0316, 364076.9102, 352210.7399,
    374447.9169, 370144.9149, 360321.7567, 360321.7567), ycoord = c(4093892.54,
    4093892.54, 4093892.54, 4093892.54, 4093892.54, 4093892.54,
    4112852.917, 4112852.917, 4112852.917, 4112852.917, 4112852.917,
    4037358.207, 4037358.207, 4037358.207, 4037358.207, 4037358.207,
```

```
4037358.207, 4052110.235, 4052110.235, 4052110.235, 4052110.235,
4052110.235, 4052110.235, 4086801.178, 4086801.178, 4086801.178,
4086801.178, 4086801.178, 4086801.178, 4043798.59, 4043798.59,
4068171.804, 4068171.804, 4106526.449, 4106526.449, 4029431.006,
4029431.006, 4051762.757, 4051762.757, 4059695.922, 4059695.922,
4116650.776, 4116650.776, 4031673.213, 4031673.213, 4083670.512,
4083670.512, 4097801.26, 4111198.837, 4036964.883, 4058212.006,
4090199.01, 4104358.637, 4046925.253, 4069918.456, 4108500.935,
4025111.1, 4045136.464, 4059978.772, 4114392.008, 4052347.39,
4081316.279, 4091976.547, 4104919.942, 4038755.818, 4065948.229,
4101667.288, 4044724.779, 4048635.721, 4070984.912, 4092091.495,
4080907.658, 4050627.729, 4075828.825, 4034665.855, 4052789.74,
4072716.1, 4088550.993, 4044132.817, 4044132.817), lake_eleva = c(3370L,
3370L, 3370L, 3370L, 3370L, 3370L, 3297L, 3297L, 3297L, 3297L,
3297L, 3066L, 3066L, 3066L, 3066L, 3066L, 3066L, 2774L, 2774L,
2774L, 2774L, 2774L, 2774L, 3359L, 3359L, 3359L, 3359L, 3359L,
3359L, 3519L, 3519L, 3150L, 3150L, 3274L, 3274L, 3115L, 3115L,
3510L, 3510L, 3650L, 3650L, 3408L, 3408L, 3018L, 3018L, 3150L,
3150L, 3539L, 3442L, 3188L, 2871L, 3200L, 3359L, 3481L, 3220L,
3627L, 2615L, 3510L, 3710L, 3347L, 3287L, 3478L, 3400L, 3405L,
3127L, 3205L, 3545L, 3750L, 3469L, 3165L, 3478L, 3076L, 3570L,
3214L, 3310L, 3139L, 3450L, 3469L, 3456L, 3456L), categories = structure(c(5L,
5L, 5L, 5L, 5L, 5L, 3L, 3L, 3L, 3L, 3L, 1L, 1L, 1L, 1L, 1L,
1L, 1L, 1L, 1L, 1L, 1L, 1L, 4L, 4L, 4L, 4L, 4L, 4L, 3L, 3L,
2L, 2L, 2L, 2L, 1L, 1L, 6L, 6L, 5L, 5L, 2L, 2L, 1L, 1L, 3L,
3L, 3L, 3L, 1L, 1L, 4L, 5L, 4L, 1L, 1L, 2L, 6L, 4L, 3L, 2L,
4L, 4L, 4L, 2L, 3L, 5L, 3L, 5L, 2L, 3L, 2L, 5L, 3L, 3L, 1L,
3L, 4L, 3L, 3L), .Label = c("1", "2", "3", "4", "5", "6"), class = "factor"),
inver_cost = c(0.08, 0.08, 0.08, 0.08, 0.08, 0.08, 0.107,
0.107, 0.107, 0.107, 0.107, 0.245, 0.245, 0.245, 0.245, 0.245,
0.245, 0.364, 0.364, 0.364, 0.364, 0.364, 0.364, 0.084, 0.084,
0.084, 0.084, 0.084, 0.084, 0.121, 0.121, 0.143, 0.143, 0.175,
0.175, 0.259, 0.259, 0.055, 0.055, 0.074, 0.074, 0.15, 0.15,
0.392, 0.392, 0.104, 0.104, 0.096, 0.106, 0.202, 0.242, 0.085,
0.069, 0.085, 0.232, 0.223, 0.151, 0.058, 0.084, 0.107, 0.13,
0.081, 0.089, 0.082, 0.135, 0.124, 0.075, 0.111, 0.073, 0.174,
0.121, 0.126, 0.08, 0.102, 0.124, 0.244, 0.118, 0.092, 0.096,
0.096), Elev_Stratum = c("High", "High", "High", "High",
"High", "High", "High", "High", "High", "High", "High", "Low",
"Low", "Low", "Low", "Low", "Low", "Low", "Low", "Low", "Low",
"Low", "Low", "High", "High", "High", "High", "High", "High",
"High", "High", "High", "High", "High", "High", "High", "High",
"High", "High", "High", "High", "High", "High", "Low", "Low",
"High", "High", "High", "High", "High", "Low", "High", "High",
"High", "High", "High", "Low", "High", "High", "High", "High",
"High", "High", "High", "High", "High", "High", "High", "High",
"High", "High", "Low", "High", "High", "High", "High", "High",
"High", "High", "High"), wgt = c(21.2765957446809, 21.2765957446809,
21.2765957446809, 21.2765957446809, 21.2765957446809, 21.2765957446809,
21.2765957446809, 21.2765957446809, 21.2765957446809, 21.2765957446809,
21.2765957446809, 2.80112044817927, 2.80112044817927, 2.80112044817927,
2.80112044817927, 2.80112044817927, 2.80112044817927, 2.80112044817927,
2.80112044817927, 2.80112044817927, 2.80112044817927, 2.80112044817927,
2.80112044817927, 24.390243902439, 24.390243902439, 24.390243902439,
24.390243902439, 24.390243902439, 24.390243902439, 21.2765957446809,
21.2765957446809, 4.85436893203884, 4.85436893203884, 4.85436893203884,
4.85436893203884, 4.85436893203884, 4.85436893203884, 21.2765957446809,
21.2765957446809, 24.390243902439, 24.390243902439, 4.85436893203884,
4.85436893203884, 2.80112044817927, 2.80112044817927, 21.2765957446809,
21.2765957446809, 21.2765957446809, 21.2765957446809, 4.85436893203884,
4.85436893203884, 24.390243902439, 21.2765957446809, 24.390243902439,
2.80112044817927, 2.80112044817927, 4.85436893203884, 21.2765957446809,
24.390243902439, 21.2765957446809, 21.2765957446809, 21.2765957446809,
```

```
    21.2765957446809, 24.390243902439, 21.2765957446809, 21.2765957446809,
    24.390243902439, 21.2765957446809, 24.390243902439, 4.85436893203884,
    4.85436893203884, 21.2765957446809, 24.390243902439, 24.390243902439,
    21.2765957446809, 4.85436893203884, 21.2765957446809, 21.2765957446809,
    24.390243902439, 24.390243902439), LogY = c(5.0636704039754,
    5.01741227837528, 4.99910237828253, 4.82060409423225, 4.86252190863204,
    5.07692228576794, 4.09916625250131, 3.62086824349739, 3.99138856080889,
    3.92236929748229, 4.05785345363969, 3.68009094808086, 3.47877551630753,
    3.60522606911668, 3.755135249891, 3.81198165124792, 3.56812325297814,
    3.63283806323031, 3.4445761049641, 3.71381593940397, 3.57990129154419,
    4.03900801718263, 3.4291367503514, 3.64961874016063, 3.58795353552398,
    3.69833461288469, 3.77849161280362, 3.70770120835452, 3.74644056463847,
    4.14456227838272, 4.23236586011947, 4.5148077459077, 4.46303041882697,
    5.01050195187324, 4.57594732731119, 3.90197266957464, 3.9740583963476,
    4.06388535473739, 4.00806019362509, 4.85934717988247, 5.24560200887604,
    4.39011880457122, 4.35632413732151, 3.99470837204675, 4.1543415361284,
    3.93495797671064, 4.04760229966997, 3.96859235691604, 4.02213222810818,
    3.74289404242017, 3.72013584376449, 4.50047611487343, 4.1601323027601,
    4.41908154262489, 4.68923556110576, 3.81264456144787, 4.42076646298981,
    3.66227877231676, 4.85405945372072, 2.67621547758219, 2.74983173511717,
    3.87036733015931, 3.66969663469716, 4.24046307035209, 4.64082686319613,
    4.19915492657445, 4.32320507386554, 3.97499780458953, 3.31636544614546,
    4.52330915950777, 4.75840616188695, 3.53251799898951, 3.89345161984271,
    4.08782334523184, 3.8640924716706, 3.1776370768516, 4.38452351487247,
    2.69597761986794, 2.01356879752913, 2.75047091698616)), .Names = c("Site",
"Y", "Year", "WYear", "Panel", "xcoord", "ycoord", "lake_eleva",
"categories", "inver_cost", "Elev_Stratum", "wgt", "LogY"), row.names = c(NA,
80L), class = "data.frame")

# Estimate trend for pilot data with all approaches
require(lme4)
require(spsurvey)
require(lmerTest)
TrendEsts(SEKIANC_Example, "Unequal")
```
