# Supplementary material for: Trend estimation for complex survey designs of water chemistry indicators from Sierra Nevada Lakes
Source: Environ Monit Assess. 2018 Sep 19;190(10):596. doi: 10.1007/s10661-018-6963-1 (PMC6153522; doi:10.1007/s10661-018-6963-1)
Supplement: Supplementary file 3 — (PDF 3585 kb) [file 10661_2018_6963_MOESM3_ESM.pdf]

Supplement C: simulation results

Table B1: Relative Bias of the ANC Trend Slope Estimate (shaded cells indicate > 5% or < -5% relative bias)

Years	Trend (p)	Subpop Trend	True Slope	Sampling Design	Revisit Design	Sites	PO	SLRDB	WLRDB	PWIGLS A-only	PWIGLS A	PWIGLS AI	PWIGLS B	PWIGLS BI	PWIGLS C
12	0.02	0	0.02	SRS	1-0	20	-0.65%	4.83%	0.41%	-0.65%	-0.65%	-0.65%	-0.65%	-0.65%	-0.65%
12	0.02	0	0.02	SRS	1-0	35	-0.79%	3.91%	0.72%	-0.79%	-0.79%	-0.79%	-0.79%	-0.79%	-0.79%
12	0.02	0	0.02	SRS	1-0	50	-0.68%	4.39%	0.94%	-0.68%	-0.68%	-0.68%	-0.68%	-0.68%	-0.68%
12	0.02	0	0.02	SRS	1-0,1-3	20	5.00%	8.69%	5.57%	4.44%	5.00%	4.33%	5.00%	4.33%	4.44%
12	0.02	0	0.02	SRS	1-0,1-3	35	4.17%	10.53%	5.37%	5.65%	4.17%	5.66%	4.17%	5.66%	5.65%
12	0.02	0	0.02	SRS	1-0,1-3	50	3.25%	8.36%	5.67%	4.65%	3.25%	4.72%	3.25%	4.72%	4.65%
12	0.02	0	0.02	SRS	1-3	20	-1.34%	4.84%	4.59%	-1.38%	-1.38%	-1.38%	-1.38%	-1.38%	-1.38%
12	0.02	0	0.02	SRS	1-3	35	-1.65%	3.22%	5.77%	-1.63%	-1.63%	-1.63%	-1.63%	-1.63%	-1.63%
12	0.02	0	0.02	SRS	1-3	50	-1.70%	3.78%	4.73%	-1.70%	-1.70%	-1.70%	-1.70%	-1.70%	-1.70%
12	0.02	0	0.02	StRS	1-0	20	-1.42%	2.90%	6.36%	-3.66%	-3.66%	-3.66%	-3.66%	-3.66%	-3.66%
12	0.02	0	0.02	StRS	1-0	35	-0.33%	3.90%	4.88%	-3.62%	-3.62%	-3.62%	-3.62%	-3.62%	-3.62%
12	0.02	0	0.02	StRS	1-0	50	-0.93%	4.68%	6.00%	-2.20%	-2.20%	-2.20%	-2.20%	-2.20%	-2.20%
12	0.02	0	0.02	StRS	1-0,1-3	20	-0.05%	3.57%	-0.55%	-1.45%	-3.01%	-1.38%	-3.01%	-1.38%	-1.45%
12	0.02	0	0.02	StRS	1-0,1-3	35	0.53%	5.85%	0.81%	-1.53%	-2.66%	-1.47%	-2.66%	-1.47%	-1.53%
12	0.02	0	0.02	StRS	1-0,1-3	50	1.60%	8.16%	0.25%	1.19%	-0.24%	1.18%	-0.24%	1.18%	1.19%
12	0.02	0	0.02	StRS	1-3	20	-1.91%	0.02%	6.03%	-4.90%	-4.72%	-4.90%	-4.72%	-4.90%	-4.72%
12	0.02	0	0.02	StRS	1-3	35	-2.80%	2.63%	4.89%	-6.08%	-6.09%	-6.09%	-6.09%	-6.09%	-6.09%
12	0.02	0	0.02	StRS	1-3	50	-1.98%	4.08%	8.79%	-6.25%	-6.25%	-6.25%	-6.25%	-6.25%	-6.25%
12	0.02	0	0.02	Unequal	1-0	20	0.11%	5.37%	3.27%	0.55%	0.55%	0.55%	0.55%	0.55%	0.55%
12	0.02	0	0.02	Unequal	1-0	35	0.59%	6.23%	4.49%	1.04%	1.04%	1.04%	1.04%	1.04%	1.04%
12	0.02	0	0.02	Unequal	1-0	50	0.82%	6.09%	2.24%	1.80%	1.80%	1.80%	1.80%	1.80%	1.80%
12	0.02	0	0.02	Unequal	1-0,1-3	20	1.46%	5.80%	0.76%	3.79%	1.51%	3.75%	1.51%	3.75%	3.79%
12	0.02	0	0.02	Unequal	1-0,1-3	35	2.48%	5.07%	5.24%	2.76%	1.90%	2.81%	1.90%	2.81%	2.76%
12	0.02	0	0.02	Unequal	1-0,1-3	50	2.61%	7.17%	5.67%	2.12%	2.32%	2.15%	2.32%	2.15%	2.12%
12	0.02	0	0.02	Unequal	1-3	20	1.07%	3.87%	4.53%	0.28%	0.28%	0.28%	0.28%	0.28%	0.28%
12	0.02	0	0.02	Unequal	1-3	35	0.70%	3.17%	2.18%	1.93%	1.93%	1.93%	1.93%	1.93%	1.93%
12	0.02	0	0.02	Unequal	1-3	50	1.09%	6.18%	2.08%	2.58%	2.58%	2.58%	2.58%	2.58%	2.58%
12	0.02	0.04	0.05	SRS	1-0	20	-1.37%	2.30%	3.02%	-1.37%	-1.37%	-1.37%	-1.37%	-1.37%	-1.37%
12	0.02	0.04	0.05	SRS	1-0	35	-0.95%	2.27%	2.30%	-0.95%	-0.95%	-0.95%	-0.95%	-0.95%	-0.95%
12	0.02	0.04	0.05	SRS	1-0	50	-1.22%	2.09%	2.55%	-1.22%	-1.22%	-1.22%	-1.22%	-1.22%	-1.22%
12	0.02	0.04	0.05	SRS	1-0,1-3	20	-0.71%	2.50%	3.09%	-0.36%	-0.71%	-0.49%	-0.71%	-0.49%	-0.36%
12	0.02	0.04	0.05	SRS	1-0,1-3	35	-0.71%	2.58%	4.41%	0.30%	-0.71%	-0.04%	-0.71%	-0.04%	0.30%
12	0.02	0.04	0.05	SRS	1-0,1-3	50	-0.57%	2.41%	3.46%	-0.23%	-0.57%	-0.44%	-0.57%	-0.44%	-0.23%
12	0.02	0.04	0.05	SRS	1-3	20	-2.90%	0.16%	3.00%	-2.89%	-2.89%	-2.89%	-2.89%	-2.89%	-2.89%
12	0.02	0.04	0.05	SRS	1-3	35	-2.12%	1.37%	3.97%	-2.11%	-2.11%	-2.11%	-2.11%	-2.11%	-2.11%
12	0.02	0.04	0.05	SRS	1-3	50	-1.73%	1.96%	2.86%	-1.76%	-1.76%	-1.76%	-1.76%	-1.76%	-1.76%

Table B1: Relative Bias of the ANC Trend Slope Estimate (shaded cells indicate > 5% or < -5% relative bias)

Years	Trend (p)	Subpop Trend	True Slope	Sampling Design	Revisit Design	Sites	PO	SLRDB	WLRDB	PWIGLS A-only	PWIGLS A	PWIGLS AI	PWIGLS B	PWIGLS BI	PWIGLS C
12	0.02	0.04	0.05	StRS	1-0	20	0.95%	6.79%	3.51%	0.95%	0.95%	0.95%	0.95%	0.95%	0.95%
12	0.02	0.04	0.05	StRS	1-0	35	1.19%	7.52%	3.47%	1.19%	1.19%	1.19%	1.19%	1.19%	1.19%
12	0.02	0.04	0.05	StRS	1-0	50	1.25%	7.35%	4.15%	1.25%	1.25%	1.25%	1.25%	1.25%	1.25%
12	0.02	0.04	0.05	StRS	1-0,1-3	20	0.95%	6.77%	2.10%	1.13%	0.98%	1.11%	0.98%	1.11%	1.13%
12	0.02	0.04	0.05	StRS	1-0,1-3	35	1.20%	6.90%	3.03%	1.02%	1.23%	1.02%	1.23%	1.02%	1.02%
12	0.02	0.04	0.05	StRS	1-0,1-3	50	1.39%	7.45%	3.24%	1.06%	1.41%	1.09%	1.41%	1.09%	1.06%
12	0.02	0.04	0.05	StRS	1-3	20	0.47%	6.82%	1.96%	0.36%	0.36%	0.36%	0.36%	0.36%	0.36%
12	0.02	0.04	0.05	StRS	1-3	35	0.27%	5.55%	2.33%	0.20%	0.20%	0.20%	0.20%	0.20%	0.20%
12	0.02	0.04	0.05	StRS	1-3	50	0.08%	5.51%	3.26%	0.02%	0.02%	0.02%	0.02%	0.02%	0.02%
12	0.02	0.04	0.05	Unequal	1-0	20	-7.38%	2.91%	0.60%	4.49%	4.49%	4.49%	4.49%	4.49%	4.49%
12	0.02	0.04	0.05	Unequal	1-0	35	-6.25%	3.81%	1.67%	7.65%	7.65%	7.65%	7.65%	7.65%	7.65%
12	0.02	0.04	0.05	Unequal	1-0	50	-7.19%	3.92%	1.75%	9.21%	9.21%	9.21%	9.21%	9.21%	9.21%
12	0.02	0.04	0.05	Unequal	1-0,1-3	20	-6.53%	2.91%	-0.19%	0.78%	6.84%	-0.11%	6.84%	-0.11%	0.78%
12	0.02	0.04	0.05	Unequal	1-0,1-3	35	-6.46%	5.18%	2.20%	3.58%	11.15%	3.77%	11.15%	3.77%	3.58%
12	0.02	0.04	0.05	Unequal	1-0,1-3	50	-6.68%	3.76%	1.92%	4.92%	11.74%	5.96%	11.74%	5.96%	4.92%
12	0.02	0.04	0.05	Unequal	1-3	20	-5.57%	4.66%	-1.32%	9.87%	9.87%	9.87%	9.87%	9.87%	9.87%
12	0.02	0.04	0.05	Unequal	1-3	35	-5.58%	4.19%	0.76%	11.94%	11.94%	11.94%	11.94%	11.94%	11.94%
12	0.02	0.04	0.05	Unequal	1-3	50	-5.80%	4.51%	-0.36%	14.15%	14.15%	14.15%	14.15%	14.15%	14.15%
24	0.01	0	0.01	SRS	1-0	20	1.64%	17.24%	17.34%	1.64%	1.64%	1.64%	1.64%	1.64%	1.64%
24	0.01	0	0.01	SRS	1-0	35	2.09%	18.11%	18.29%	2.09%	2.09%	2.09%	2.09%	2.09%	2.09%
24	0.01	0	0.01	SRS	1-0	50	0.85%	17.30%	15.68%	0.85%	0.85%	0.85%	0.85%	0.85%	0.85%
24	0.01	0	0.01	SRS	1-0,1-3	20	-1.12%	14.56%	12.81%	-0.38%	-1.13%	-0.37%	-1.13%	-0.37%	-0.38%
24	0.01	0	0.01	SRS	1-0,1-3	35	-0.90%	14.89%	14.82%	-1.62%	-0.90%	-1.39%	-0.90%	-1.39%	-1.62%
24	0.01	0	0.01	SRS	1-0,1-3	50	-0.91%	15.60%	16.04%	0.70%	-0.91%	-0.06%	-0.91%	-0.06%	0.70%
24	0.01	0	0.01	SRS	1-3	20	0.40%	15.05%	12.01%	0.40%	0.40%	0.40%	0.40%	0.40%	0.40%
24	0.01	0	0.01	SRS	1-3	35	1.21%	17.67%	12.32%	1.19%	1.19%	1.19%	1.19%	1.19%	1.19%
24	0.01	0	0.01	SRS	1-3	50	0.41%	16.79%	13.69%	0.41%	0.41%	0.41%	0.41%	0.41%	0.41%
24	0.01	0	0.01	StRS	1-0	20	-0.57%	16.17%	14.22%	0.82%	0.82%	0.82%	0.82%	0.82%	0.82%
24	0.01	0	0.01	StRS	1-0	35	1.63%	18.84%	14.18%	2.56%	2.56%	2.56%	2.56%	2.56%	2.56%
24	0.01	0	0.01	StRS	1-0	50	-0.50%	15.88%	13.36%	0.02%	0.02%	0.02%	0.02%	0.02%	0.02%
24	0.01	0	0.01	StRS	1-0,1-3	20	0.57%	15.98%	11.50%	-0.65%	-0.91%	-0.30%	-0.91%	-0.30%	-0.65%
24	0.01	0	0.01	StRS	1-0,1-3	35	1.10%	16.59%	11.97%	0.80%	1.01%	0.85%	1.01%	0.85%	0.80%
24	0.01	0	0.01	StRS	1-0,1-3	50	0.70%	17.18%	10.20%	1.18%	0.93%	1.11%	0.93%	1.11%	1.18%
24	0.01	0	0.01	StRS	1-3	20	1.28%	17.37%	9.40%	2.94%	2.94%	2.94%	2.94%	2.94%	2.94%
24	0.01	0	0.01	StRS	1-3	35	1.05%	17.43%	11.01%	2.61%	2.61%	2.61%	2.61%	2.61%	2.61%
24	0.01	0	0.01	StRS	1-3	50	-0.08%	17.56%	11.84%	1.66%	1.66%	1.66%	1.66%	1.66%	1.66%

Table B1: Relative Bias of the ANC Trend Slope Estimate (shaded cells indicate > 5% or < -5% relative bias)

Years	Trend (p)	Subpop Trend	True Slope	Sampling Design	Revisit Design	Sites	PO	SLRDB	WLRDB	PWIGLS A-only	PWIGLS A	PWIGLS AI	PWIGLS B	PWIGLS BI	PWIGLS C
24	0.01	0	0.01	Unequal	1-0	20	-1.31%	12.75%	18.16%	-2.18%	-2.18%	-2.18%	-2.18%	-2.18%	-2.18%
24	0.01	0	0.01	Unequal	1-0	35	-1.08%	16.21%	17.34%	1.42%	1.42%	1.42%	1.42%	1.42%	1.42%
24	0.01	0	0.01	Unequal	1-0	50	-0.36%	15.99%	16.82%	0.60%	0.60%	0.60%	0.60%	0.60%	0.60%
24	0.01	0	0.01	Unequal	1-0,1-3	20	0.12%	12.87%	10.66%	-0.34%	-0.57%	0.11%	-0.57%	0.11%	-0.34%
24	0.01	0	0.01	Unequal	1-0,1-3	35	0.79%	14.35%	10.72%	0.24%	-0.20%	0.00%	-0.20%	0.00%	0.24%
24	0.01	0	0.01	Unequal	1-0,1-3	50	-0.28%	16.32%	12.60%	0.14%	0.26%	0.13%	0.26%	0.13%	0.14%
24	0.01	0	0.01	Unequal	1-3	20	2.43%	16.85%	4.44%	3.23%	3.23%	3.23%	3.23%	3.23%	3.23%
24	0.01	0	0.01	Unequal	1-3	35	2.43%	17.05%	6.72%	0.34%	0.34%	0.34%	0.34%	0.34%	0.34%
24	0.01	0	0.01	Unequal	1-3	50	2.14%	16.82%	5.83%	2.54%	2.54%	2.54%	2.54%	2.54%	2.54%
24	0.01	0.04	0.04	SRS	1-0	20	0.01%	6.96%	5.15%	0.01%	0.01%	0.01%	0.01%	0.01%	0.01%
24	0.01	0.04	0.04	SRS	1-0	35	0.02%	7.46%	5.37%	0.02%	0.02%	0.02%	0.02%	0.02%	0.02%
24	0.01	0.04	0.04	SRS	1-0	50	0.16%	7.24%	5.65%	0.16%	0.16%	0.16%	0.16%	0.16%	0.16%
24	0.01	0.04	0.04	SRS	1-0,1-3	20	-0.15%	6.59%	4.91%	-0.06%	-0.15%	-0.05%	-0.15%	-0.05%	-0.06%
24	0.01	0.04	0.04	SRS	1-0,1-3	35	0.22%	6.99%	5.29%	0.34%	0.22%	0.35%	0.22%	0.35%	0.34%
24	0.01	0.04	0.04	SRS	1-0,1-3	50	0.02%	6.93%	5.20%	0.19%	0.02%	0.16%	0.02%	0.16%	0.19%
24	0.01	0.04	0.04	SRS	1-3	20	0.03%	6.78%	3.19%	0.03%	0.03%	0.03%	0.03%	0.03%	0.03%
24	0.01	0.04	0.04	SRS	1-3	35	-0.21%	6.72%	4.08%	-0.22%	-0.22%	-0.22%	-0.22%	-0.22%	-0.22%
24	0.01	0.04	0.04	SRS	1-3	50	0.09%	7.03%	4.51%	0.09%	0.09%	0.09%	0.09%	0.09%	0.09%
24	0.01	0.04	0.04	StRS	1-0	20	-0.36%	9.07%	7.78%	-0.36%	-0.36%	-0.36%	-0.36%	-0.36%	-0.36%
24	0.01	0.04	0.04	StRS	1-0	35	-0.05%	9.66%	7.89%	-0.05%	-0.05%	-0.05%	-0.05%	-0.05%	-0.05%
24	0.01	0.04	0.04	StRS	1-0	50	-0.18%	9.78%	7.32%	-0.18%	-0.18%	-0.18%	-0.18%	-0.18%	-0.18%
24	0.01	0.04	0.04	StRS	1-0,1-3	20	0.14%	9.58%	6.49%	0.09%	0.15%	0.08%	0.15%	0.08%	0.09%
24	0.01	0.04	0.04	StRS	1-0,1-3	35	0.21%	9.95%	5.91%	0.20%	0.21%	0.21%	0.21%	0.21%	0.20%
24	0.01	0.04	0.04	StRS	1-0,1-3	50	0.14%	9.99%	6.81%	0.12%	0.13%	0.10%	0.13%	0.10%	0.12%
24	0.01	0.04	0.04	StRS	1-3	20	-0.94%	8.63%	6.34%	-0.93%	-0.93%	-0.93%	-0.93%	-0.93%	-0.93%
24	0.01	0.04	0.04	StRS	1-3	35	-0.69%	9.20%	6.48%	-0.70%	-0.70%	-0.70%	-0.70%	-0.70%	-0.70%
24	0.01	0.04	0.04	StRS	1-3	50	-0.86%	9.00%	6.96%	-0.86%	-0.86%	-0.86%	-0.86%	-0.86%	-0.86%
24	0.01	0.04	0.04	Unequal	1-0	20	-9.37%	5.46%	3.48%	-0.44%	-0.44%	-0.44%	-0.44%	-0.44%	-0.44%
24	0.01	0.04	0.04	Unequal	1-0	35	-9.22%	6.51%	4.77%	1.92%	1.92%	1.92%	1.92%	1.92%	1.92%
24	0.01	0.04	0.04	Unequal	1-0	50	-9.01%	6.56%	5.26%	2.89%	2.89%	2.89%	2.89%	2.89%	2.89%
24	0.01	0.04	0.04	Unequal	1-0,1-3	20	-9.66%	4.55%	3.21%	-0.85%	1.31%	-1.46%	1.31%	-1.46%	-0.85%
24	0.01	0.04	0.04	Unequal	1-0,1-3	35	-9.36%	5.92%	3.53%	1.65%	4.92%	1.43%	4.92%	1.43%	1.65%
24	0.01	0.04	0.04	Unequal	1-0,1-3	50	-9.75%	6.19%	3.86%	2.51%	6.12%	2.55%	6.12%	2.55%	2.51%
24	0.01	0.04	0.04	Unequal	1-3	20	-8.80%	6.38%	0.72%	3.97%	3.97%	3.97%	3.97%	3.97%	3.97%
24	0.01	0.04	0.04	Unequal	1-3	35	-8.74%	6.76%	1.67%	6.40%	6.40%	6.40%	6.40%	6.40%	6.40%
24	0.01	0.04	0.04	Unequal	1-3	50	-9.02%	6.37%	2.59%	8.42%	8.42%	8.42%	8.42%	8.42%	8.42%

Table B2: Relative Bias of the modified ANC Trend Slope Estimate (shaded cells indicate > 5% or < -5% relative bias)

Years	Trend (p)	Subpop Trend	True Slope	Sampling Design	Revisit Design	Sites	PO	SLRDB	WLRDB	PWIGLS A-only	PWIGLS A	PWIGLS AI	PWIGLS B	PWIGLS BI	PWIGLS C
12	0.02	0	0.02	SRS	1-0	20	1.38%	6.28%	5.58%	1.38%	1.38%	1.38%	1.38%	1.38%	1.38%
12	0.02	0	0.02	SRS	1-0	35	0.31%	5.74%	4.75%	0.31%	0.31%	0.31%	0.31%	0.31%	0.31%
12	0.02	0	0.02	SRS	1-0	50	-0.02%	5.64%	4.71%	-0.02%	-0.02%	-0.02%	-0.02%	-0.02%	-0.02%
12	0.02	0	0.02	SRS	1-0,1-3	20	-2.03%	2.74%	1.26%	-2.08%	-2.03%	-2.14%	-2.03%	-2.14%	-2.08%
12	0.02	0	0.02	SRS	1-0,1-3	35	-0.54%	5.14%	2.89%	-0.56%	-0.54%	-0.52%	-0.54%	-0.52%	-0.56%
12	0.02	0	0.02	SRS	1-0,1-3	50	-0.21%	5.12%	3.35%	-0.24%	-0.22%	-0.28%	-0.22%	-0.28%	-0.24%
12	0.02	0	0.02	SRS	1-3	20	0.40%	6.47%	-1.97%	0.40%	0.40%	0.40%	0.40%	0.40%	0.40%
12	0.02	0	0.02	SRS	1-3	35	0.03%	4.70%	3.00%	0.03%	0.03%	0.03%	0.03%	0.03%	0.03%
12	0.02	0	0.02	SRS	1-3	50	0.51%	4.49%	4.67%	0.52%	0.52%	0.52%	0.52%	0.52%	0.52%
12	0.02	0	0.02	StRS	1-0	20	0.30%	5.51%	5.52%	0.12%	0.12%	0.12%	0.12%	0.12%	0.12%
12	0.02	0	0.02	StRS	1-0	35	0.16%	4.99%	5.15%	-0.13%	-0.13%	-0.13%	-0.13%	-0.13%	-0.13%
12	0.02	0	0.02	StRS	1-0	50	0.14%	5.18%	4.89%	0.16%	0.16%	0.16%	0.16%	0.16%	0.16%
12	0.02	0	0.02	StRS	1-0,1-3	20	0.64%	5.85%	3.91%	0.43%	0.41%	0.50%	0.41%	0.50%	0.43%
12	0.02	0	0.02	StRS	1-0,1-3	35	-0.32%	3.21%	4.11%	-0.57%	-0.55%	-0.56%	-0.55%	-0.56%	-0.57%
12	0.02	0	0.02	StRS	1-0,1-3	50	-0.02%	6.10%	3.59%	-0.16%	-0.20%	-0.10%	-0.20%	-0.10%	-0.16%
12	0.02	0	0.02	StRS	1-3	20	-0.47%	5.49%	2.43%	-0.49%	-0.49%	-0.49%	-0.49%	-0.49%	-0.49%
12	0.02	0	0.02	StRS	1-3	35	-0.51%	2.47%	2.28%	-0.55%	-0.55%	-0.55%	-0.55%	-0.55%	-0.55%
12	0.02	0	0.02	StRS	1-3	50	-0.22%	4.72%	3.33%	-0.30%	-0.30%	-0.30%	-0.30%	-0.30%	-0.30%
12	0.02	0	0.02	Unequal	1-0	20	1.06%	5.84%	1.73%	1.18%	1.18%	1.18%	1.18%	1.18%	1.18%
12	0.02	0	0.02	Unequal	1-0	35	0.76%	4.58%	3.72%	0.39%	0.39%	0.39%	0.39%	0.39%	0.39%
12	0.02	0	0.02	Unequal	1-0	50	0.53%	5.87%	5.15%	0.46%	0.46%	0.46%	0.46%	0.46%	0.46%
12	0.02	0	0.02	Unequal	1-0,1-3	20	-0.76%	1.74%	4.62%	-1.02%	-1.01%	-0.89%	-1.01%	-0.89%	-1.02%
12	0.02	0	0.02	Unequal	1-0,1-3	35	-0.35%	4.96%	5.52%	-0.57%	-0.61%	-0.55%	-0.61%	-0.55%	-0.57%
12	0.02	0	0.02	Unequal	1-0,1-3	50	0.43%	6.63%	3.24%	0.38%	0.49%	0.32%	0.49%	0.32%	0.38%
12	0.02	0	0.02	Unequal	1-3	20	0.09%	5.12%	-0.19%	-0.05%	-0.05%	-0.05%	-0.05%	-0.05%	-0.05%
12	0.02	0	0.02	Unequal	1-3	35	-0.05%	1.64%	0.49%	-0.11%	-0.11%	-0.11%	-0.11%	-0.11%	-0.11%
12	0.02	0	0.02	Unequal	1-3	50	0.05%	4.63%	3.92%	-0.06%	-0.06%	-0.06%	-0.06%	-0.06%	-0.06%
12	0.02	0.04	0.05	SRS	1-0	20	0.06%	3.12%	2.58%	0.06%	0.06%	0.06%	0.06%	0.06%	0.06%
12	0.02	0.04	0.05	SRS	1-0	35	0.19%	3.57%	3.12%	0.19%	0.19%	0.19%	0.19%	0.19%	0.19%
12	0.02	0.04	0.05	SRS	1-0	50	0.09%	3.45%	3.01%	0.09%	0.09%	0.09%	0.09%	0.09%	0.09%
12	0.02	0.04	0.05	SRS	1-0,1-3	20	-0.03%	3.18%	1.87%	-0.04%	-0.02%	0.14%	-0.02%	0.14%	-0.04%
12	0.02	0.04	0.05	SRS	1-0,1-3	35	0.21%	3.77%	2.30%	0.21%	0.22%	0.28%	0.22%	0.28%	0.22%
12	0.02	0.04	0.05	SRS	1-0,1-3	50	0.00%	3.34%	3.07%	0.01%	0.01%	0.09%	0.01%	0.09%	0.01%
12	0.02	0.04	0.05	SRS	1-3	20	0.22%	3.31%	2.04%	0.22%	0.22%	0.22%	0.22%	0.22%	0.22%
12	0.02	0.04	0.05	SRS	1-3	35	0.19%	3.55%	2.52%	0.19%	0.19%	0.19%	0.19%	0.19%	0.19%
12	0.02	0.04	0.05	SRS	1-3	50	0.11%	3.13%	2.51%	0.11%	0.11%	0.11%	0.11%	0.11%	0.11%

Table B2: Relative Bias of the modified ANC Trend Slope Estimate (shaded cells indicate > 5% or < -5% relative bias)

Years	Trend (p)	Subpop Trend	True Slope	Sampling Design	Revisit Design	Sites	PO	SLRDB	WLRDB	PWIGLS A-only	PWIGLS A	PWIGLS AI	PWIGLS B	PWIGLS BI	PWIGLS C
12	0.02	0.04	0.05	StRS	1-0	20	0.21%	6.10%	5.60%	0.21%	0.21%	0.21%	0.21%	0.21%	0.21%
12	0.02	0.04	0.05	StRS	1-0	35	-0.10%	6.05%	5.93%	-0.10%	-0.10%	-0.10%	-0.10%	-0.10%	-0.10%
12	0.02	0.04	0.05	StRS	1-0	50	-0.02%	6.18%	6.10%	-0.02%	-0.02%	-0.02%	-0.02%	-0.02%	-0.02%
12	0.02	0.04	0.05	StRS	1-0,1-3	20	-0.03%	6.07%	4.84%	-0.06%	-0.05%	-0.02%	-0.05%	-0.02%	-0.06%
12	0.02	0.04	0.05	StRS	1-0,1-3	35	-0.20%	5.38%	4.51%	-0.22%	-0.22%	-0.21%	-0.22%	-0.21%	-0.22%
12	0.02	0.04	0.05	StRS	1-0,1-3	50	-0.04%	6.58%	5.84%	-0.03%	-0.03%	-0.04%	-0.03%	-0.04%	-0.03%
12	0.02	0.04	0.05	StRS	1-3	20	0.49%	6.04%	4.65%	0.48%	0.49%	0.49%	0.49%	0.49%	0.49%
12	0.02	0.04	0.05	StRS	1-3	35	0.37%	5.45%	4.64%	0.37%	0.37%	0.37%	0.37%	0.37%	0.37%
12	0.02	0.04	0.05	StRS	1-3	50	0.05%	7.13%	5.22%	0.05%	0.05%	0.05%	0.05%	0.05%	0.05%
12	0.02	0.04	0.05	Unequal	1-0	20	-7.54%	1.80%	0.85%	-2.69%	-2.69%	-2.69%	-2.69%	-2.69%	-2.69%
12	0.02	0.04	0.05	Unequal	1-0	35	-6.55%	3.17%	2.23%	-1.46%	-1.46%	-1.46%	-1.46%	-1.46%	-1.46%
12	0.02	0.04	0.05	Unequal	1-0	50	-7.55%	3.06%	2.34%	-2.36%	-2.36%	-2.36%	-2.36%	-2.36%	-2.36%
12	0.02	0.04	0.05	Unequal	1-0,1-3	20	-7.39%	2.84%	0.88%	-3.89%	-3.77%	-4.69%	-3.77%	-4.69%	-3.89%
12	0.02	0.04	0.05	Unequal	1-0,1-3	35	-7.20%	3.58%	2.11%	-3.88%	-3.71%	-4.35%	-3.71%	-4.35%	-3.88%
12	0.02	0.04	0.05	Unequal	1-0,1-3	50	-7.23%	4.20%	0.42%	-3.95%	-3.69%	-4.32%	-3.69%	-4.32%	-3.95%
12	0.02	0.04	0.05	Unequal	1-3	20	-7.43%	3.75%	-1.24%	-4.24%	-4.24%	-4.24%	-4.24%	-4.24%	-4.24%
12	0.02	0.04	0.05	Unequal	1-3	35	-7.51%	2.16%	0.19%	-4.30%	-4.30%	-4.30%	-4.30%	-4.30%	-4.30%
12	0.02	0.04	0.05	Unequal	1-3	50	-7.46%	2.64%	1.80%	-4.18%	-4.15%	-4.10%	-4.15%	-4.10%	-4.15%
24	0.01	0	0.01	SRS	1-0	20	-0.07%	15.09%	14.78%	-0.07%	-0.07%	-0.07%	-0.07%	-0.07%	-0.07%
24	0.01	0	0.01	SRS	1-0	35	0.08%	17.52%	16.17%	0.08%	0.08%	0.08%	0.08%	0.08%	0.08%
24	0.01	0	0.01	SRS	1-0	50	0.59%	17.26%	15.26%	0.59%	0.59%	0.59%	0.59%	0.59%	0.59%
24	0.01	0	0.01	SRS	1-0,1-3	20	0.03%	16.42%	8.92%	0.03%	0.03%	0.14%	0.03%	0.14%	0.03%
24	0.01	0	0.01	SRS	1-0,1-3	35	0.08%	16.33%	13.83%	0.07%	0.08%	0.00%	0.08%	0.00%	0.07%
24	0.01	0	0.01	SRS	1-0,1-3	50	0.07%	16.25%	14.77%	0.06%	0.07%	0.01%	0.07%	0.01%	0.06%
24	0.01	0	0.01	SRS	1-3	20	-0.26%	15.38%	12.60%	-0.26%	-0.26%	-0.26%	-0.26%	-0.26%	-0.26%
24	0.01	0	0.01	SRS	1-3	35	0.42%	17.71%	12.70%	0.42%	0.42%	0.42%	0.42%	0.42%	0.42%
24	0.01	0	0.01	SRS	1-3	50	0.50%	17.01%	11.80%	0.50%	0.50%	0.50%	0.50%	0.50%	0.50%
24	0.01	0	0.01	StRS	1-0	20	1.07%	16.38%	14.20%	0.75%	0.75%	0.75%	0.75%	0.75%	0.75%
24	0.01	0	0.01	StRS	1-0	35	-0.25%	13.66%	14.49%	-1.01%	-1.01%	-1.01%	-1.01%	-1.01%	-1.01%
24	0.01	0	0.01	StRS	1-0	50	0.58%	17.13%	15.53%	0.31%	0.31%	0.31%	0.31%	0.31%	0.31%
24	0.01	0	0.01	StRS	1-0,1-3	20	-0.64%	16.13%	12.81%	-0.61%	-0.60%	-0.65%	-0.60%	-0.65%	-0.61%
24	0.01	0	0.01	StRS	1-0,1-3	35	-0.95%	15.71%	13.60%	-1.12%	-1.12%	-0.98%	-1.12%	-0.98%	-1.12%
24	0.01	0	0.01	StRS	1-0,1-3	50	-0.29%	15.89%	12.10%	-0.17%	-0.16%	-0.17%	-0.16%	-0.17%	-0.17%
24	0.01	0	0.01	StRS	1-3	20	0.96%	16.90%	7.73%	1.40%	1.40%	1.40%	1.40%	1.40%	1.40%
24	0.01	0	0.01	StRS	1-3	35	1.14%	18.43%	9.60%	1.65%	1.65%	1.65%	1.65%	1.65%	1.65%
24	0.01	0	0.01	StRS	1-3	50	0.26%	16.18%	13.08%	0.10%	0.10%	0.10%	0.10%	0.10%	0.10%

Table B2: Relative Bias of the modified ANC Trend Slope Estimate (shaded cells indicate > 5% or < -5% relative bias)

Years	Trend (p)	Subpop Trend	True Slope	Sampling Design	Revisit Design	Sites	PO	SLRDB	WLRDB	PWIGLS A-only	PWIGLS A	PWIGLS AI	PWIGLS B	PWIGLS BI	PWIGLS C
24	0.01	0	0.01	Unequal	1-0	20	-1.81%	14.56%	13.41%	-0.65%	-0.65%	-0.65%	-0.65%	-0.65%	-0.65%
24	0.01	0	0.01	Unequal	1-0	35	-0.72%	15.52%	16.59%	-0.82%	-0.82%	-0.82%	-0.82%	-0.82%	-0.82%
24	0.01	0	0.01	Unequal	1-0	50	-0.60%	16.27%	14.90%	0.23%	0.23%	0.23%	0.23%	0.23%	0.23%
24	0.01	0	0.01	Unequal	1-0,1-3	20	0.49%	15.97%	9.07%	0.25%	0.25%	0.08%	0.25%	0.08%	0.25%
24	0.01	0	0.01	Unequal	1-0,1-3	35	0.09%	15.67%	10.74%	-0.25%	-0.26%	-0.24%	-0.26%	-0.24%	-0.25%
24	0.01	0	0.01	Unequal	1-0,1-3	50	0.73%	17.18%	12.05%	0.39%	0.37%	0.47%	0.37%	0.47%	0.39%
24	0.01	0	0.01	Unequal	1-3	20	0.40%	16.09%	7.32%	0.74%	0.74%	0.74%	0.74%	0.74%	0.74%
24	0.01	0	0.01	Unequal	1-3	35	0.66%	14.76%	10.04%	0.73%	0.73%	0.73%	0.73%	0.73%	0.73%
24	0.01	0	0.01	Unequal	1-3	50	-0.24%	15.63%	11.35%	-0.56%	-0.56%	-0.56%	-0.56%	-0.56%	-0.56%
24	0.01	0.04	0.04	SRS	1-0	20	-1.06%	5.94%	6.10%	-1.06%	-1.06%	-1.06%	-1.06%	-1.06%	-1.06%
24	0.01	0.04	0.04	SRS	1-0	35	-0.33%	6.89%	6.19%	-0.33%	-0.33%	-0.33%	-0.33%	-0.33%	-0.33%
24	0.01	0.04	0.04	SRS	1-0	50	-0.29%	6.99%	5.72%	-0.29%	-0.29%	-0.29%	-0.29%	-0.29%	-0.29%
24	0.01	0.04	0.04	SRS	1-0,1-3	20	-0.13%	6.62%	4.54%	-0.13%	-0.13%	-0.02%	-0.13%	-0.02%	-0.13%
24	0.01	0.04	0.04	SRS	1-0,1-3	35	-0.04%	7.04%	5.51%	-0.04%	-0.04%	-0.02%	-0.04%	-0.02%	-0.04%
24	0.01	0.04	0.04	SRS	1-0,1-3	50	0.12%	6.98%	5.72%	0.12%	0.12%	0.17%	0.12%	0.17%	0.12%
24	0.01	0.04	0.04	SRS	1-3	20	0.00%	7.13%	4.84%	0.00%	0.00%	0.00%	0.00%	0.00%	0.00%
24	0.01	0.04	0.04	SRS	1-3	35	0.11%	7.32%	5.17%	0.11%	0.11%	0.11%	0.11%	0.11%	0.11%
24	0.01	0.04	0.04	SRS	1-3	50	0.12%	7.04%	5.75%	0.12%	0.12%	0.12%	0.12%	0.12%	0.12%
24	0.01	0.04	0.04	StRS	1-0	20	-0.08%	9.38%	8.75%	-0.08%	-0.08%	-0.08%	-0.08%	-0.08%	-0.08%
24	0.01	0.04	0.04	StRS	1-0	35	-0.20%	9.78%	8.79%	-0.20%	-0.20%	-0.20%	-0.20%	-0.20%	-0.20%
24	0.01	0.04	0.04	StRS	1-0	50	-0.11%	10.02%	8.80%	-0.11%	-0.11%	-0.11%	-0.11%	-0.11%	-0.11%
24	0.01	0.04	0.04	StRS	1-0,1-3	20	0.13%	10.36%	8.07%	0.13%	0.13%	0.12%	0.13%	0.12%	0.13%
24	0.01	0.04	0.04	StRS	1-0,1-3	35	0.03%	9.78%	8.29%	0.03%	0.03%	0.03%	0.03%	0.03%	0.03%
24	0.01	0.04	0.04	StRS	1-0,1-3	50	0.05%	10.01%	8.59%	0.05%	0.05%	0.05%	0.05%	0.05%	0.05%
24	0.01	0.04	0.04	StRS	1-3	20	0.06%	9.28%	6.98%	0.06%	0.06%	0.06%	0.06%	0.06%	0.06%
24	0.01	0.04	0.04	StRS	1-3	35	-0.12%	9.73%	7.39%	-0.12%	-0.12%	-0.12%	-0.12%	-0.12%	-0.12%
24	0.01	0.04	0.04	StRS	1-3	50	0.12%	10.04%	7.99%	0.12%	0.12%	0.12%	0.12%	0.12%	0.12%
24	0.01	0.04	0.04	Unequal	1-0	20	-9.03%	5.26%	4.68%	-1.67%	-1.67%	-1.67%	-1.67%	-1.67%	-1.67%
24	0.01	0.04	0.04	Unequal	1-0	35	-8.69%	7.37%	5.15%	-0.26%	-0.26%	-0.26%	-0.26%	-0.26%	-0.26%
24	0.01	0.04	0.04	Unequal	1-0	50	-9.17%	6.65%	5.35%	-0.39%	-0.39%	-0.39%	-0.39%	-0.39%	-0.39%
24	0.01	0.04	0.04	Unequal	1-0,1-3	20	-9.53%	5.41%	2.73%	-2.10%	-2.08%	-2.58%	-2.08%	-2.58%	-2.10%
24	0.01	0.04	0.04	Unequal	1-0,1-3	35	-8.83%	7.05%	4.69%	-1.05%	-1.01%	-1.28%	-1.01%	-1.28%	-1.05%
24	0.01	0.04	0.04	Unequal	1-0,1-3	50	-9.21%	6.66%	5.04%	-1.68%	-1.64%	-1.87%	-1.64%	-1.87%	-1.68%
24	0.01	0.04	0.04	Unequal	1-3	20	-8.73%	6.85%	1.84%	-1.28%	-1.28%	-1.28%	-1.28%	-1.28%	-1.28%
24	0.01	0.04	0.04	Unequal	1-3	35	-9.00%	6.32%	3.34%	-1.66%	-1.66%	-1.66%	-1.66%	-1.66%	-1.66%
24	0.01	0.04	0.04	Unequal	1-3	50	-9.05%	7.02%	4.16%	-1.49%	-1.49%	-1.49%	-1.49%	-1.49%	-1.49%

Table B3: Confidence Interval Coverage of the ANC Trend Slope Estimate ($\alpha = 0.10$)

Years	Trend (p)	Subpop Trend	Sampling Design	Revisit Design	Effort	Sites	PO	SLRDB	WLRDB	PWIGLS A-only	PWIGLS A	PWIGLS AI	PWIGLS B	PWIGLS BI	PWIGLS C
12	0.02	0	SRS	1-0	1	20	0.88	0.87	0.90	0.33	0.33	0.33	0.33	0.33	0.33
12	0.02	0	SRS	1-0	1	35	0.89	0.88	0.89	0.28	0.28	0.28	0.28	0.28	0.28
12	0.02	0	SRS	1-0	1	50	0.87	0.87	0.88	0.26	0.26	0.26	0.26	0.26	0.26
12	0.02	0	SRS	1-0,1-3	1	20	0.88	0.90	0.89	0.71	0.75	0.71	0.75	0.71	0.71
12	0.02	0	SRS	1-0,1-3	1	35	0.88	0.90	0.91	0.59	0.63	0.59	0.63	0.59	0.59
12	0.02	0	SRS	1-0,1-3	1	50	0.88	0.90	0.91	0.48	0.55	0.48	0.55	0.48	0.48
12	0.02	0	SRS	1-3	1	20	0.93	0.95	0.89	0.73	0.73	0.73	0.73	0.73	0.73
12	0.02	0	SRS	1-3	1	35	0.91	0.93	0.87	0.63	0.63	0.63	0.63	0.63	0.63
12	0.02	0	SRS	1-3	1	50	0.91	0.92	0.89	0.52	0.53	0.52	0.53	0.52	0.53
12	0.02	0	StRS	1-0	1	20	0.90	0.89	0.87	0.38	0.38	0.38	0.38	0.38	0.38
12	0.02	0	StRS	1-0	1	35	0.91	0.92	0.87	0.27	0.27	0.27	0.27	0.27	0.27
12	0.02	0	StRS	1-0	1	50	0.92	0.92	0.87	0.20	0.20	0.20	0.20	0.20	0.20
12	0.02	0	StRS	1-0,1-3	1	20	0.90	0.93	0.88	0.85	0.73	0.86	0.73	0.86	0.85
12	0.02	0	StRS	1-0,1-3	1	35	0.91	0.93	0.91	0.66	0.51	0.66	0.51	0.66	0.66
12	0.02	0	StRS	1-0,1-3	1	50	0.92	0.94	0.90	0.56	0.42	0.54	0.42	0.54	0.56
12	0.02	0	StRS	1-3	1	20	0.88	0.95	0.91	0.66	0.65	0.65	0.65	0.65	0.65
12	0.02	0	StRS	1-3	1	35	0.89	0.94	0.90	0.50	0.50	0.49	0.50	0.49	0.50
12	0.02	0	StRS	1-3	1	50	0.88	0.94	0.90	0.42	0.43	0.40	0.43	0.40	0.43
12	0.02	0	Unequal	1-0	1	20	0.91	0.89	0.83	0.39	0.39	0.39	0.39	0.39	0.39
12	0.02	0	Unequal	1-0	1	35	0.91	0.89	0.83	0.34	0.34	0.34	0.34	0.34	0.34
12	0.02	0	Unequal	1-0	1	50	0.91	0.90	0.84	0.33	0.33	0.33	0.33	0.33	0.33
12	0.02	0	Unequal	1-0,1-3	1	20	0.91	0.93	0.87	0.83	0.82	0.83	0.82	0.83	0.83
12	0.02	0	Unequal	1-0,1-3	1	35	0.89	0.93	0.88	0.76	0.72	0.77	0.72	0.77	0.76
12	0.02	0	Unequal	1-0,1-3	1	50	0.89	0.93	0.90	0.69	0.68	0.67	0.68	0.67	0.70
12	0.02	0	Unequal	1-3	1	20	0.89	0.97	0.90	0.82	0.82	0.82	0.82	0.82	0.82
12	0.02	0	Unequal	1-3	1	35	0.88	0.95	0.90	0.72	0.73	0.72	0.73	0.72	0.73
12	0.02	0	Unequal	1-3	1	50	0.89	0.94	0.89	0.68	0.70	0.66	0.70	0.66	0.70
12	0.02	0.04	SRS	1-0	1	20	0.88	0.85	0.87	0.45	0.45	0.45	0.45	0.45	0.45
12	0.02	0.04	SRS	1-0	1	35	0.89	0.87	0.88	0.36	0.36	0.36	0.36	0.36	0.36
12	0.02	0.04	SRS	1-0	1	50	0.89	0.88	0.88	0.32	0.32	0.32	0.32	0.32	0.32
12	0.02	0.04	SRS	1-0,1-3	1	20	0.90	0.93	0.88	0.78	0.78	0.78	0.78	0.78	0.78
12	0.02	0.04	SRS	1-0,1-3	1	35	0.90	0.92	0.87	0.59	0.67	0.60	0.67	0.60	0.59
12	0.02	0.04	SRS	1-0,1-3	1	50	0.91	0.94	0.89	0.55	0.58	0.56	0.58	0.56	0.55
12	0.02	0.04	SRS	1-3	1	20	0.92	0.96	0.89	0.75	0.75	0.75	0.75	0.75	0.75
12	0.02	0.04	SRS	1-3	1	35	0.92	0.93	0.90	0.63	0.63	0.63	0.63	0.63	0.63
12	0.02	0.04	SRS	1-3	1	50	0.91	0.93	0.87	0.55	0.56	0.55	0.56	0.55	0.56

Table B3: Confidence Interval Coverage of the ANC Trend Slope Estimate (α = 0.10)

Years	Trend (p)	Subpop Trend	Sampling Design	Revisit Design	Effort	Sites	PO	SLRDB	WLRDB	PWIGLS A-only	PWIGLS A	PWIGLS AI	PWIGLS B	PWIGLS BI	PWIGLS C
12	0.02	0.04	StRS	1-0	1	20	0.80	0.86	0.87	0.39	0.39	0.39	0.39	0.39	0.39
12	0.02	0.04	StRS	1-0	1	35	0.77	0.86	0.87	0.30	0.30	0.30	0.30	0.30	0.30
12	0.02	0.04	StRS	1-0	1	50	0.78	0.86	0.87	0.26	0.26	0.26	0.26	0.26	0.26
12	0.02	0.04	StRS	1-0,1-3	1	20	0.82	0.94	0.89	0.83	0.82	0.83	0.82	0.83	0.83
12	0.02	0.04	StRS	1-0,1-3	1	35	0.79	0.93	0.89	0.66	0.68	0.66	0.68	0.66	0.67
12	0.02	0.04	StRS	1-0,1-3	1	50	0.78	0.92	0.89	0.53	0.58	0.53	0.58	0.53	0.53
12	0.02	0.04	StRS	1-3	1	20	0.82	0.95	0.90	0.78	0.78	0.77	0.78	0.77	0.78
12	0.02	0.04	StRS	1-3	1	35	0.80	0.94	0.88	0.70	0.70	0.69	0.70	0.69	0.70
12	0.02	0.04	StRS	1-3	1	50	0.80	0.92	0.89	0.61	0.61	0.61	0.61	0.61	0.61
12	0.02	0.04	Unequal	1-0	1	20	0.89	0.87	0.85	0.48	0.48	0.48	0.48	0.48	0.48
12	0.02	0.04	Unequal	1-0	1	35	0.89	0.88	0.87	0.39	0.39	0.39	0.39	0.39	0.39
12	0.02	0.04	Unequal	1-0	1	50	0.89	0.89	0.87	0.36	0.36	0.36	0.36	0.36	0.36
12	0.02	0.04	Unequal	1-0,1-3	1	20	0.91	0.94	0.88	0.84	0.80	0.84	0.80	0.84	0.84
12	0.02	0.04	Unequal	1-0,1-3	1	35	0.89	0.93	0.89	0.79	0.72	0.79	0.72	0.79	0.79
12	0.02	0.04	Unequal	1-0,1-3	1	50	0.90	0.94	0.89	0.71	0.66	0.67	0.66	0.67	0.71
12	0.02	0.04	Unequal	1-3	1	20	0.87	0.94	0.89	0.75	0.75	0.75	0.75	0.75	0.75
12	0.02	0.04	Unequal	1-3	1	35	0.89	0.95	0.89	0.64	0.65	0.64	0.65	0.64	0.65
12	0.02	0.04	Unequal	1-3	1	50	0.88	0.93	0.92	0.59	0.59	0.57	0.59	0.57	0.59
24	0.01	0	SRS	1-0	1	20	0.90	0.78	0.80	0.59	0.59	0.59	0.59	0.59	0.59
24	0.01	0	SRS	1-0	1	35	0.89	0.78	0.84	0.51	0.51	0.51	0.51	0.51	0.51
24	0.01	0	SRS	1-0	1	50	0.87	0.80	0.84	0.41	0.41	0.41	0.41	0.41	0.41
24	0.01	0	SRS	1-0,1-3	1	20	0.89	0.88	0.86	0.65	0.67	0.64	0.67	0.64	0.65
24	0.01	0	SRS	1-0,1-3	1	35	0.89	0.90	0.89	0.53	0.53	0.52	0.53	0.52	0.53
24	0.01	0	SRS	1-0,1-3	1	50	0.88	0.87	0.87	0.43	0.45	0.43	0.45	0.43	0.43
24	0.01	0	SRS	1-3	1	20	0.87	0.92	0.87	0.60	0.60	0.59	0.60	0.59	0.60
24	0.01	0	SRS	1-3	1	35	0.85	0.87	0.86	0.49	0.49	0.49	0.49	0.49	0.49
24	0.01	0	SRS	1-3	1	50	0.87	0.88	0.88	0.40	0.40	0.40	0.40	0.40	0.40
24	0.01	0	StRS	1-0	1	20	0.89	0.75	0.76	0.66	0.66	0.66	0.66	0.66	0.66
24	0.01	0	StRS	1-0	1	35	0.88	0.78	0.80	0.57	0.57	0.57	0.57	0.57	0.57
24	0.01	0	StRS	1-0	1	50	0.92	0.81	0.80	0.49	0.49	0.49	0.49	0.49	0.49
24	0.01	0	StRS	1-0,1-3	1	20	0.91	0.89	0.84	0.78	0.75	0.81	0.75	0.81	0.78
24	0.01	0	StRS	1-0,1-3	1	35	0.90	0.89	0.87	0.62	0.55	0.63	0.55	0.63	0.62
24	0.01	0	StRS	1-0,1-3	1	50	0.90	0.88	0.85	0.51	0.44	0.51	0.44	0.51	0.51
24	0.01	0	StRS	1-3	1	20	0.92	0.96	0.89	0.68	0.68	0.68	0.68	0.68	0.68
24	0.01	0	StRS	1-3	1	35	0.92	0.94	0.86	0.54	0.54	0.53	0.54	0.53	0.54
24	0.01	0	StRS	1-3	1	50	0.92	0.92	0.88	0.43	0.44	0.42	0.44	0.42	0.44

Table B3: Confidence Interval Coverage of the ANC Trend Slope Estimate (α = 0.10)

Years	Trend (p)	Subpop Trend	Sampling Design	Revisit Design	Effort	Sites	PO	SLRDB	WLRDB	PWIGLS A-only	PWIGLS A	PWIGLS AI	PWIGLS B	PWIGLS BI	PWIGLS C
24	0.01	0	Unequal	1-0	1	20	0.91	0.76	0.75	0.57	0.57	0.57	0.57	0.57	0.57
24	0.01	0	Unequal	1-0	1	35	0.91	0.80	0.79	0.53	0.53	0.53	0.53	0.53	0.53
24	0.01	0	Unequal	1-0	1	50	0.91	0.82	0.79	0.53	0.53	0.53	0.53	0.53	0.53
24	0.01	0	Unequal	1-0,1-3	1	20	0.88	0.90	0.85	0.73	0.70	0.76	0.70	0.76	0.73
24	0.01	0	Unequal	1-0,1-3	1	35	0.91	0.93	0.86	0.67	0.65	0.68	0.65	0.68	0.67
24	0.01	0	Unequal	1-0,1-3	1	50	0.91	0.89	0.86	0.62	0.59	0.62	0.59	0.62	0.62
24	0.01	0	Unequal	1-3	1	20	0.90	0.95	0.88	0.74	0.74	0.74	0.74	0.74	0.74
24	0.01	0	Unequal	1-3	1	35	0.89	0.94	0.86	0.65	0.65	0.65	0.65	0.65	0.65
24	0.01	0	Unequal	1-3	1	50	0.90	0.93	0.88	0.60	0.61	0.59	0.61	0.59	0.61
24	0.01	0.04	SRS	1-0	1	20	0.93	0.69	0.75	0.75	0.75	0.75	0.75	0.75	0.75
24	0.01	0.04	SRS	1-0	1	35	0.90	0.74	0.76	0.69	0.69	0.69	0.69	0.69	0.69
24	0.01	0.04	SRS	1-0	1	50	0.92	0.75	0.77	0.66	0.66	0.66	0.66	0.66	0.66
24	0.01	0.04	SRS	1-0,1-3	1	20	0.90	0.81	0.84	0.76	0.79	0.76	0.79	0.76	0.76
24	0.01	0.04	SRS	1-0,1-3	1	35	0.90	0.85	0.82	0.61	0.64	0.62	0.64	0.62	0.61
24	0.01	0.04	SRS	1-0,1-3	1	50	0.90	0.82	0.84	0.52	0.54	0.53	0.54	0.53	0.52
24	0.01	0.04	SRS	1-3	1	20	0.91	0.88	0.88	0.67	0.67	0.67	0.67	0.67	0.67
24	0.01	0.04	SRS	1-3	1	35	0.91	0.86	0.87	0.56	0.56	0.56	0.56	0.56	0.56
24	0.01	0.04	SRS	1-3	1	50	0.92	0.84	0.88	0.50	0.50	0.50	0.50	0.50	0.50
24	0.01	0.04	StRS	1-0	1	20	0.85	0.64	0.71	0.72	0.72	0.72	0.72	0.72	0.72
24	0.01	0.04	StRS	1-0	1	35	0.86	0.67	0.72	0.66	0.66	0.66	0.66	0.66	0.66
24	0.01	0.04	StRS	1-0	1	50	0.86	0.66	0.75	0.57	0.57	0.57	0.57	0.57	0.57
24	0.01	0.04	StRS	1-0,1-3	1	20	0.85	0.78	0.82	0.80	0.81	0.80	0.81	0.80	0.80
24	0.01	0.04	StRS	1-0,1-3	1	35	0.81	0.76	0.84	0.63	0.63	0.62	0.63	0.62	0.63
24	0.01	0.04	StRS	1-0,1-3	1	50	0.83	0.76	0.81	0.57	0.57	0.56	0.57	0.56	0.57
24	0.01	0.04	StRS	1-3	1	20	0.82	0.88	0.83	0.73	0.73	0.73	0.73	0.73	0.73
24	0.01	0.04	StRS	1-3	1	35	0.81	0.83	0.83	0.61	0.61	0.61	0.61	0.61	0.61
24	0.01	0.04	StRS	1-3	1	50	0.82	0.81	0.82	0.54	0.55	0.54	0.55	0.54	0.55
24	0.01	0.04	Unequal	1-0	1	20	0.86	0.63	0.71	0.72	0.72	0.72	0.72	0.72	0.72
24	0.01	0.04	Unequal	1-0	1	35	0.81	0.74	0.73	0.70	0.70	0.70	0.70	0.70	0.70
24	0.01	0.04	Unequal	1-0	1	50	0.79	0.71	0.75	0.60	0.60	0.60	0.60	0.60	0.60
24	0.01	0.04	Unequal	1-0,1-3	1	20	0.78	0.85	0.78	0.81	0.76	0.80	0.76	0.80	0.81
24	0.01	0.04	Unequal	1-0,1-3	1	35	0.74	0.88	0.82	0.70	0.65	0.69	0.65	0.69	0.70
24	0.01	0.04	Unequal	1-0,1-3	1	50	0.73	0.87	0.82	0.67	0.59	0.65	0.59	0.65	0.67
24	0.01	0.04	Unequal	1-3	1	20	0.77	0.93	0.88	0.69	0.69	0.69	0.69	0.69	0.69
24	0.01	0.04	Unequal	1-3	1	35	0.76	0.89	0.87	0.58	0.58	0.58	0.58	0.58	0.58
24	0.01	0.04	Unequal	1-3	1	50	0.74	0.89	0.84	0.52	0.52	0.49	0.52	0.49	0.52

Table B4: Confidence Interval Coverage of the modified ANC Trend Slope Estimate (α = 0.10)

Years	Trend (p)	Subpop Trend	Sampling Design	Revisit Design	Effort	Sites	PO	SLRDB	WLRDB	PWIGLS A-only	PWIGLS A	PWIGLS AI	PWIGLS B	PWIGLS BI	PWIGLS C
12	0.02	0	SRS	1-0	1	20	0.91	0.74	0.70	0.90	0.90	0.90	0.90	0.90	0.90
12	0.02	0	SRS	1-0	1	35	0.92	0.76	0.75	0.89	0.89	0.89	0.89	0.89	0.89
12	0.02	0	SRS	1-0	1	50	0.90	0.71	0.75	0.87	0.87	0.87	0.87	0.87	0.87
12	0.02	0	SRS	1-0,1-3	1	20	0.91	0.97	0.97	1.00	1.00	1.00	1.00	1.00	1.00
12	0.02	0	SRS	1-0,1-3	1	35	0.89	0.99	0.97	1.00	1.00	1.00	1.00	1.00	1.00
12	0.02	0	SRS	1-0,1-3	1	50	0.91	0.99	0.98	1.00	1.00	1.00	1.00	1.00	1.00
12	0.02	0	SRS	1-3	1	20	0.90	1.00	0.99	1.00	1.00	1.00	1.00	1.00	1.00
12	0.02	0	SRS	1-3	1	35	0.90	0.99	0.99	1.00	1.00	1.00	1.00	1.00	1.00
12	0.02	0	SRS	1-3	1	50	0.91	0.99	0.97	1.00	1.00	1.00	1.00	1.00	1.00
12	0.02	0	StRS	1-0	1	20	0.90	0.74	0.73	0.90	0.90	0.90	0.90	0.90	0.90
12	0.02	0	StRS	1-0	1	35	0.92	0.74	0.70	0.90	0.90	0.90	0.90	0.90	0.90
12	0.02	0	StRS	1-0	1	50	0.91	0.72	0.75	0.90	0.90	0.90	0.90	0.90	0.90
12	0.02	0	StRS	1-0,1-3	1	20	0.92	0.99	0.97	1.00	1.00	1.00	1.00	1.00	1.00
12	0.02	0	StRS	1-0,1-3	1	35	0.93	0.99	0.98	1.00	1.00	1.00	1.00	1.00	1.00
12	0.02	0	StRS	1-0,1-3	1	50	0.92	0.99	0.98	1.00	1.00	1.00	1.00	1.00	1.00
12	0.02	0	StRS	1-3	1	20	0.92	0.99	0.98	1.00	1.00	1.00	1.00	1.00	1.00
12	0.02	0	StRS	1-3	1	35	0.93	1.00	0.99	1.00	1.00	1.00	1.00	1.00	1.00
12	0.02	0	StRS	1-3	1	50	0.93	0.99	0.98	1.00	1.00	1.00	1.00	1.00	1.00
12	0.02	0	Unequal	1-0	1	20	0.91	0.73	0.75	0.87	0.87	0.87	0.87	0.87	0.87
12	0.02	0	Unequal	1-0	1	35	0.91	0.77	0.78	0.91	0.91	0.91	0.91	0.91	0.91
12	0.02	0	Unequal	1-0	1	50	0.92	0.75	0.72	0.94	0.94	0.94	0.94	0.94	0.94
12	0.02	0	Unequal	1-0,1-3	1	20	0.93	0.97	0.94	1.00	1.00	1.00	1.00	1.00	1.00
12	0.02	0	Unequal	1-0,1-3	1	35	0.91	0.98	0.98	1.00	1.00	1.00	1.00	1.00	1.00
12	0.02	0	Unequal	1-0,1-3	1	50	0.90	0.99	0.98	1.00	1.00	1.00	1.00	1.00	1.00
12	0.02	0	Unequal	1-3	1	20	0.90	0.99	0.98	1.00	1.00	1.00	1.00	1.00	1.00
12	0.02	0	Unequal	1-3	1	35	0.91	1.00	0.99	1.00	1.00	1.00	1.00	1.00	1.00
12	0.02	0	Unequal	1-3	1	50	0.92	0.99	0.99	1.00	1.00	1.00	1.00	1.00	1.00
12	0.02	0.04	SRS	1-0	1	20	0.92	0.64	0.63	0.90	0.90	0.90	0.90	0.90	0.90
12	0.02	0.04	SRS	1-0	1	35	0.92	0.59	0.60	0.91	0.91	0.91	0.91	0.91	0.91
12	0.02	0.04	SRS	1-0	1	50	0.95	0.60	0.64	0.94	0.94	0.94	0.94	0.94	0.94
12	0.02	0.04	SRS	1-0,1-3	1	20	0.94	0.94	0.96	1.00	1.00	1.00	1.00	1.00	1.00
12	0.02	0.04	SRS	1-0,1-3	1	35	0.90	0.95	0.96	1.00	1.00	1.00	1.00	1.00	1.00
12	0.02	0.04	SRS	1-0,1-3	1	50	0.93	0.97	0.97	1.00	1.00	1.00	1.00	1.00	1.00
12	0.02	0.04	SRS	1-3	1	20	0.95	0.99	0.98	1.00	1.00	1.00	1.00	1.00	1.00
12	0.02	0.04	SRS	1-3	1	35	0.92	0.98	0.98	1.00	1.00	1.00	1.00	1.00	1.00
12	0.02	0.04	SRS	1-3	1	50	0.92	0.98	0.97	1.00	1.00	1.00	1.00	1.00	1.00

Table B4: Confidence Interval Coverage of the modified ANC Trend Slope Estimate (α = 0.10)

Years	Trend (p)	Subpop Trend	Sampling Design	Revisit Design	Effort	Sites	PO	SLRDB	WLRDB	PWIGLS A-only	PWIGLS A	PWIGLS AI	PWIGLS B	PWIGLS BI	PWIGLS C
12	0.02	0.04	StRS	1-0	1	20	0.89	0.63	0.65	0.87	0.87	0.87	0.87	0.87	0.87
12	0.02	0.04	StRS	1-0	1	35	0.90	0.56	0.55	0.89	0.89	0.89	0.89	0.89	0.89
12	0.02	0.04	StRS	1-0	1	50	0.88	0.48	0.52	0.86	0.86	0.86	0.86	0.86	0.86
12	0.02	0.04	StRS	1-0,1-3	1	20	0.89	0.94	0.95	1.00	1.00	1.00	1.00	1.00	1.00
12	0.02	0.04	StRS	1-0,1-3	1	35	0.89	0.96	0.96	1.00	1.00	1.00	1.00	1.00	1.00
12	0.02	0.04	StRS	1-0,1-3	1	50	0.89	0.95	0.95	1.00	1.00	1.00	1.00	1.00	1.00
12	0.02	0.04	StRS	1-3	1	20	0.89	0.98	0.96	1.00	1.00	1.00	1.00	1.00	1.00
12	0.02	0.04	StRS	1-3	1	35	0.86	0.96	0.98	1.00	1.00	1.00	1.00	1.00	1.00
12	0.02	0.04	StRS	1-3	1	50	0.90	0.96	0.95	1.00	1.00	1.00	1.00	1.00	1.00
12	0.02	0.04	Unequal	1-0	1	20	0.87	0.64	0.66	0.89	0.89	0.89	0.89	0.89	0.89
12	0.02	0.04	Unequal	1-0	1	35	0.78	0.63	0.69	0.89	0.89	0.89	0.89	0.89	0.89
12	0.02	0.04	Unequal	1-0	1	50	0.66	0.65	0.66	0.89	0.89	0.89	0.89	0.89	0.89
12	0.02	0.04	Unequal	1-0,1-3	1	20	0.75	0.95	0.94	1.00	1.00	1.00	1.00	1.00	1.00
12	0.02	0.04	Unequal	1-0,1-3	1	35	0.59	0.96	0.96	1.00	1.00	1.00	1.00	1.00	1.00
12	0.02	0.04	Unequal	1-0,1-3	1	50	0.44	0.97	0.97	1.00	1.00	1.00	1.00	1.00	1.00
12	0.02	0.04	Unequal	1-3	1	20	0.69	0.99	0.98	1.00	1.00	1.00	1.00	1.00	1.00
12	0.02	0.04	Unequal	1-3	1	35	0.51	0.99	0.97	1.00	1.00	1.00	1.00	1.00	1.00
12	0.02	0.04	Unequal	1-3	1	50	0.42	0.99	0.97	1.00	1.00	1.00	1.00	1.00	1.00
24	0.01	0	SRS	1-0	1	20	0.92	0.34	0.34	0.92	0.92	0.92	0.92	0.92	0.92
24	0.01	0	SRS	1-0	1	35	0.89	0.32	0.31	0.89	0.89	0.89	0.89	0.89	0.89
24	0.01	0	SRS	1-0	1	50	0.90	0.27	0.31	0.90	0.90	0.90	0.90	0.90	0.90
24	0.01	0	SRS	1-0,1-3	1	20	0.93	0.83	0.85	0.98	0.98	0.98	0.98	0.98	0.98
24	0.01	0	SRS	1-0,1-3	1	35	0.91	0.84	0.88	0.98	0.98	0.98	0.98	0.98	0.98
24	0.01	0	SRS	1-0,1-3	1	50	0.89	0.79	0.83	0.97	0.97	0.98	0.97	0.98	0.97
24	0.01	0	SRS	1-3	1	20	0.91	0.96	0.89	0.99	0.99	0.99	0.99	0.99	0.99
24	0.01	0	SRS	1-3	1	35	0.88	0.89	0.90	0.99	0.99	0.99	0.99	0.99	0.99
24	0.01	0	SRS	1-3	1	50	0.88	0.87	0.91	0.97	0.97	0.97	0.97	0.97	0.97
24	0.01	0	StRS	1-0	1	20	0.89	0.36	0.33	0.89	0.89	0.89	0.89	0.89	0.89
24	0.01	0	StRS	1-0	1	35	0.90	0.35	0.39	0.90	0.90	0.90	0.90	0.90	0.90
24	0.01	0	StRS	1-0	1	50	0.89	0.32	0.30	0.90	0.90	0.90	0.90	0.90	0.90
24	0.01	0	StRS	1-0,1-3	1	20	0.91	0.82	0.79	0.99	0.99	0.98	0.99	0.98	0.99
24	0.01	0	StRS	1-0,1-3	1	35	0.90	0.87	0.84	0.98	0.98	0.98	0.98	0.98	0.98
24	0.01	0	StRS	1-0,1-3	1	50	0.87	0.88	0.88	0.97	0.97	0.98	0.97	0.98	0.97
24	0.01	0	StRS	1-3	1	20	0.89	0.94	0.95	0.99	0.99	0.99	0.99	0.99	0.99
24	0.01	0	StRS	1-3	1	35	0.89	0.91	0.93	0.99	0.99	0.99	0.99	0.99	0.99
24	0.01	0	StRS	1-3	1	50	0.93	0.91	0.90	0.99	0.99	0.99	0.99	0.99	0.99

Table B4: Confidence Interval Coverage of the modified ANC Trend Slope Estimate (α = 0.10)

Years	Trend (p)	Subpop Trend	Sampling Design	Revisit Design	Effort	Sites	PO	SLRDB	WLRDB	PWIGLS A-only	PWIGLS A	PWIGLS AI	PWIGLS B	PWIGLS BI	PWIGLS C
24	0.01	0	Unequal	1-0	1	20	0.90	0.36	0.36	0.86	0.86	0.86	0.86	0.86	0.86
24	0.01	0	Unequal	1-0	1	35	0.90	0.37	0.35	0.89	0.89	0.89	0.89	0.89	0.89
24	0.01	0	Unequal	1-0	1	50	0.89	0.34	0.32	0.87	0.87	0.87	0.87	0.87	0.87
24	0.01	0	Unequal	1-0,1-3	1	20	0.90	0.84	0.79	0.97	0.97	0.98	0.97	0.98	0.97
24	0.01	0	Unequal	1-0,1-3	1	35	0.90	0.89	0.87	1.00	1.00	1.00	1.00	1.00	1.00
24	0.01	0	Unequal	1-0,1-3	1	50	0.92	0.90	0.89	0.99	0.99	0.99	0.99	0.99	0.99
24	0.01	0	Unequal	1-3	1	20	0.89	0.97	0.95	0.99	0.99	0.99	0.99	0.99	0.99
24	0.01	0	Unequal	1-3	1	35	0.89	0.95	0.95	0.99	0.99	0.99	0.99	0.99	0.99
24	0.01	0	Unequal	1-3	1	50	0.91	0.96	0.92	1.00	1.00	1.00	1.00	1.00	1.00
24	0.01	0.04	SRS	1-0	1	20	0.93	0.23	0.27	0.93	0.93	0.93	0.93	0.93	0.93
24	0.01	0.04	SRS	1-0	1	35	0.93	0.16	0.25	0.93	0.93	0.93	0.93	0.93	0.93
24	0.01	0.04	SRS	1-0	1	50	0.93	0.13	0.24	0.93	0.93	0.93	0.93	0.93	0.93
24	0.01	0.04	SRS	1-0,1-3	1	20	0.93	0.64	0.74	0.97	0.97	0.97	0.97	0.97	0.97
24	0.01	0.04	SRS	1-0,1-3	1	35	0.94	0.64	0.73	0.98	0.98	0.98	0.98	0.98	0.98
24	0.01	0.04	SRS	1-0,1-3	1	50	0.96	0.59	0.69	0.99	0.99	0.99	0.99	0.99	0.99
24	0.01	0.04	SRS	1-3	1	20	0.95	0.79	0.88	0.98	0.98	0.98	0.98	0.98	0.98
24	0.01	0.04	SRS	1-3	1	35	0.95	0.71	0.81	0.98	0.98	0.98	0.98	0.98	0.98
24	0.01	0.04	SRS	1-3	1	50	0.95	0.63	0.74	0.98	0.98	0.98	0.98	0.98	0.98
24	0.01	0.04	StRS	1-0	1	20	0.90	0.21	0.23	0.89	0.89	0.89	0.89	0.89	0.89
24	0.01	0.04	StRS	1-0	1	35	0.89	0.11	0.14	0.87	0.87	0.87	0.87	0.87	0.87
24	0.01	0.04	StRS	1-0	1	50	0.90	0.06	0.10	0.90	0.90	0.90	0.90	0.90	0.90
24	0.01	0.04	StRS	1-0,1-3	1	20	0.90	0.59	0.65	0.96	0.96	0.96	0.96	0.96	0.96
24	0.01	0.04	StRS	1-0,1-3	1	35	0.91	0.54	0.65	0.98	0.98	0.98	0.98	0.98	0.98
24	0.01	0.04	StRS	1-0,1-3	1	50	0.87	0.49	0.58	0.97	0.97	0.97	0.97	0.97	0.97
24	0.01	0.04	StRS	1-3	1	20	0.91	0.80	0.83	0.98	0.98	0.98	0.98	0.98	0.98
24	0.01	0.04	StRS	1-3	1	35	0.88	0.64	0.74	0.98	0.98	0.98	0.98	0.98	0.98
24	0.01	0.04	StRS	1-3	1	50	0.87	0.51	0.70	0.98	0.98	0.98	0.98	0.98	0.98
24	0.01	0.04	Unequal	1-0	1	20	0.83	0.27	0.33	0.87	0.87	0.87	0.87	0.87	0.87
24	0.01	0.04	Unequal	1-0	1	35	0.76	0.20	0.32	0.89	0.89	0.89	0.89	0.89	0.89
24	0.01	0.04	Unequal	1-0	1	50	0.60	0.28	0.30	0.93	0.93	0.93	0.93	0.93	0.93
24	0.01	0.04	Unequal	1-0,1-3	1	20	0.58	0.75	0.73	0.96	0.96	0.93	0.96	0.93	0.96
24	0.01	0.04	Unequal	1-0,1-3	1	35	0.32	0.79	0.80	0.98	0.98	0.97	0.98	0.97	0.98
24	0.01	0.04	Unequal	1-0,1-3	1	50	0.09	0.79	0.80	0.99	0.99	0.97	0.99	0.97	0.99
24	0.01	0.04	Unequal	1-3	1	20	0.50	0.92	0.93	0.98	0.98	0.98	0.98	0.98	0.98
24	0.01	0.04	Unequal	1-3	1	35	0.19	0.87	0.91	0.97	0.97	0.97	0.97	0.97	0.97
24	0.01	0.04	Unequal	1-3	1	50	0.06	0.83	0.88	0.97	0.97	0.97	0.97	0.97	0.97

Table B5: Trend Test Size of the *t*-test for the ANC Trend Slope Estimate (shaded cells indicate > 0.13)

Years	Trend (p)	Subpop Trend	Sampling Design	Revisit Design	Sites	PO	SLRDB	WLRDB	PWIGLS A-only	PWIGLS A	PWIGLS AI	PWIGLS B	PWIGLS BI	PWIGLS C
12	0	0	SRS	1-0	20	0.11	0.12	0.11	0.65	0.65	0.65	0.65	0.65	0.65
12	0	0	SRS	1-0	35	0.10	0.11	0.12	0.71	0.71	0.71	0.71	0.71	0.71
12	0	0	SRS	1-0	50	0.10	0.12	0.14	0.76	0.76	0.76	0.76	0.76	0.76
12	0	0	SRS	1-0,1-3	20	0.11	0.10	0.11	0.27	0.25	0.28	0.25	0.28	0.27
12	0	0	SRS	1-0,1-3	35	0.10	0.08	0.10	0.43	0.40	0.43	0.40	0.43	0.43
12	0	0	SRS	1-0,1-3	50	0.11	0.09	0.11	0.48	0.48	0.50	0.48	0.50	0.48
12	0	0	SRS	1-3	20	0.11	0.05	0.08	0.29	0.29	0.29	0.29	0.29	0.29
12	0	0	SRS	1-3	35	0.11	0.08	0.11	0.38	0.38	0.39	0.38	0.39	0.38
12	0	0	SRS	1-3	50	0.11	0.08	0.10	0.46	0.46	0.46	0.46	0.46	0.46
12	0	0	StRS	1-0	20	0.12	0.12	0.10	0.61	0.61	0.61	0.61	0.61	0.61
12	0	0	StRS	1-0	35	0.09	0.10	0.11	0.76	0.76	0.76	0.76	0.76	0.76
12	0	0	StRS	1-0	50	0.10	0.10	0.10	0.81	0.81	0.81	0.81	0.81	0.81
12	0	0	StRS	1-0,1-3	20	0.12	0.09	0.09	0.19	0.30	0.18	0.30	0.18	0.19
12	0	0	StRS	1-0,1-3	35	0.12	0.08	0.09	0.36	0.49	0.36	0.49	0.36	0.36
12	0	0	StRS	1-0,1-3	50	0.13	0.09	0.09	0.49	0.63	0.52	0.63	0.52	0.49
12	0	0	StRS	1-3	20	0.10	0.06	0.11	0.34	0.34	0.35	0.34	0.35	0.34
12	0	0	StRS	1-3	35	0.10	0.06	0.10	0.54	0.54	0.55	0.54	0.55	0.54
12	0	0	StRS	1-3	50	0.11	0.08	0.12	0.62	0.61	0.64	0.61	0.64	0.61
12	0	0	Unequal	1-0	20	0.12	0.12	0.10	0.59	0.59	0.59	0.59	0.59	0.59
12	0	0	Unequal	1-0	35	0.10	0.10	0.08	0.64	0.64	0.64	0.64	0.64	0.64
12	0	0	Unequal	1-0	50	0.10	0.11	0.08	0.66	0.66	0.66	0.66	0.66	0.66
12	0	0	Unequal	1-0,1-3	20	0.12	0.09	0.12	0.21	0.23	0.20	0.23	0.20	0.21
12	0	0	Unequal	1-0,1-3	35	0.11	0.07	0.13	0.27	0.26	0.27	0.26	0.27	0.27
12	0	0	Unequal	1-0,1-3	50	0.10	0.07	0.11	0.31	0.31	0.32	0.31	0.32	0.31
12	0	0	Unequal	1-3	20	0.13	0.03	0.10	0.16	0.16	0.16	0.16	0.16	0.16
12	0	0	Unequal	1-3	35	0.12	0.06	0.12	0.29	0.29	0.30	0.29	0.30	0.29
12	0	0	Unequal	1-3	50	0.12	0.05	0.13	0.34	0.32	0.35	0.32	0.35	0.32
24	0	0	SRS	1-0	20	0.10	0.19	0.20	0.37	0.37	0.37	0.37	0.37	0.37
24	0	0	SRS	1-0	35	0.09	0.16	0.17	0.46	0.46	0.46	0.46	0.46	0.46
24	0	0	SRS	1-0	50	0.10	0.17	0.16	0.54	0.54	0.54	0.54	0.54	0.54
24	0	0	SRS	1-0,1-3	20	0.09	0.10	0.17	0.34	0.33	0.34	0.33	0.34	0.34
24	0	0	SRS	1-0,1-3	35	0.10	0.12	0.12	0.46	0.43	0.46	0.43	0.46	0.46
24	0	0	SRS	1-0,1-3	50	0.09	0.09	0.14	0.55	0.50	0.53	0.50	0.53	0.55
24	0	0	SRS	1-3	20	0.10	0.09	0.14	0.37	0.37	0.37	0.37	0.37	0.37
24	0	0	SRS	1-3	35	0.10	0.10	0.15	0.53	0.53	0.53	0.53	0.53	0.53
24	0	0	SRS	1-3	50	0.10	0.10	0.14	0.58	0.58	0.59	0.58	0.59	0.58

Table B5: Trend Test Size of the *t*-test for the ANC Trend Slope Estimate (shaded cells indicate > 0.13)

Years	Trend (p)	Subpop Trend	Sampling Design	Revisit Design	Sites	PO	SLRDB	WLRDB	PWIGLS A-only	PWIGLS A	PWIGLS AI	PWIGLS B	PWIGLS BI	PWIGLS C
24	0	0	StRS	1-0	20	0.08	0.22	0.26	0.32	0.32	0.32	0.32	0.32	0.32
24	0	0	StRS	1-0	35	0.09	0.18	0.22	0.42	0.42	0.42	0.42	0.42	0.42
24	0	0	StRS	1-0	50	0.11	0.15	0.20	0.50	0.50	0.50	0.50	0.50	0.50
24	0	0	StRS	1-0,1-3	20	0.09	0.11	0.19	0.21	0.26	0.21	0.26	0.21	0.21
24	0	0	StRS	1-0,1-3	35	0.10	0.07	0.13	0.39	0.46	0.38	0.46	0.38	0.39
24	0	0	StRS	1-0,1-3	50	0.11	0.11	0.13	0.50	0.57	0.52	0.57	0.52	0.50
24	0	0	StRS	1-3	20	0.11	0.05	0.12	0.31	0.31	0.32	0.31	0.32	0.31
24	0	0	StRS	1-3	35	0.10	0.07	0.09	0.51	0.51	0.51	0.51	0.51	0.51
24	0	0	StRS	1-3	50	0.10	0.09	0.10	0.57	0.56	0.57	0.56	0.57	0.56
24	0	0	Unequal	1-0	20	0.10	0.23	0.26	0.40	0.40	0.40	0.40	0.40	0.40
24	0	0	Unequal	1-0	35	0.11	0.21	0.21	0.42	0.42	0.42	0.42	0.42	0.42
24	0	0	Unequal	1-0	50	0.12	0.21	0.24	0.50	0.50	0.50	0.50	0.50	0.50
24	0	0	Unequal	1-0,1-3	20	0.08	0.11	0.16	0.24	0.27	0.22	0.27	0.22	0.24
24	0	0	Unequal	1-0,1-3	35	0.09	0.09	0.14	0.30	0.35	0.32	0.35	0.32	0.30
24	0	0	Unequal	1-0,1-3	50	0.08	0.08	0.12	0.33	0.36	0.33	0.36	0.33	0.33
24	0	0	Unequal	1-3	20	0.10	0.05	0.13	0.31	0.31	0.31	0.31	0.31	0.31
24	0	0	Unequal	1-3	35	0.10	0.05	0.13	0.37	0.37	0.38	0.37	0.38	0.37
24	0	0	Unequal	1-3	50	0.11	0.08	0.10	0.45	0.44	0.45	0.44	0.45	0.44

Table B6: Trend Test Size of the *t*-test for the modified ANC Trend Slope Estimate (shaded cells indicate > 0.13 or < 0.07)

Years	Trend (p)	Subpop Trend	Sampling Design	Revisit Design	Sites	PO	SLRDB	WLRDB	PWIGLS A-only	PWIGLS A	PWIGLS AI	PWIGLS B	PWIGLS BI	PWIGLS C
12	0	0	SRS	1-0	20	0.07	0.25	0.30	0.10	0.10	0.10	0.10	0.10	0.10
12	0	0	SRS	1-0	35	0.10	0.30	0.29	0.12	0.12	0.12	0.12	0.12	0.12
12	0	0	SRS	1-0	50	0.09	0.26	0.27	0.11	0.11	0.11	0.11	0.11	0.11
12	0	0	SRS	1-0,1-3	20	0.10	0.03	0.05	0.00	0.00	0.00	0.00	0.00	0.00
12	0	0	SRS	1-0,1-3	35	0.07	0.02	0.03	0.00	0.00	0.00	0.00	0.00	0.00
12	0	0	SRS	1-0,1-3	50	0.08	0.02	0.02	0.00	0.00	0.00	0.00	0.00	0.00
12	0	0	SRS	1-3	20	0.08	0.01	0.01	0.00	0.00	0.00	0.00	0.00	0.00
12	0	0	SRS	1-3	35	0.07	0.01	0.01	0.00	0.00	0.00	0.00	0.00	0.00
12	0	0	SRS	1-3	50	0.10	0.02	0.02	0.00	0.00	0.00	0.00	0.00	0.00
12	0	0	StRS	1-0	20	0.07	0.27	0.27	0.06	0.06	0.06	0.06	0.06	0.06
12	0	0	StRS	1-0	35	0.11	0.28	0.30	0.12	0.12	0.12	0.12	0.12	0.12
12	0	0	StRS	1-0	50	0.09	0.26	0.28	0.09	0.09	0.09	0.09	0.09	0.09
12	0	0	StRS	1-0,1-3	20	0.11	0.02	0.04	0.00	0.00	0.00	0.00	0.00	0.00
12	0	0	StRS	1-0,1-3	35	0.09	0.02	0.01	0.00	0.00	0.00	0.00	0.00	0.00
12	0	0	StRS	1-0,1-3	50	0.06	0.02	0.01	0.00	0.00	0.00	0.00	0.00	0.00
12	0	0	StRS	1-3	20	0.10	0.01	0.01	0.00	0.00	0.00	0.00	0.00	0.00
12	0	0	StRS	1-3	35	0.05	0.01	0.02	0.00	0.00	0.00	0.00	0.00	0.00
12	0	0	StRS	1-3	50	0.08	0.01	0.01	0.00	0.00	0.00	0.00	0.00	0.00
12	0	0	Unequal	1-0	20	0.08	0.27	0.26	0.12	0.12	0.12	0.12	0.12	0.12
12	0	0	Unequal	1-0	35	0.11	0.30	0.30	0.09	0.09	0.09	0.09	0.09	0.09
12	0	0	Unequal	1-0	50	0.07	0.28	0.27	0.04	0.04	0.04	0.04	0.04	0.04
12	0	0	Unequal	1-0,1-3	20	0.08	0.02	0.04	0.00	0.00	0.00	0.00	0.00	0.00
12	0	0	Unequal	1-0,1-3	35	0.10	0.03	0.03	0.00	0.00	0.00	0.00	0.00	0.00
12	0	0	Unequal	1-0,1-3	50	0.09	0.01	0.02	0.00	0.00	0.00	0.00	0.00	0.00
12	0	0	Unequal	1-3	20	0.08	0.00	0.02	0.00	0.00	0.00	0.00	0.00	0.00
12	0	0	Unequal	1-3	35	0.10	0.01	0.01	0.00	0.00	0.00	0.00	0.00	0.00
12	0	0	Unequal	1-3	50	0.09	0.00	0.01	0.00	0.00	0.00	0.00	0.00	0.00
24	0	0	SRS	1-0	20	0.10	0.67	0.64	0.11	0.11	0.11	0.11	0.11	0.11
24	0	0	SRS	1-0	35	0.11	0.66	0.65	0.12	0.12	0.12	0.12	0.12	0.12
24	0	0	SRS	1-0	50	0.09	0.71	0.69	0.10	0.10	0.10	0.10	0.10	0.10
24	0	0	SRS	1-0,1-3	20	0.09	0.19	0.24	0.03	0.03	0.03	0.03	0.03	0.03
24	0	0	SRS	1-0,1-3	35	0.09	0.14	0.14	0.02	0.02	0.02	0.02	0.02	0.02
24	0	0	SRS	1-0,1-3	50	0.10	0.16	0.15	0.02	0.02	0.02	0.02	0.02	0.02
24	0	0	SRS	1-3	20	0.08	0.07	0.07	0.01	0.01	0.01	0.01	0.01	0.01
24	0	0	SRS	1-3	35	0.10	0.11	0.09	0.02	0.02	0.02	0.02	0.02	0.02
24	0	0	SRS	1-3	50	0.09	0.14	0.10	0.02	0.02	0.02	0.02	0.02	0.02

Table B6: Trend Test Size of the *t*-test for the modified ANC Trend Slope Estimate (shaded cells indicate > 0.13 or < 0.07)

Years	Trend (p)	Subpop Trend	Sampling Design	Revisit Design	Sites	PO	SLRDB	WLRDB	PWIGLS A-only	PWIGLS A	PWIGLS AI	PWIGLS B	PWIGLS BI	PWIGLS C
24	0	0	StRS	1-0	20	0.12	0.64	0.65	0.10	0.10	0.10	0.10	0.10	0.10
24	0	0	StRS	1-0	35	0.09	0.64	0.65	0.11	0.11	0.11	0.11	0.11	0.11
24	0	0	StRS	1-0	50	0.11	0.70	0.70	0.14	0.14	0.14	0.14	0.14	0.14
24	0	0	StRS	1-0,1-3	20	0.08	0.17	0.20	0.02	0.02	0.03	0.02	0.03	0.02
24	0	0	StRS	1-0,1-3	35	0.08	0.15	0.15	0.02	0.02	0.02	0.02	0.02	0.02
24	0	0	StRS	1-0,1-3	50	0.11	0.15	0.13	0.01	0.01	0.01	0.01	0.01	0.01
24	0	0	StRS	1-3	20	0.10	0.04	0.07	0.02	0.02	0.02	0.02	0.02	0.02
24	0	0	StRS	1-3	35	0.11	0.08	0.06	0.02	0.02	0.02	0.02	0.02	0.02
24	0	0	StRS	1-3	50	0.08	0.09	0.07	0.02	0.02	0.02	0.02	0.02	0.02
24	0	0	Unequal	1-0	20	0.09	0.63	0.63	0.16	0.16	0.16	0.16	0.16	0.16
24	0	0	Unequal	1-0	35	0.10	0.62	0.64	0.12	0.12	0.12	0.12	0.12	0.12
24	0	0	Unequal	1-0	50	0.12	0.65	0.66	0.11	0.11	0.11	0.11	0.11	0.11
24	0	0	Unequal	1-0,1-3	20	0.07	0.17	0.17	0.02	0.02	0.02	0.02	0.02	0.02
24	0	0	Unequal	1-0,1-3	35	0.09	0.11	0.13	0.01	0.01	0.01	0.01	0.01	0.01
24	0	0	Unequal	1-0,1-3	50	0.09	0.10	0.11	0.01	0.01	0.01	0.01	0.01	0.01
24	0	0	Unequal	1-3	20	0.10	0.04	0.07	0.01	0.01	0.01	0.01	0.01	0.01
24	0	0	Unequal	1-3	35	0.09	0.05	0.05	0.00	0.00	0.00	0.00	0.00	0.00
24	0	0	Unequal	1-3	50	0.11	0.07	0.05	0.00	0.00	0.00	0.00	0.00	0.00

Table B7: Trend Test Power of the *t*-test for the ANC Trend Slope Estimate (shaded cells indicate power below 0.8, results in bold reflect test size of 0.13 or less)

Years	Trend (p)	Subpop Trend	Sampling Design	Revisit Design	Sites	PO	SLRDB	WLRDB	PWIGLS A-only	PWIGLS A	PWIGLS AI	PWIGLS B	PWIGLS BI	PWIGLS C
12	0.02	0	SRS	1-0	20	**0.53**	**0.57**	**0.54**	0.92	0.92	0.92	0.92	0.92	0.92
12	0.02	0	SRS	1-0	35	**0.54**	**0.57**	**0.55**	0.94	0.94	0.94	0.94	0.94	0.94
12	0.02	0	SRS	1-0	50	**0.54**	**0.56**	0.56	0.95	0.95	0.95	0.95	0.95	0.95
12	0.02	0	SRS	1-0,1-3	20	**0.56**	**0.48**	**0.45**	0.74	0.77	0.74	0.77	0.74	0.74
12	0.02	0	SRS	1-0,1-3	35	**0.56**	**0.48**	**0.47**	0.85	0.86	0.84	0.86	0.84	0.85
12	0.02	0	SRS	1-0,1-3	50	**0.55**	**0.51**	**0.48**	0.87	0.89	0.87	0.89	0.87	0.87
12	0.02	0	SRS	1-3	20	**0.47**	**0.36**	**0.37**	0.79	0.79	0.79	0.79	0.79	0.79
12	0.02	0	SRS	1-3	35	**0.48**	**0.41**	**0.44**	0.85	0.85	0.85	0.85	0.85	0.85
12	0.02	0	SRS	1-3	50	**0.48**	**0.43**	**0.48**	0.89	0.89	0.89	0.89	0.89	0.89
12	0.02	0	StRS	1-0	20	**0.74**	**0.61**	**0.62**	0.87	0.87	0.87	0.87	0.87	0.87
12	0.02	0	StRS	1-0	35	**0.77**	**0.65**	**0.63**	0.90	0.90	0.90	0.90	0.90	0.90
12	0.02	0	StRS	1-0	50	**0.75**	**0.64**	**0.64**	0.91	0.91	0.91	0.91	0.91	0.91
12	0.02	0	StRS	1-0,1-3	20	**0.74**	**0.42**	**0.47**	0.67	0.66	0.67	0.66	0.67	0.67
12	0.02	0	StRS	1-0,1-3	35	**0.77**	**0.46**	**0.48**	0.76	0.74	0.76	0.74	0.76	0.76
12	0.02	0	StRS	1-0,1-3	50	**0.78**	**0.52**	**0.50**	0.81	0.77	0.82	0.77	0.82	0.81
12	0.02	0	StRS	1-3	20	**0.70**	**0.33**	**0.40**	0.70	0.70	0.70	0.70	0.70	0.70
12	0.02	0	StRS	1-3	35	**0.73**	**0.39**	**0.47**	0.79	0.79	0.80	0.79	0.80	0.79
12	0.02	0	StRS	1-3	50	**0.73**	**0.48**	**0.48**	0.80	0.79	0.82	0.79	0.82	0.79
12	0.02	0	Unequal	1-0	20	**0.53**	**0.54**	**0.54**	0.87	0.87	0.87	0.87	0.87	0.87
12	0.02	0	Unequal	1-0	35	**0.56**	**0.57**	**0.55**	0.90	0.90	0.90	0.90	0.90	0.90
12	0.02	0	Unequal	1-0	50	**0.57**	**0.60**	**0.54**	0.91	0.91	0.91	0.91	0.91	0.91
12	0.02	0	Unequal	1-0,1-3	20	**0.54**	**0.37**	**0.40**	0.63	0.60	0.63	0.60	0.63	0.63
12	0.02	0	Unequal	1-0,1-3	35	**0.55**	**0.39**	0.42	0.70	0.68	0.70	0.68	0.70	0.70
12	0.02	0	Unequal	1-0,1-3	50	**0.55**	**0.42**	**0.42**	0.74	0.73	0.74	0.73	0.74	0.74
12	0.02	0	Unequal	1-3	20	**0.50**	**0.26**	**0.38**	0.60	0.60	0.60	0.60	0.60	0.60
12	0.02	0	Unequal	1-3	35	**0.51**	**0.32**	**0.39**	0.70	0.70	0.71	0.70	0.71	0.70
12	0.02	0	Unequal	1-3	50	**0.51**	**0.38**	**0.40**	0.77	0.77	0.78	0.77	0.78	0.77
12	0.02	0.04	SRS	1-0	20	**1.00**	1.00	**0.99**	1.00	1.00	1.00	1.00	1.00	1.00
12	0.02	0.04	SRS	1-0	35	**1.00**	1.00	**1.00**	1.00	1.00	1.00	1.00	1.00	1.00
12	0.02	0.04	SRS	1-0	50	**1.00**	1.00	1.00	1.00	1.00	1.00	1.00	1.00	1.00
12	0.02	0.04	SRS	1-0,1-3	20	**1.00**	**0.99**	**0.98**	1.00	1.00	1.00	1.00	1.00	1.00
12	0.02	0.04	SRS	1-0,1-3	35	**1.00**	**0.99**	**1.00**	1.00	1.00	1.00	1.00	1.00	1.00
12	0.02	0.04	SRS	1-0,1-3	50	**1.00**	**1.00**	**0.98**	1.00	1.00	1.00	1.00	1.00	1.00
12	0.02	0.04	SRS	1-3	20	**1.00**	**0.96**	**0.97**	1.00	1.00	1.00	1.00	1.00	1.00
12	0.02	0.04	SRS	1-3	35	**1.00**	**0.99**	**0.97**	1.00	1.00	1.00	1.00	1.00	1.00
12	0.02	0.04	SRS	1-3	50	**1.00**	**0.99**	**0.98**	1.00	1.00	1.00	1.00	1.00	1.00

Table B7: Trend Test Power of the *t*-test for the ANC Trend Slope Estimate (shaded cells indicate power below 0.8, results in bold reflect test size of 0.13 or less)

Years	Trend (p)	Subpop Trend	Sampling Design	Revisit Design	Sites	PO	SLRDB	WLRDB	PWIGLS A-only	PWIGLS A	PWIGLS AI	PWIGLS B	PWIGLS BI	PWIGLS C
12	0.02	0.04	StRS	1-0	20	**1.00**	1.00	**1.00**	1.00	1.00	1.00	1.00	1.00	1.00
12	0.02	0.04	StRS	1-0	35	**1.00**	1.00	**1.00**	1.00	1.00	1.00	1.00	1.00	1.00
12	0.02	0.04	StRS	1-0	50	**1.00**	1.00	**1.00**	1.00	1.00	1.00	1.00	1.00	1.00
12	0.02	0.04	StRS	1-0,1-3	20	**1.00**	**0.99**	**0.97**	1.00	1.00	1.00	1.00	1.00	1.00
12	0.02	0.04	StRS	1-0,1-3	35	**1.00**	**1.00**	**0.99**	1.00	1.00	1.00	1.00	1.00	1.00
12	0.02	0.04	StRS	1-0,1-3	50	**1.00**	**1.00**	**0.99**	1.00	1.00	1.00	1.00	1.00	1.00
12	0.02	0.04	StRS	1-3	20	**1.00**	0.97	**0.95**	1.00	1.00	1.00	1.00	1.00	1.00
12	0.02	0.04	StRS	1-3	35	**1.00**	**0.99**	**0.97**	1.00	1.00	1.00	1.00	1.00	1.00
12	0.02	0.04	StRS	1-3	50	**1.00**	**1.00**	**0.99**	1.00	1.00	1.00	1.00	1.00	1.00
12	0.02	0.04	Unequal	1-0	20	**0.99**	1.00	**0.99**	1.00	1.00	1.00	1.00	1.00	1.00
12	0.02	0.04	Unequal	1-0	35	**0.99**	1.00	**0.99**	1.00	1.00	1.00	1.00	1.00	1.00
12	0.02	0.04	Unequal	1-0	50	**0.99**	1.00	**0.98**	1.00	1.00	1.00	1.00	1.00	1.00
12	0.02	0.04	Unequal	1-0,1-3	20	**1.00**	**0.96**	**0.93**	0.99	1.00	0.99	1.00	0.99	0.99
12	0.02	0.04	Unequal	1-0,1-3	35	**1.00**	**0.97**	0.98	1.00	1.00	1.00	1.00	1.00	1.00
12	0.02	0.04	Unequal	1-0,1-3	50	**1.00**	**0.99**	**0.97**	1.00	1.00	1.00	1.00	1.00	1.00
12	0.02	0.04	Unequal	1-3	20	**0.99**	0.89	**0.90**	1.00	1.00	1.00	1.00	1.00	1.00
12	0.02	0.04	Unequal	1-3	35	**0.99**	0.95	**0.93**	1.00	1.00	1.00	1.00	1.00	1.00
12	0.02	0.04	Unequal	1-3	50	**1.00**	**0.98**	**0.95**	1.00	1.00	1.00	1.00	1.00	1.00
24	0.01	0	SRS	1-0	20	**0.71**	0.87	0.86	0.92	0.92	0.92	0.92	0.92	0.92
24	0.01	0	SRS	1-0	35	**0.79**	0.88	0.89	0.96	0.96	0.96	0.96	0.96	0.96
24	0.01	0	SRS	1-0	50	**0.76**	0.89	0.88	0.96	0.96	0.96	0.96	0.96	0.96
24	0.01	0	SRS	1-0,1-3	20	**0.75**	**0.77**	0.76	0.90	0.92	0.90	0.92	0.90	0.90
24	0.01	0	SRS	1-0,1-3	35	**0.78**	**0.81**	**0.83**	0.95	0.95	0.95	0.95	0.95	0.95
24	0.01	0	SRS	1-0,1-3	50	**0.79**	**0.83**	0.83	0.96	0.97	0.96	0.97	0.96	0.96
24	0.01	0	SRS	1-3	20	**0.78**	**0.71**	0.68	0.93	0.93	0.93	0.93	0.93	0.93
24	0.01	0	SRS	1-3	35	**0.80**	**0.79**	0.76	0.96	0.96	0.96	0.96	0.96	0.96
24	0.01	0	SRS	1-3	50	**0.79**	**0.82**	0.81	0.97	0.97	0.97	0.97	0.97	0.97
24	0.01	0	StRS	1-0	20	**0.86**	0.88	0.87	0.89	0.89	0.89	0.89	0.89	0.89
24	0.01	0	StRS	1-0	35	**0.94**	0.91	0.89	0.94	0.94	0.94	0.94	0.94	0.94
24	0.01	0	StRS	1-0	50	**0.95**	0.93	0.90	0.95	0.95	0.95	0.95	0.95	0.95
24	0.01	0	StRS	1-0,1-3	20	**0.93**	**0.78**	0.75	0.92	0.89	0.93	0.89	0.93	0.92
24	0.01	0	StRS	1-0,1-3	35	**0.96**	**0.83**	**0.78**	0.95	0.93	0.96	0.93	0.96	0.95
24	0.01	0	StRS	1-0,1-3	50	**0.97**	**0.87**	0.80	0.96	0.96	0.96	0.96	0.96	0.96
24	0.01	0	StRS	1-3	20	**0.96**	**0.70**	**0.67**	0.94	0.94	0.94	0.94	0.94	0.94
24	0.01	0	StRS	1-3	35	**0.98**	**0.81**	**0.73**	0.96	0.96	0.96	0.96	0.96	0.96
24	0.01	0	StRS	1-3	50	**0.97**	**0.85**	**0.80**	0.95	0.95	0.96	0.95	0.96	0.95

Table B7: Trend Test Power of the *t*-test for the ANC Trend Slope Estimate (shaded cells indicate power below 0.8, results in bold reflect test size of 0.13 or less)

Years	Trend (p)	Subpop Trend	Sampling Design	Revisit Design	Sites	PO	SLRDB	WLRDB	PWIGLS A-only	PWIGLS A	PWIGLS AI	PWIGLS B	PWIGLS BI	PWIGLS C
24	0.01	0	Unequal	1-0	20	**0.70**	0.80	0.82	0.80	0.80	0.80	0.80	0.80	0.80
24	0.01	0	Unequal	1-0	35	**0.74**	0.85	0.86	0.88	0.88	0.88	0.88	0.88	0.88
24	0.01	0	Unequal	1-0	50	**0.78**	0.86	0.85	0.89	0.89	0.89	0.89	0.89	0.89
24	0.01	0	Unequal	1-0,1-3	20	**0.77**	**0.66**	0.68	0.77	0.73	0.77	0.73	0.77	0.77
24	0.01	0	Unequal	1-0,1-3	35	**0.82**	**0.70**	0.72	0.90	0.87	0.91	0.87	0.91	0.90
24	0.01	0	Unequal	1-0,1-3	50	**0.81**	**0.77**	**0.75**	0.93	0.92	0.94	0.92	0.94	0.93
24	0.01	0	Unequal	1-3	20	**0.81**	**0.59**	0.56	0.88	0.88	0.88	0.88	0.88	0.88
24	0.01	0	Unequal	1-3	35	**0.84**	**0.68**	0.61	0.88	0.88	0.89	0.88	0.89	0.88
24	0.01	0	Unequal	1-3	50	**0.83**	**0.74**	**0.69**	0.92	0.92	0.92	0.92	0.92	0.92
24	0.01	0.04	SRS	1-0	20	**1.00**	1.00	1.00	1.00	1.00	1.00	1.00	1.00	1.00
24	0.01	0.04	SRS	1-0	35	**1.00**	1.00	1.00	1.00	1.00	1.00	1.00	1.00	1.00
24	0.01	0.04	SRS	1-0	50	**1.00**	1.00	1.00	1.00	1.00	1.00	1.00	1.00	1.00
24	0.01	0.04	SRS	1-0,1-3	20	**1.00**	**1.00**	1.00	1.00	1.00	1.00	1.00	1.00	1.00
24	0.01	0.04	SRS	1-0,1-3	35	**1.00**	**1.00**	**1.00**	1.00	1.00	1.00	1.00	1.00	1.00
24	0.01	0.04	SRS	1-0,1-3	50	**1.00**	**1.00**	1.00	1.00	1.00	1.00	1.00	1.00	1.00
24	0.01	0.04	SRS	1-3	20	**1.00**	**1.00**	1.00	1.00	1.00	1.00	1.00	1.00	1.00
24	0.01	0.04	SRS	1-3	35	**1.00**	**1.00**	1.00	1.00	1.00	1.00	1.00	1.00	1.00
24	0.01	0.04	SRS	1-3	50	**1.00**	**1.00**	1.00	1.00	1.00	1.00	1.00	1.00	1.00
24	0.01	0.04	StRS	1-0	20	**1.00**	1.00	1.00	1.00	1.00	1.00	1.00	1.00	1.00
24	0.01	0.04	StRS	1-0	35	**1.00**	1.00	1.00	1.00	1.00	1.00	1.00	1.00	1.00
24	0.01	0.04	StRS	1-0	50	**1.00**	1.00	1.00	1.00	1.00	1.00	1.00	1.00	1.00
24	0.01	0.04	StRS	1-0,1-3	20	**1.00**	**1.00**	1.00	1.00	1.00	1.00	1.00	1.00	1.00
24	0.01	0.04	StRS	1-0,1-3	35	**1.00**	**1.00**	**1.00**	1.00	1.00	1.00	1.00	1.00	1.00
24	0.01	0.04	StRS	1-0,1-3	50	**1.00**	**1.00**	1.00	1.00	1.00	1.00	1.00	1.00	1.00
24	0.01	0.04	StRS	1-3	20	**1.00**	**1.00**	**1.00**	1.00	1.00	1.00	1.00	1.00	1.00
24	0.01	0.04	StRS	1-3	35	**1.00**	**1.00**	**1.00**	1.00	1.00	1.00	1.00	1.00	1.00
24	0.01	0.04	StRS	1-3	50	**1.00**	**1.00**	**1.00**	1.00	1.00	1.00	1.00	1.00	1.00
24	0.01	0.04	Unequal	1-0	20	**1.00**	1.00	1.00	1.00	1.00	1.00	1.00	1.00	1.00
24	0.01	0.04	Unequal	1-0	35	**1.00**	1.00	1.00	1.00	1.00	1.00	1.00	1.00	1.00
24	0.01	0.04	Unequal	1-0	50	**1.00**	1.00	1.00	1.00	1.00	1.00	1.00	1.00	1.00
24	0.01	0.04	Unequal	1-0,1-3	20	**1.00**	**1.00**	1.00	1.00	1.00	1.00	1.00	1.00	1.00
24	0.01	0.04	Unequal	1-0,1-3	35	**1.00**	**1.00**	1.00	1.00	1.00	1.00	1.00	1.00	1.00
24	0.01	0.04	Unequal	1-0,1-3	50	**1.00**	**1.00**	**1.00**	1.00	1.00	1.00	1.00	1.00	1.00
24	0.01	0.04	Unequal	1-3	20	**1.00**	**1.00**	1.00	1.00	1.00	1.00	1.00	1.00	1.00
24	0.01	0.04	Unequal	1-3	35	**1.00**	**1.00**	1.00	1.00	1.00	1.00	1.00	1.00	1.00
24	0.01	0.04	Unequal	1-3	50	**1.00**	**1.00**	**1.00**	1.00	1.00	1.00	1.00	1.00	1.00

Table B8: Trend Test Power of the *t*-test for the modified ANC Trend Slope Estimate (shaded cells indicate power below 0.8, results in bold reflect test size of 0.13 or less)

Years	Trend (p)	Subpop Trend	Sampling Design	Revisit Design	Sites	PO	SLRDB	WLRDB	PWIGLS A-only	PWIGLS A	PWIGLS AI	PWIGLS B	PWIGLS BI	PWIGLS C
12	0.02	0	SRS	1-0	20	**1.00**	1.00	1.00	**1.00**	**1.00**	**1.00**	**1.00**	**1.00**	**1.00**
12	0.02	0	SRS	1-0	35	**1.00**	1.00	1.00	**1.00**	**1.00**	**1.00**	**1.00**	**1.00**	**1.00**
12	0.02	0	SRS	1-0	50	**1.00**	1.00	1.00	**1.00**	**1.00**	**1.00**	**1.00**	**1.00**	**1.00**
12	0.02	0	SRS	1-0,1-3	20	**1.00**	**0.81**	**0.84**	**0.98**	**0.98**	**0.98**	**0.98**	**0.98**	**0.98**
12	0.02	0	SRS	1-0,1-3	35	**1.00**	**0.90**	**0.90**	**1.00**	**1.00**	**1.00**	**1.00**	**1.00**	**1.00**
12	0.02	0	SRS	1-0,1-3	50	**1.00**	**0.96**	**0.94**	**1.00**	**1.00**	**1.00**	**1.00**	**1.00**	**1.00**
12	0.02	0	SRS	1-3	20	**1.00**	**0.66**	**0.65**	**1.00**	**1.00**	**1.00**	**1.00**	**1.00**	**1.00**
12	0.02	0	SRS	1-3	35	**1.00**	**0.84**	**0.85**	**1.00**	**1.00**	**1.00**	**1.00**	**1.00**	**1.00**
12	0.02	0	SRS	1-3	50	**1.00**	**0.89**	**0.92**	**1.00**	**1.00**	**1.00**	**1.00**	**1.00**	**1.00**
12	0.02	0	StRS	1-0	20	**1.00**	1.00	1.00	**1.00**	**1.00**	**1.00**	**1.00**	**1.00**	**1.00**
12	0.02	0	StRS	1-0	35	**1.00**	1.00	1.00	**1.00**	**1.00**	**1.00**	**1.00**	**1.00**	**1.00**
12	0.02	0	StRS	1-0	50	**1.00**	1.00	1.00	**1.00**	**1.00**	**1.00**	**1.00**	**1.00**	**1.00**
12	0.02	0	StRS	1-0,1-3	20	**1.00**	**0.76**	**0.80**	**0.96**	**0.96**	**0.96**	**0.96**	**0.96**	**0.96**
12	0.02	0	StRS	1-0,1-3	35	**1.00**	**0.81**	**0.81**	**1.00**	**1.00**	**1.00**	**1.00**	**1.00**	**1.00**
12	0.02	0	StRS	1-0,1-3	50	**1.00**	**0.87**	**0.89**	**1.00**	**1.00**	**1.00**	**1.00**	**1.00**	**1.00**
12	0.02	0	StRS	1-3	20	**1.00**	**0.56**	**0.58**	**0.98**	**0.98**	**0.98**	**0.98**	**0.98**	**0.98**
12	0.02	0	StRS	1-3	35	**1.00**	**0.71**	**0.73**	**1.00**	**1.00**	**1.00**	**1.00**	**1.00**	**1.00**
12	0.02	0	StRS	1-3	50	**1.00**	**0.81**	**0.84**	**1.00**	**1.00**	**1.00**	**1.00**	**1.00**	**1.00**
12	0.02	0	Unequal	1-0	20	**1.00**	0.99	1.00	**0.99**	**0.99**	**0.99**	**0.99**	**0.99**	**0.99**
12	0.02	0	Unequal	1-0	35	**1.00**	1.00	1.00	**1.00**	**1.00**	**1.00**	**1.00**	**1.00**	**1.00**
12	0.02	0	Unequal	1-0	50	**1.00**	1.00	1.00	**1.00**	**1.00**	**1.00**	**1.00**	**1.00**	**1.00**
12	0.02	0	Unequal	1-0,1-3	20	**1.00**	**0.59**	**0.71**	**0.73**	**0.73**	**0.74**	**0.73**	**0.74**	**0.73**
12	0.02	0	Unequal	1-0,1-3	35	**1.00**	**0.68**	**0.76**	**0.96**	**0.96**	**0.96**	**0.96**	**0.96**	**0.96**
12	0.02	0	Unequal	1-0,1-3	50	**1.00**	**0.71**	**0.78**	**1.00**	**1.00**	**1.00**	**1.00**	**1.00**	**1.00**
12	0.02	0	Unequal	1-3	20	**1.00**	**0.42**	**0.49**	**0.83**	**0.83**	**0.83**	**0.83**	**0.83**	**0.83**
12	0.02	0	Unequal	1-3	35	**1.00**	**0.50**	**0.61**	**0.97**	**0.97**	**0.98**	**0.97**	**0.98**	**0.97**
12	0.02	0	Unequal	1-3	50	**1.00**	**0.65**	**0.75**	**0.99**	**0.99**	**1.00**	**0.99**	**1.00**	**0.99**
12	0.02	0.04	SRS	1-0	20	**1.00**	1.00	1.00	**1.00**	**1.00**	**1.00**	**1.00**	**1.00**	**1.00**
12	0.02	0.04	SRS	1-0	35	**1.00**	1.00	1.00	**1.00**	**1.00**	**1.00**	**1.00**	**1.00**	**1.00**
12	0.02	0.04	SRS	1-0	50	**1.00**	1.00	1.00	**1.00**	**1.00**	**1.00**	**1.00**	**1.00**	**1.00**
12	0.02	0.04	SRS	1-0,1-3	20	**1.00**	**1.00**	**1.00**	**1.00**	**1.00**	**1.00**	**1.00**	**1.00**	**1.00**
12	0.02	0.04	SRS	1-0,1-3	35	**1.00**	**1.00**	**1.00**	**1.00**	**1.00**	**1.00**	**1.00**	**1.00**	**1.00**
12	0.02	0.04	SRS	1-0,1-3	50	**1.00**	**1.00**	**1.00**	**1.00**	**1.00**	**1.00**	**1.00**	**1.00**	**1.00**
12	0.02	0.04	SRS	1-3	20	**1.00**	**1.00**	**1.00**	**1.00**	**1.00**	**1.00**	**1.00**	**1.00**	**1.00**
12	0.02	0.04	SRS	1-3	35	**1.00**	**1.00**	**1.00**	**1.00**	**1.00**	**1.00**	**1.00**	**1.00**	**1.00**
12	0.02	0.04	SRS	1-3	50	**1.00**	**1.00**	**1.00**	**1.00**	**1.00**	**1.00**	**1.00**	**1.00**	**1.00**
12	0.02	0.04	StRS	1-0	20	**1.00**	1.00	1.00	**1.00**	**1.00**	**1.00**	**1.00**	**1.00**	**1.00**

Table B8: Trend Test Power of the *t*-test for the modified ANC Trend Slope Estimate (shaded cells indicate power below 0.8, results in bold reflect test size of 0.13 or less)

Years	Trend (p)	Subpop Trend	Sampling Design	Revisit Design	Sites	PO	SLRDB	WLRDB	PWIGLS A-only	PWIGLS A	PWIGLS AI	PWIGLS B	PWIGLS BI	PWIGLS C
12	0.02	0.04	StRS	1-0	35	**1.00**	1.00	1.00	**1.00**	**1.00**	**1.00**	**1.00**	**1.00**	**1.00**
12	0.02	0.04	StRS	1-0	50	**1.00**	1.00	1.00	**1.00**	**1.00**	**1.00**	**1.00**	**1.00**	**1.00**
12	0.02	0.04	StRS	1-0,1-3	20	**1.00**	**1.00**	**1.00**	**1.00**	**1.00**	**1.00**	**1.00**	**1.00**	**1.00**
12	0.02	0.04	StRS	1-0,1-3	35	**1.00**	**1.00**	**1.00**	**1.00**	**1.00**	**1.00**	**1.00**	**1.00**	**1.00**
12	0.02	0.04	StRS	1-0,1-3	50	**1.00**	**1.00**	**1.00**	**1.00**	**1.00**	**1.00**	**1.00**	**1.00**	**1.00**
12	0.02	0.04	StRS	1-3	20	**1.00**	**0.99**	**0.98**	**1.00**	**1.00**	**1.00**	**1.00**	**1.00**	**1.00**
12	0.02	0.04	StRS	1-3	35	**1.00**	**1.00**	**1.00**	**1.00**	**1.00**	**1.00**	**1.00**	**1.00**	**1.00**
12	0.02	0.04	StRS	1-3	50	**1.00**	**1.00**	**1.00**	**1.00**	**1.00**	**1.00**	**1.00**	**1.00**	**1.00**
12	0.02	0.04	Unequal	1-0	20	**1.00**	1.00	1.00	**1.00**	**1.00**	**1.00**	**1.00**	**1.00**	**1.00**
12	0.02	0.04	Unequal	1-0	35	**1.00**	1.00	1.00	**1.00**	**1.00**	**1.00**	**1.00**	**1.00**	**1.00**
12	0.02	0.04	Unequal	1-0	50	**1.00**	1.00	1.00	**1.00**	**1.00**	**1.00**	**1.00**	**1.00**	**1.00**
12	0.02	0.04	Unequal	1-0,1-3	20	**1.00**	**0.99**	**0.99**	**1.00**	**1.00**	**1.00**	**1.00**	**1.00**	**1.00**
12	0.02	0.04	Unequal	1-0,1-3	35	**1.00**	**0.99**	**1.00**	**1.00**	**1.00**	**1.00**	**1.00**	**1.00**	**1.00**
12	0.02	0.04	Unequal	1-0,1-3	50	**1.00**	**1.00**	**1.00**	**1.00**	**1.00**	**1.00**	**1.00**	**1.00**	**1.00**
12	0.02	0.04	Unequal	1-3	20	**1.00**	**0.95**	**0.97**	**1.00**	**1.00**	**1.00**	**1.00**	**1.00**	**1.00**
12	0.02	0.04	Unequal	1-3	35	**1.00**	**0.98**	**0.99**	**1.00**	**1.00**	**1.00**	**1.00**	**1.00**	**1.00**
12	0.02	0.04	Unequal	1-3	50	**1.00**	**1.00**	**1.00**	**1.00**	**1.00**	**1.00**	**1.00**	**1.00**	**1.00**
24	0.01	0	SRS	1-0	20	**0.99**	1.00	1.00	**0.99**	**0.99**	**0.99**	**0.99**	**0.99**	**0.99**
24	0.01	0	SRS	1-0	35	**1.00**	1.00	1.00	**1.00**	**1.00**	**1.00**	**1.00**	**1.00**	**1.00**
24	0.01	0	SRS	1-0	50	**1.00**	1.00	1.00	**1.00**	**1.00**	**1.00**	**1.00**	**1.00**	**1.00**
24	0.01	0	SRS	1-0,1-3	20	**1.00**	0.99	0.97	**1.00**	**1.00**	**1.00**	**1.00**	**1.00**	**1.00**
24	0.01	0	SRS	1-0,1-3	35	**1.00**	1.00	1.00	**1.00**	**1.00**	**1.00**	**1.00**	**1.00**	**1.00**
24	0.01	0	SRS	1-0,1-3	50	**1.00**	1.00	1.00	**1.00**	**1.00**	**1.00**	**1.00**	**1.00**	**1.00**
24	0.01	0	SRS	1-3	20	**1.00**	**0.94**	**0.94**	**1.00**	**1.00**	**1.00**	**1.00**	**1.00**	**1.00**
24	0.01	0	SRS	1-3	35	**1.00**	**0.99**	**0.99**	**1.00**	**1.00**	**1.00**	**1.00**	**1.00**	**1.00**
24	0.01	0	SRS	1-3	50	**1.00**	1.00	**1.00**	**1.00**	**1.00**	**1.00**	**1.00**	**1.00**	**1.00**
24	0.01	0	StRS	1-0	20	**0.99**	1.00	1.00	**0.96**	**0.96**	**0.96**	**0.96**	**0.96**	**0.96**
24	0.01	0	StRS	1-0	35	**1.00**	1.00	1.00	**1.00**	**1.00**	**1.00**	**1.00**	**1.00**	**1.00**
24	0.01	0	StRS	1-0	50	**1.00**	1.00	1.00	1.00	1.00	1.00	1.00	1.00	1.00
24	0.01	0	StRS	1-0,1-3	20	**1.00**	0.94	0.94	**1.00**	**1.00**	**1.00**	**1.00**	**1.00**	**1.00**
24	0.01	0	StRS	1-0,1-3	35	**1.00**	0.98	0.99	**1.00**	**1.00**	**1.00**	**1.00**	**1.00**	**1.00**
24	0.01	0	StRS	1-0,1-3	50	**1.00**	0.99	**0.99**	**1.00**	**1.00**	**1.00**	**1.00**	**1.00**	**1.00**
24	0.01	0	StRS	1-3	20	**1.00**	**0.87**	**0.82**	**1.00**	**1.00**	**1.00**	**1.00**	**1.00**	**1.00**
24	0.01	0	StRS	1-3	35	**1.00**	**0.98**	**0.95**	**1.00**	**1.00**	**1.00**	**1.00**	**1.00**	**1.00**
24	0.01	0	StRS	1-3	50	**1.00**	**0.99**	**0.98**	**1.00**	**1.00**	**1.00**	**1.00**	**1.00**	**1.00**
24	0.01	0	Unequal	1-0	20	**0.99**	0.99	0.99	0.91	0.91	0.91	0.91	0.91	0.91
24	0.01	0	Unequal	1-0	35	**1.00**	1.00	1.00	**0.97**	**0.97**	**0.97**	**0.97**	**0.97**	**0.97**

Table B8: Trend Test Power of the *t*-test for the modified ANC Trend Slope Estimate (shaded cells indicate power below 0.8, results in bold reflect test size of 0.13 or less)

Years	Trend (p)	Subpop Trend	Sampling Design	Revisit Design	Sites	PO	SLRDB	WLRDB	PWIGLS A-only	PWIGLS A	PWIGLS AI	PWIGLS B	PWIGLS BI	PWIGLS C
24	0.01	0	Unequal	1-0	50	**1.00**	1.00	1.00	**0.98**	**0.98**	**0.98**	**0.98**	**0.98**	**0.98**
24	0.01	0	Unequal	1-0,1-3	20	**1.00**	0.86	0.89	**0.95**	**0.95**	**0.95**	**0.95**	**0.95**	**0.95**
24	0.01	0	Unequal	1-0,1-3	35	**1.00**	**0.92**	**0.94**	**1.00**	**1.00**	**1.00**	**1.00**	**1.00**	**1.00**
24	0.01	0	Unequal	1-0,1-3	50	**1.00**	**0.94**	**0.96**	**1.00**	**1.00**	**1.00**	**1.00**	**1.00**	**1.00**
24	0.01	0	Unequal	1-3	20	**1.00**	**0.72**	**0.80**	**1.00**	**1.00**	**1.00**	**1.00**	**1.00**	**1.00**
24	0.01	0	Unequal	1-3	35	**1.00**	**0.87**	**0.89**	**1.00**	**1.00**	**1.00**	**1.00**	**1.00**	**1.00**
24	0.01	0	Unequal	1-3	50	**1.00**	**0.94**	**0.96**	**1.00**	**1.00**	**1.00**	**1.00**	**1.00**	**1.00**
24	0.01	0.04	SRS	1-0	20	**1.00**	1.00	1.00	**1.00**	**1.00**	**1.00**	**1.00**	**1.00**	**1.00**
24	0.01	0.04	SRS	1-0	35	**1.00**	1.00	1.00	**1.00**	**1.00**	**1.00**	**1.00**	**1.00**	**1.00**
24	0.01	0.04	SRS	1-0	50	**1.00**	1.00	1.00	**1.00**	**1.00**	**1.00**	**1.00**	**1.00**	**1.00**
24	0.01	0.04	SRS	1-0,1-3	20	**1.00**	1.00	1.00	**1.00**	**1.00**	**1.00**	**1.00**	**1.00**	**1.00**
24	0.01	0.04	SRS	1-0,1-3	35	**1.00**	1.00	1.00	**1.00**	**1.00**	**1.00**	**1.00**	**1.00**	**1.00**
24	0.01	0.04	SRS	1-0,1-3	50	**1.00**	1.00	1.00	**1.00**	**1.00**	**1.00**	**1.00**	**1.00**	**1.00**
24	0.01	0.04	SRS	1-3	20	**1.00**	**1.00**	**1.00**	**1.00**	**1.00**	**1.00**	**1.00**	**1.00**	**1.00**
24	0.01	0.04	SRS	1-3	35	**1.00**	**1.00**	**1.00**	**1.00**	**1.00**	**1.00**	**1.00**	**1.00**	**1.00**
24	0.01	0.04	SRS	1-3	50	**1.00**	1.00	**1.00**	**1.00**	**1.00**	**1.00**	**1.00**	**1.00**	**1.00**
24	0.01	0.04	StRS	1-0	20	**1.00**	1.00	1.00	**1.00**	**1.00**	**1.00**	**1.00**	**1.00**	**1.00**
24	0.01	0.04	StRS	1-0	35	**1.00**	1.00	1.00	**1.00**	**1.00**	**1.00**	**1.00**	**1.00**	**1.00**
24	0.01	0.04	StRS	1-0	50	**1.00**	1.00	1.00	1.00	1.00	1.00	1.00	1.00	1.00
24	0.01	0.04	StRS	1-0,1-3	20	**1.00**	1.00	1.00	**1.00**	**1.00**	**1.00**	**1.00**	**1.00**	**1.00**
24	0.01	0.04	StRS	1-0,1-3	35	**1.00**	1.00	1.00	**1.00**	**1.00**	**1.00**	**1.00**	**1.00**	**1.00**
24	0.01	0.04	StRS	1-0,1-3	50	**1.00**	1.00	**1.00**	**1.00**	**1.00**	**1.00**	**1.00**	**1.00**	**1.00**
24	0.01	0.04	StRS	1-3	20	**1.00**	**1.00**	**1.00**	**1.00**	**1.00**	**1.00**	**1.00**	**1.00**	**1.00**
24	0.01	0.04	StRS	1-3	35	**1.00**	**1.00**	**1.00**	**1.00**	**1.00**	**1.00**	**1.00**	**1.00**	**1.00**
24	0.01	0.04	StRS	1-3	50	**1.00**	**1.00**	**1.00**	**1.00**	**1.00**	**1.00**	**1.00**	**1.00**	**1.00**
24	0.01	0.04	Unequal	1-0	20	**1.00**	1.00	1.00	1.00	1.00	1.00	1.00	1.00	1.00
24	0.01	0.04	Unequal	1-0	35	**1.00**	1.00	1.00	**1.00**	**1.00**	**1.00**	**1.00**	**1.00**	**1.00**
24	0.01	0.04	Unequal	1-0	50	**1.00**	1.00	1.00	**1.00**	**1.00**	**1.00**	**1.00**	**1.00**	**1.00**
24	0.01	0.04	Unequal	1-0,1-3	20	**1.00**	1.00	1.00	**1.00**	**1.00**	**1.00**	**1.00**	**1.00**	**1.00**
24	0.01	0.04	Unequal	1-0,1-3	35	**1.00**	**1.00**	**1.00**	**1.00**	**1.00**	**1.00**	**1.00**	**1.00**	**1.00**
24	0.01	0.04	Unequal	1-0,1-3	50	**1.00**	**1.00**	**1.00**	**1.00**	**1.00**	**1.00**	**1.00**	**1.00**	**1.00**
24	0.01	0.04	Unequal	1-3	20	**1.00**	**1.00**	**1.00**	**1.00**	**1.00**	**1.00**	**1.00**	**1.00**	**1.00**
24	0.01	0.04	Unequal	1-3	35	**1.00**	**1.00**	**1.00**	**1.00**	**1.00**	**1.00**	**1.00**	**1.00**	**1.00**
24	0.01	0.04	Unequal	1-3	50	**1.00**	**1.00**	**1.00**	**1.00**	**1.00**	**1.00**	**1.00**	**1.00**	**1.00**

Table B9: Relative Bias of the ANC Site-to-Site Variance Estimate (shaded cells indicate > 5% or < -5% relative bias)

Years	Trend (p)	Subpop Trend	Sampling Design	Revisit Design	Sites	PO	PWIGLS A-only	PWIGLS A	PWIGLS AI	PWIGLS B	PWIGLS BI	PWIGLS C
12	0.02	0	SRS	1-0	20	3.34%	3260.02%	3260.02%	3260.02%	3260.02%	3260.02%	3260.02%
12	0.02	0	SRS	1-0	35	-0.53%	1789.54%	1789.54%	1789.54%	1789.54%	1789.54%	1789.54%
12	0.02	0	SRS	1-0	50	2.61%	1275.98%	1275.98%	1275.98%	1275.98%	1275.98%	1275.98%
12	0.02	0	SRS	1-0,1-3	20	0.02%	1241.12%	1240.90%	4426.15%	1240.90%	4426.15%	1241.12%
12	0.02	0	SRS	1-0,1-3	35	-0.14%	515.30%	515.03%	2172.25%	515.03%	2172.25%	515.30%
12	0.02	0	SRS	1-0,1-3	50	-0.32%	297.93%	298.40%	1409.81%	298.40%	1409.81%	297.93%
12	0.02	0	SRS	1-3	20	0.70%	750.93%	750.93%	3303.73%	750.93%	3303.73%	750.93%
12	0.02	0	SRS	1-3	35	-0.05%	384.91%	384.91%	1839.66%	384.91%	1839.66%	384.91%
12	0.02	0	SRS	1-3	50	0.23%	241.00%	241.00%	1263.99%	241.00%	1263.99%	241.00%
12	0.02	0	StRS	1-0	20	1.30%	3036.05%	3036.05%	3036.05%	3036.05%	3036.05%	3036.05%
12	0.02	0	StRS	1-0	35	-1.84%	1693.13%	1693.13%	1693.13%	1693.13%	1693.13%	1693.13%
12	0.02	0	StRS	1-0	50	-0.56%	1194.79%	1194.79%	1194.79%	1194.79%	1194.79%	1194.79%
12	0.02	0	StRS	1-0,1-3	20	-0.47%	1195.07%	1193.38%	4215.95%	1193.38%	4215.95%	1195.07%
12	0.02	0	StRS	1-0,1-3	35	-0.68%	493.61%	493.30%	2096.51%	493.30%	2096.51%	493.61%
12	0.02	0	StRS	1-0,1-3	50	-0.54%	286.64%	287.04%	1372.99%	287.04%	1372.99%	286.64%
12	0.02	0	StRS	1-3	20	0.34%	717.42%	717.44%	3169.68%	717.44%	3169.68%	717.44%
12	0.02	0	StRS	1-3	35	0.68%	377.01%	377.02%	1808.07%	377.02%	1808.07%	377.02%
12	0.02	0	StRS	1-3	50	0.43%	232.93%	232.93%	1231.72%	232.93%	1231.72%	232.93%
12	0.02	0	Unequal	1-0	20	-2.79%	2582.55%	2582.55%	2582.55%	2582.55%	2582.55%	2582.55%
12	0.02	0	Unequal	1-0	35	0.92%	1662.25%	1662.25%	1662.25%	1662.25%	1662.25%	1662.25%
12	0.02	0	Unequal	1-0	50	-0.33%	1164.46%	1164.46%	1164.46%	1164.46%	1164.46%	1164.46%
12	0.02	0	Unequal	1-0,1-3	20	-0.40%	1121.78%	1123.45%	3923.26%	1123.45%	3923.26%	1121.78%
12	0.02	0	Unequal	1-0,1-3	35	0.20%	479.29%	481.25%	2040.27%	481.25%	2040.27%	479.29%
12	0.02	0	Unequal	1-0,1-3	50	-0.47%	274.49%	275.83%	1316.37%	275.83%	1316.37%	274.49%
12	0.02	0	Unequal	1-3	20	-0.73%	701.99%	701.99%	3107.95%	701.99%	3107.95%	701.99%
12	0.02	0	Unequal	1-3	35	-1.33%	351.69%	351.69%	1706.76%	351.69%	1706.76%	351.69%
12	0.02	0	Unequal	1-3	50	-0.57%	219.79%	219.79%	1179.15%	219.79%	1179.15%	219.79%
12	0.02	0.04	SRS	1-0	20	-1.50%	3104.46%	3104.46%	3104.46%	3104.46%	3104.46%	3104.46%
12	0.02	0.04	SRS	1-0	35	-0.19%	1796.38%	1796.38%	1796.38%	1796.38%	1796.38%	1796.38%
12	0.02	0.04	SRS	1-0	50	0.20%	1243.93%	1243.93%	1243.93%	1243.93%	1243.93%	1243.93%
12	0.02	0.04	SRS	1-0,1-3	20	0.28%	1248.07%	1244.73%	4413.94%	1244.73%	4413.94%	1248.07%
12	0.02	0.04	SRS	1-0,1-3	35	-0.73%	512.37%	511.43%	2147.69%	511.43%	2147.69%	512.37%
12	0.02	0.04	SRS	1-0,1-3	50	-0.39%	297.85%	298.12%	1405.04%	298.12%	1405.04%	297.85%
12	0.02	0.04	SRS	1-3	20	0.44%	748.75%	748.75%	3295.02%	748.75%	3295.02%	748.75%
12	0.02	0.04	SRS	1-3	35	0.01%	385.20%	385.20%	1840.82%	385.20%	1840.82%	385.20%
12	0.02	0.04	SRS	1-3	50	-0.21%	239.44%	239.44%	1257.75%	239.44%	1257.75%	239.44%

Table B9: Relative Bias of the ANC Site-to-Site Variance Estimate (shaded cells indicate > 5% or < -5% relative bias)

Years	Trend (p)	Subpop Trend	Sampling Design	Revisit Design	Sites	PO	PWIGLS A-only	PWIGLS A	PWIGLS AI	PWIGLS B	PWIGLS BI	PWIGLS C
12	0.02	0.04	StRS	1-0	20	-2.88%	2900.04%	2900.04%	2900.04%	2900.04%	2900.04%	2900.04%
12	0.02	0.04	StRS	1-0	35	-0.39%	1736.19%	1736.19%	1736.19%	1736.19%	1736.19%	1736.19%
12	0.02	0.04	StRS	1-0	50	-1.96%	1180.43%	1180.43%	1180.43%	1180.43%	1180.43%	1180.43%
12	0.02	0.04	StRS	1-0,1-3	20	1.04%	1215.43%	1214.41%	4289.29%	1214.41%	4289.29%	1215.47%
12	0.02	0.04	StRS	1-0,1-3	35	-0.06%	498.09%	498.17%	2107.18%	498.17%	2107.18%	498.09%
12	0.02	0.04	StRS	1-0,1-3	50	-0.23%	287.66%	287.78%	1372.71%	287.78%	1372.71%	287.66%
12	0.02	0.04	StRS	1-3	20	0.47%	721.26%	721.28%	3185.12%	721.28%	3185.12%	721.28%
12	0.02	0.04	StRS	1-3	35	-0.08%	374.49%	374.49%	1797.97%	374.49%	1797.97%	374.49%
12	0.02	0.04	StRS	1-3	50	-0.91%	228.41%	228.41%	1213.64%	228.41%	1213.64%	228.41%
12	0.02	0.04	Unequal	1-0	20	-0.83%	2594.00%	2594.00%	2594.00%	2594.00%	2594.00%	2594.00%
12	0.02	0.04	Unequal	1-0	35	1.49%	1731.74%	1731.74%	1731.74%	1731.74%	1731.74%	1731.74%
12	0.02	0.04	Unequal	1-0	50	0.08%	1171.45%	1171.45%	1171.45%	1171.45%	1171.45%	1171.45%
12	0.02	0.04	Unequal	1-0,1-3	20	-0.14%	1108.45%	1110.93%	3890.10%	1110.93%	3890.10%	1108.45%
12	0.02	0.04	Unequal	1-0,1-3	35	-0.07%	480.26%	481.75%	2034.33%	481.75%	2034.33%	480.26%
12	0.02	0.04	Unequal	1-0,1-3	50	0.69%	273.55%	275.06%	1304.58%	275.06%	1304.58%	273.55%
12	0.02	0.04	Unequal	1-3	20	-0.02%	684.01%	684.01%	3036.04%	684.01%	3036.04%	684.01%
12	0.02	0.04	Unequal	1-3	35	-0.53%	351.49%	351.49%	1705.98%	351.49%	1705.98%	351.49%
12	0.02	0.04	Unequal	1-3	50	-0.06%	219.63%	219.63%	1178.53%	219.63%	1178.53%	219.63%
24	0.01	0	SRS	1-0	20	0.45%	3164.54%	3164.54%	3164.54%	3164.54%	3164.54%	3164.54%
24	0.01	0	SRS	1-0	35	0.10%	1799.81%	1799.81%	1799.81%	1799.81%	1799.81%	1799.81%
24	0.01	0	SRS	1-0	50	-1.99%	1213.32%	1213.32%	1213.32%	1213.32%	1213.32%	1213.32%
24	0.01	0	SRS	1-0,1-3	20	1.77%	1264.48%	1263.37%	4490.82%	1263.37%	4490.82%	1264.48%
24	0.01	0	SRS	1-0,1-3	35	-0.26%	514.66%	513.90%	2182.01%	513.90%	2182.01%	514.66%
24	0.01	0	SRS	1-0,1-3	50	1.41%	305.09%	305.07%	1444.18%	305.07%	1444.18%	305.09%
24	0.01	0	SRS	1-3	20	-0.55%	738.97%	738.97%	3255.87%	738.97%	3255.87%	738.97%
24	0.01	0	SRS	1-3	35	0.58%	387.38%	387.38%	1849.51%	387.38%	1849.51%	387.38%
24	0.01	0	SRS	1-3	50	-0.13%	239.41%	239.41%	1257.63%	239.41%	1257.63%	239.41%
24	0.01	0	StRS	1-0	20	0.28%	3018.99%	3018.99%	3018.99%	3018.99%	3018.99%	3018.99%
24	0.01	0	StRS	1-0	35	0.58%	1757.17%	1757.17%	1757.17%	1757.17%	1757.17%	1757.17%
24	0.01	0	StRS	1-0	50	-0.31%	1197.90%	1197.90%	1197.90%	1197.90%	1197.90%	1197.90%
24	0.01	0	StRS	1-0,1-3	20	0.40%	1192.27%	1192.76%	4216.41%	1192.76%	4216.41%	1192.27%
24	0.01	0	StRS	1-0,1-3	35	0.43%	501.61%	501.64%	2122.22%	501.64%	2122.22%	501.61%
24	0.01	0	StRS	1-0,1-3	50	-0.63%	286.51%	286.69%	1369.59%	286.69%	1369.59%	286.51%
24	0.01	0	StRS	1-3	20	-1.07%	709.25%	709.25%	3137.02%	709.25%	3137.02%	709.25%
24	0.01	0	StRS	1-3	35	0.22%	371.85%	371.85%	1787.38%	371.85%	1787.38%	371.85%
24	0.01	0	StRS	1-3	50	0.23%	231.69%	231.69%	1226.76%	231.69%	1226.76%	231.69%

Table B9: Relative Bias of the ANC Site-to-Site Variance Estimate (shaded cells indicate > 5% or < -5% relative bias)

Years	Trend (p)	Subpop Trend	Sampling Design	Revisit Design	Sites	PO	PWIGLS A-only	PWIGLS A	PWIGLS AI	PWIGLS B	PWIGLS BI	PWIGLS C
24	0.01	0	Unequal	1-0	20	0.24%	2667.16%	2667.16%	2667.16%	2667.16%	2667.16%	2667.16%
24	0.01	0	Unequal	1-0	35	1.83%	1700.87%	1700.87%	1700.87%	1700.87%	1700.87%	1700.87%
24	0.01	0	Unequal	1-0	50	0.23%	1159.35%	1159.35%	1159.35%	1159.35%	1159.35%	1159.35%
24	0.01	0	Unequal	1-0,1-3	20	-1.72%	1131.84%	1131.62%	4023.93%	1131.62%	4023.93%	1131.84%
24	0.01	0	Unequal	1-0,1-3	35	0.30%	486.24%	487.06%	2060.47%	487.06%	2060.47%	486.24%
24	0.01	0	Unequal	1-0,1-3	50	-0.01%	278.71%	279.34%	1337.13%	279.34%	1337.13%	278.71%
24	0.01	0	Unequal	1-3	20	-0.21%	694.17%	694.17%	3076.68%	694.17%	3076.68%	694.17%
24	0.01	0	Unequal	1-3	35	-0.90%	363.42%	363.42%	1753.67%	363.42%	1753.67%	363.42%
24	0.01	0	Unequal	1-3	50	-0.42%	223.40%	223.40%	1193.61%	223.40%	1193.61%	223.40%
24	0.01	0.04	SRS	1-0	20	-1.57%	3102.26%	3102.26%	3102.26%	3102.26%	3102.26%	3102.26%
24	0.01	0.04	SRS	1-0	35	-1.02%	1780.23%	1780.23%	1780.23%	1780.23%	1780.23%	1780.23%
24	0.01	0.04	SRS	1-0	50	-1.22%	1224.51%	1224.51%	1224.51%	1224.51%	1224.51%	1224.51%
24	0.01	0.04	SRS	1-0,1-3	20	0.19%	1243.15%	1243.17%	4432.37%	1243.17%	4432.37%	1243.15%
24	0.01	0.04	SRS	1-0,1-3	35	0.27%	517.74%	517.35%	2192.05%	517.35%	2192.05%	517.74%
24	0.01	0.04	SRS	1-0,1-3	50	-0.39%	298.57%	297.97%	1420.96%	297.97%	1420.96%	298.57%
24	0.01	0.04	SRS	1-3	20	-0.51%	739.73%	739.73%	3258.91%	739.73%	3258.91%	739.73%
24	0.01	0.04	SRS	1-3	35	-0.43%	382.58%	382.58%	1830.32%	382.58%	1830.32%	382.58%
24	0.01	0.04	SRS	1-3	50	-0.04%	239.75%	239.75%	1259.00%	239.75%	1259.00%	239.75%
24	0.01	0.04	StRS	1-0	20	0.26%	3004.47%	3004.47%	3004.47%	3004.47%	3004.47%	3004.47%
24	0.01	0.04	StRS	1-0	35	-0.37%	1735.32%	1735.32%	1735.32%	1735.32%	1735.32%	1735.32%
24	0.01	0.04	StRS	1-0	50	0.95%	1220.64%	1220.64%	1220.64%	1220.64%	1220.64%	1220.64%
24	0.01	0.04	StRS	1-0,1-3	20	1.54%	1217.25%	1216.12%	4286.76%	1216.12%	4286.76%	1217.25%
24	0.01	0.04	StRS	1-0,1-3	35	-0.96%	493.24%	493.69%	2094.28%	493.69%	2094.28%	493.24%
24	0.01	0.04	StRS	1-0,1-3	50	-1.09%	283.70%	284.02%	1356.87%	284.02%	1356.87%	283.70%
24	0.01	0.04	StRS	1-3	20	-0.24%	713.38%	713.38%	3153.53%	713.38%	3153.53%	713.38%
24	0.01	0.04	StRS	1-3	35	-0.54%	366.92%	366.92%	1767.68%	366.92%	1767.68%	366.92%
24	0.01	0.04	StRS	1-3	50	-0.06%	231.76%	231.76%	1227.03%	231.76%	1227.03%	231.76%
24	0.01	0.04	Unequal	1-0	20	-1.21%	2618.63%	2618.63%	2618.63%	2618.63%	2618.63%	2618.63%
24	0.01	0.04	Unequal	1-0	35	0.65%	1642.58%	1642.58%	1642.58%	1642.58%	1642.58%	1642.58%
24	0.01	0.04	Unequal	1-0	50	0.04%	1182.18%	1182.18%	1182.18%	1182.18%	1182.18%	1182.18%
24	0.01	0.04	Unequal	1-0,1-3	20	0.26%	1147.78%	1149.63%	4112.47%	1149.63%	4112.47%	1147.78%
24	0.01	0.04	Unequal	1-0,1-3	35	-0.96%	477.30%	478.98%	2028.42%	478.98%	2028.42%	477.30%
24	0.01	0.04	Unequal	1-0,1-3	50	-0.28%	275.14%	276.19%	1323.14%	276.19%	1323.14%	275.14%
24	0.01	0.04	Unequal	1-3	20	0.05%	682.41%	682.41%	3029.63%	682.41%	3029.63%	682.41%
24	0.01	0.04	Unequal	1-3	35	0.41%	358.37%	358.37%	1733.47%	358.37%	1733.47%	358.37%
24	0.01	0.04	Unequal	1-3	50	-0.41%	217.89%	217.89%	1171.58%	217.89%	1171.58%	217.89%

Table B10: Relative Bias of the modified ANC Site-to-Site Variance Estimate (shaded cells indicate > 5% or < -5% relative bias)

Years	Trend (p)	Subpop Trend	Sampling Design	Revisit Design	Sites	PO	PWIGLS A-only	PWIGLS A	PWIGLS AI	PWIGLS B	PWIGLS BI	PWIGLS C
12	0.02	0	SRS	1-0	20	3.32%	3245.80%	3245.80%	3245.80%	3245.80%	3245.80%	3245.80%
12	0.02	0	SRS	1-0	35	-0.11%	1790.38%	1790.38%	1790.38%	1790.38%	1790.38%	1790.38%
12	0.02	0	SRS	1-0	50	0.17%	1238.79%	1238.79%	1238.79%	1238.79%	1238.79%	1238.79%
12	0.02	0	SRS	1-0,1-3	20	-1.22%	1219.88%	1219.61%	4320.80%	1219.61%	4320.80%	1219.88%
12	0.02	0	SRS	1-0,1-3	35	-1.30%	506.35%	506.22%	2143.62%	506.22%	2143.62%	506.35%
12	0.02	0	SRS	1-0,1-3	50	-0.35%	297.42%	297.36%	1411.97%	297.36%	1411.97%	297.42%
12	0.02	0	SRS	1-3	20	0.42%	745.05%	745.06%	3280.22%	745.06%	3280.22%	745.06%
12	0.02	0	SRS	1-3	35	0.75%	387.20%	387.19%	1848.78%	387.19%	1848.78%	387.19%
12	0.02	0	SRS	1-3	50	-0.15%	238.74%	238.74%	1254.94%	238.74%	1254.94%	238.74%
12	0.02	0	StRS	1-0	20	0.93%	3075.73%	3075.73%	3075.73%	3075.73%	3075.73%	3075.73%
12	0.02	0	StRS	1-0	35	-0.62%	1725.98%	1725.98%	1725.98%	1725.98%	1725.98%	1725.98%
12	0.02	0	StRS	1-0	50	-1.24%	1195.64%	1195.64%	1195.64%	1195.64%	1195.64%	1195.64%
12	0.02	0	StRS	1-0,1-3	20	0.98%	1210.19%	1210.11%	4272.16%	1210.11%	4272.16%	1210.19%
12	0.02	0	StRS	1-0,1-3	35	0.82%	507.45%	507.39%	2150.86%	507.39%	2150.86%	507.45%
12	0.02	0	StRS	1-0,1-3	50	1.33%	298.05%	298.03%	1417.21%	298.03%	1417.21%	298.05%
12	0.02	0	StRS	1-3	20	-0.45%	717.60%	717.60%	3170.39%	717.60%	3170.39%	717.60%
12	0.02	0	StRS	1-3	35	0.22%	374.58%	374.58%	1798.32%	374.58%	1798.32%	374.58%
12	0.02	0	StRS	1-3	50	0.38%	235.52%	235.52%	1242.07%	235.52%	1242.07%	235.52%
12	0.02	0	Unequal	1-0	20	-1.20%	2665.98%	2665.98%	2665.98%	2665.98%	2665.98%	2665.98%
12	0.02	0	Unequal	1-0	35	0.58%	1702.09%	1702.09%	1702.09%	1702.09%	1702.09%	1702.09%
12	0.02	0	Unequal	1-0	50	0.12%	1183.63%	1183.63%	1183.63%	1183.63%	1183.63%	1183.63%
12	0.02	0	Unequal	1-0,1-3	20	0.22%	1143.10%	1142.87%	4049.80%	1142.87%	4049.80%	1143.10%
12	0.02	0	Unequal	1-0,1-3	35	0.50%	488.33%	488.31%	2065.41%	488.31%	2065.41%	488.33%
12	0.02	0	Unequal	1-0,1-3	50	-0.01%	279.13%	279.10%	1337.20%	279.10%	1337.20%	279.13%
12	0.02	0	Unequal	1-3	20	-1.12%	688.03%	688.02%	3052.10%	688.02%	3052.10%	688.02%
12	0.02	0	Unequal	1-3	35	-0.72%	360.67%	360.68%	1742.71%	360.68%	1742.71%	360.68%
12	0.02	0	Unequal	1-3	50	-0.44%	223.71%	223.71%	1194.83%	223.71%	1194.83%	223.71%
12	0.02	0.04	SRS	1-0	20	-0.72%	3115.03%	3115.03%	3115.03%	3115.03%	3115.03%	3115.03%
12	0.02	0.04	SRS	1-0	35	-1.33%	1767.30%	1767.30%	1767.30%	1767.30%	1767.30%	1767.30%
12	0.02	0.04	SRS	1-0	50	-1.19%	1220.62%	1220.62%	1220.62%	1220.62%	1220.62%	1220.62%
12	0.02	0.04	SRS	1-0,1-3	20	-0.19%	1233.80%	1233.37%	4334.70%	1233.37%	4334.70%	1233.80%
12	0.02	0.04	SRS	1-0,1-3	35	0.16%	515.26%	515.18%	2176.68%	515.18%	2176.68%	515.27%
12	0.02	0.04	SRS	1-0,1-3	50	0.71%	301.65%	301.57%	1425.54%	301.57%	1425.54%	301.65%
12	0.02	0.04	SRS	1-3	20	0.66%	747.01%	747.01%	3288.05%	747.01%	3288.05%	747.01%
12	0.02	0.04	SRS	1-3	35	-0.17%	382.75%	382.75%	1831.02%	382.75%	1831.02%	382.75%
12	0.02	0.04	SRS	1-3	50	-1.00%	235.86%	235.86%	1243.44%	235.86%	1243.44%	235.86%

Table B10: Relative Bias of the modified ANC Site-to-Site Variance Estimate (shaded cells indicate > 5% or < -5% relative bias)

Years	Trend (p)	Subpop Trend	Sampling Design	Revisit Design	Sites	PO	PWIGLS A-only	PWIGLS A	PWIGLS AI	PWIGLS B	PWIGLS BI	PWIGLS C
12	0.02	0.04	StRS	1-0	20	-0.38%	2955.34%	2955.34%	2955.34%	2955.34%	2955.34%	2955.34%
12	0.02	0.04	StRS	1-0	35	-0.64%	1748.33%	1748.33%	1748.33%	1748.33%	1748.33%	1748.33%
12	0.02	0.04	StRS	1-0	50	0.46%	1203.34%	1203.34%	1203.34%	1203.34%	1203.34%	1203.34%
12	0.02	0.04	StRS	1-0,1-3	20	-0.53%	1186.19%	1185.96%	4225.71%	1185.96%	4225.71%	1186.19%
12	0.02	0.04	StRS	1-0,1-3	35	-1.01%	496.21%	496.14%	2112.21%	496.14%	2112.21%	496.21%
12	0.02	0.04	StRS	1-0,1-3	50	-0.35%	291.37%	291.35%	1392.15%	291.35%	1392.15%	291.37%
12	0.02	0.04	StRS	1-3	20	0.16%	724.53%	724.53%	3198.12%	724.53%	3198.12%	724.53%
12	0.02	0.04	StRS	1-3	35	-0.43%	370.37%	370.37%	1781.48%	370.37%	1781.48%	370.37%
12	0.02	0.04	StRS	1-3	50	0.61%	234.99%	234.99%	1239.97%	234.99%	1239.97%	234.99%
12	0.02	0.04	Unequal	1-0	20	-0.96%	2589.44%	2589.44%	2589.44%	2589.44%	2589.44%	2589.44%
12	0.02	0.04	Unequal	1-0	35	1.22%	1665.04%	1665.04%	1665.04%	1665.04%	1665.04%	1665.04%
12	0.02	0.04	Unequal	1-0	50	0.84%	1196.51%	1196.51%	1196.51%	1196.51%	1196.51%	1196.51%
12	0.02	0.04	Unequal	1-0,1-3	20	0.50%	1124.88%	1124.67%	3931.33%	1124.67%	3931.33%	1124.88%
12	0.02	0.04	Unequal	1-0,1-3	35	-0.62%	476.34%	476.25%	2019.31%	476.25%	2019.31%	476.34%
12	0.02	0.04	Unequal	1-0,1-3	50	-0.36%	278.81%	278.78%	1328.79%	278.78%	1328.79%	278.81%
12	0.02	0.04	Unequal	1-3	20	1.13%	698.97%	698.96%	3095.86%	698.96%	3095.86%	698.96%
12	0.02	0.04	Unequal	1-3	35	1.14%	359.40%	359.40%	1737.62%	359.40%	1737.62%	359.40%
12	0.02	0.04	Unequal	1-3	50	0.06%	216.48%	216.48%	1165.88%	216.48%	1165.88%	216.48%
24	0.01	0	SRS	1-0	20	-0.02%	3138.19%	3138.19%	3138.19%	3138.19%	3138.19%	3138.19%
24	0.01	0	SRS	1-0	35	1.57%	1822.50%	1822.50%	1822.50%	1822.50%	1822.50%	1822.50%
24	0.01	0	SRS	1-0	50	0.23%	1239.66%	1239.66%	1239.66%	1239.66%	1239.66%	1239.66%
24	0.01	0	SRS	1-0,1-3	20	1.09%	1251.15%	1250.96%	4445.86%	1250.96%	4445.86%	1251.15%
24	0.01	0	SRS	1-0,1-3	35	0.91%	519.87%	519.84%	2199.77%	519.84%	2199.77%	519.87%
24	0.01	0	SRS	1-0,1-3	50	-0.03%	298.66%	298.65%	1419.27%	298.65%	1419.27%	298.66%
24	0.01	0	SRS	1-3	20	0.49%	745.82%	745.82%	3283.29%	745.82%	3283.29%	745.82%
24	0.01	0	SRS	1-3	35	0.00%	383.60%	383.60%	1834.40%	383.60%	1834.40%	383.60%
24	0.01	0	SRS	1-3	50	0.10%	239.63%	239.63%	1258.51%	239.63%	1258.51%	239.63%
24	0.01	0	StRS	1-0	20	-0.37%	3005.39%	3005.39%	3005.39%	3005.39%	3005.39%	3005.39%
24	0.01	0	StRS	1-0	35	0.62%	1763.58%	1763.58%	1763.58%	1763.58%	1763.58%	1763.58%
24	0.01	0	StRS	1-0	50	0.39%	1218.23%	1218.23%	1218.23%	1218.23%	1218.23%	1218.23%
24	0.01	0	StRS	1-0,1-3	20	-1.31%	1181.25%	1181.18%	4202.41%	1181.18%	4202.41%	1181.25%
24	0.01	0	StRS	1-0,1-3	35	-0.08%	503.63%	503.64%	2135.89%	503.64%	2135.89%	503.63%
24	0.01	0	StRS	1-0,1-3	50	-0.66%	288.43%	288.42%	1378.24%	288.42%	1378.24%	288.43%
24	0.01	0	StRS	1-3	20	-1.47%	706.07%	706.07%	3124.30%	706.07%	3124.30%	706.07%
24	0.01	0	StRS	1-3	35	0.06%	374.49%	374.49%	1797.95%	374.49%	1797.95%	374.49%
24	0.01	0	StRS	1-3	50	0.01%	233.43%	233.43%	1233.72%	233.43%	1233.72%	233.43%

Table B10: Relative Bias of the modified ANC Site-to-Site Variance Estimate (shaded cells indicate > 5% or < -5% relative bias)

Years	Trend (p)	Subpop Trend	Sampling Design	Revisit Design	Sites	PO	PWIGLS A-only	PWIGLS A	PWIGLS AI	PWIGLS B	PWIGLS BI	PWIGLS C
24	0.01	0	Unequal	1-0	20	2.64%	2710.79%	2710.79%	2710.79%	2710.79%	2710.79%	2710.79%
24	0.01	0	Unequal	1-0	35	-0.14%	1698.63%	1698.63%	1698.63%	1698.63%	1698.63%	1698.63%
24	0.01	0	Unequal	1-0	50	1.09%	1209.92%	1209.92%	1209.92%	1209.92%	1209.92%	1209.92%
24	0.01	0	Unequal	1-0,1-3	20	0.55%	1122.02%	1121.97%	3965.83%	1121.97%	3965.83%	1122.02%
24	0.01	0	Unequal	1-0,1-3	35	0.48%	495.84%	495.84%	2102.92%	495.84%	2102.92%	495.84%
24	0.01	0	Unequal	1-0,1-3	50	0.43%	287.09%	287.08%	1372.70%	287.08%	1372.70%	287.09%
24	0.01	0	Unequal	1-3	20	-0.02%	700.16%	700.16%	3100.62%	700.16%	3100.62%	700.16%
24	0.01	0	Unequal	1-3	35	-0.76%	360.51%	360.51%	1742.05%	360.51%	1742.05%	360.51%
24	0.01	0	Unequal	1-3	50	-0.46%	226.35%	226.35%	1205.40%	226.35%	1205.40%	226.35%
24	0.01	0.04	SRS	1-0	20	-0.34%	3127.86%	3127.86%	3127.86%	3127.86%	3127.86%	3127.86%
24	0.01	0.04	SRS	1-0	35	-1.21%	1769.74%	1769.74%	1769.74%	1769.74%	1769.74%	1769.74%
24	0.01	0.04	SRS	1-0	50	-1.17%	1221.06%	1221.06%	1221.06%	1221.06%	1221.06%	1221.06%
24	0.01	0.04	SRS	1-0,1-3	20	0.03%	1236.81%	1236.76%	4389.18%	1236.76%	4389.18%	1236.81%
24	0.01	0.04	SRS	1-0,1-3	35	0.55%	517.72%	517.69%	2195.18%	517.69%	2195.18%	517.72%
24	0.01	0.04	SRS	1-0,1-3	50	-0.73%	295.90%	295.88%	1409.05%	295.88%	1409.05%	295.90%
24	0.01	0.04	SRS	1-3	20	0.34%	744.52%	744.52%	3278.07%	744.52%	3278.07%	744.52%
24	0.01	0.04	SRS	1-3	35	0.48%	385.94%	385.94%	1843.75%	385.94%	1843.75%	385.94%
24	0.01	0.04	SRS	1-3	50	-0.03%	239.17%	239.17%	1256.70%	239.17%	1256.70%	239.17%
24	0.01	0.04	StRS	1-0	20	-1.11%	2960.40%	2960.40%	2960.40%	2960.40%	2960.40%	2960.40%
24	0.01	0.04	StRS	1-0	35	1.04%	1750.70%	1750.70%	1750.70%	1750.70%	1750.70%	1750.70%
24	0.01	0.04	StRS	1-0	50	-1.02%	1196.44%	1196.44%	1196.44%	1196.44%	1196.44%	1196.44%
24	0.01	0.04	StRS	1-0,1-3	20	-1.82%	1183.06%	1183.08%	4202.93%	1183.08%	4202.93%	1183.06%
24	0.01	0.04	StRS	1-0,1-3	35	-0.32%	500.68%	500.67%	2124.76%	500.67%	2124.76%	500.68%
24	0.01	0.04	StRS	1-0,1-3	50	0.37%	294.64%	294.63%	1402.71%	294.63%	1402.71%	294.64%
24	0.01	0.04	StRS	1-3	20	-0.32%	722.66%	722.66%	3190.65%	722.66%	3190.65%	722.66%
24	0.01	0.04	StRS	1-3	35	-0.77%	369.73%	369.73%	1778.93%	369.73%	1778.93%	369.73%
24	0.01	0.04	StRS	1-3	50	-0.60%	231.09%	231.09%	1224.34%	231.09%	1224.34%	231.09%
24	0.01	0.04	Unequal	1-0	20	-0.72%	2540.62%	2540.62%	2540.62%	2540.62%	2540.62%	2540.62%
24	0.01	0.04	Unequal	1-0	35	-3.02%	1622.39%	1622.39%	1622.39%	1622.39%	1622.39%	1622.39%
24	0.01	0.04	Unequal	1-0	50	-0.20%	1158.01%	1158.01%	1158.01%	1158.01%	1158.01%	1158.01%
24	0.01	0.04	Unequal	1-0,1-3	20	-0.62%	1120.21%	1120.15%	3943.93%	1120.15%	3943.93%	1120.21%
24	0.01	0.04	Unequal	1-0,1-3	35	0.54%	490.30%	490.30%	2078.87%	490.30%	2078.87%	490.30%
24	0.01	0.04	Unequal	1-0,1-3	50	0.32%	278.11%	278.10%	1332.73%	278.10%	1332.73%	278.11%
24	0.01	0.04	Unequal	1-3	20	-0.05%	688.73%	688.73%	3054.93%	688.73%	3054.93%	688.73%
24	0.01	0.04	Unequal	1-3	35	0.50%	358.64%	358.64%	1734.55%	358.64%	1734.55%	358.64%
24	0.01	0.04	Unequal	1-3	50	0.20%	222.40%	222.40%	1189.59%	222.40%	1189.59%	222.40%

Table B11: Relative Bias of the ANC Site-Level Slope Variance Estimate (shaded cells indicate > 5% or < -5% relative bias)

Years	Trend (p)	Subpop Trend	Sampling Design	Revisit Design	Sites	PO	PWIGLS A-only	PWIGLS A	PWIGLS AI	PWIGLS B	PWIGLS BI	PWIGLS C
12	0.02	0	SRS	1-0	20	-2.66%	3170.55%	3170.55%	3170.55%	3170.55%	3170.55%	3170.55%
12	0.02	0	SRS	1-0	35	-0.06%	1837.79%	1837.79%	1837.79%	1837.79%	1837.79%	1837.79%
12	0.02	0	SRS	1-0	50	3.53%	1308.38%	1308.38%	1308.38%	1308.38%	1308.38%	1308.38%
12	0.02	0	SRS	1-0,1-3	20	-0.13%	1437.72%	1251.79%	1944.90%	1251.79%	1944.90%	1437.72%
12	0.02	0	SRS	1-0,1-3	35	-2.27%	491.53%	504.50%	811.57%	504.50%	811.57%	491.53%
12	0.02	0	SRS	1-0,1-3	50	-1.92%	242.38%	293.00%	492.16%	293.00%	492.16%	242.39%
12	0.02	0	SRS	1-3	20	2.54%	771.24%	771.17%	3384.97%	771.17%	3384.97%	771.17%
12	0.02	0	SRS	1-3	35	3.27%	402.34%	402.39%	1909.55%	402.39%	1909.55%	402.39%
12	0.02	0	SRS	1-3	50	2.26%	248.43%	248.45%	1293.78%	248.45%	1293.78%	248.45%
12	0.02	0	StRS	1-0	20	6.22%	1506.31%	1506.31%	1506.31%	1506.31%	1506.31%	1506.31%
12	0.02	0	StRS	1-0	35	2.70%	521.88%	521.88%	521.88%	521.88%	521.88%	521.88%
12	0.02	0	StRS	1-0	50	0.37%	259.66%	259.66%	259.66%	259.66%	259.66%	259.66%
12	0.02	0	StRS	1-0,1-3	20	12.48%	621.04%	440.42%	1316.58%	440.42%	1316.58%	621.04%
12	0.02	0	StRS	1-0,1-3	35	20.00%	172.54%	115.71%	488.75%	115.71%	488.75%	172.54%
12	0.02	0	StRS	1-0,1-3	50	21.78%	41.38%	23.67%	252.29%	23.67%	252.29%	41.38%
12	0.02	0	StRS	1-3	20	69.48%	455.73%	452.25%	2122.97%	452.25%	2122.97%	452.25%
12	0.02	0	StRS	1-3	35	58.01%	151.84%	151.86%	907.46%	151.86%	907.46%	151.86%
12	0.02	0	StRS	1-3	50	55.88%	70.21%	70.21%	580.84%	70.21%	580.84%	70.21%
12	0.02	0	Unequal	1-0	20	-2.15%	1479.20%	1479.20%	1479.20%	1479.20%	1479.20%	1479.20%
12	0.02	0	Unequal	1-0	35	1.31%	860.68%	860.68%	860.68%	860.68%	860.68%	860.68%
12	0.02	0	Unequal	1-0	50	-0.26%	522.27%	522.27%	522.27%	522.27%	522.27%	522.27%
12	0.02	0	Unequal	1-0,1-3	20	2.48%	545.53%	477.97%	946.99%	477.97%	946.99%	545.53%
12	0.02	0	Unequal	1-0,1-3	35	-0.07%	105.52%	128.28%	331.08%	128.28%	331.08%	105.52%
12	0.02	0	Unequal	1-0,1-3	50	1.01%	6.02%	35.15%	143.31%	35.15%	143.31%	6.02%
12	0.02	0	Unequal	1-3	20	3.89%	186.90%	186.88%	1047.60%	186.88%	1047.60%	186.88%
12	0.02	0	Unequal	1-3	35	-1.42%	33.71%	33.79%	435.11%	33.79%	435.11%	33.79%
12	0.02	0	Unequal	1-3	50	4.46%	-8.31%	-8.31%	266.76%	-8.31%	266.76%	-8.31%
12	0.02	0.04	SRS	1-0	20	162.94%	8721.32%	8721.32%	8721.32%	8721.32%	8721.32%	8721.32%
12	0.02	0.04	SRS	1-0	35	156.53%	4869.40%	4869.40%	4869.40%	4869.40%	4869.40%	4869.40%
12	0.02	0.04	SRS	1-0	50	162.64%	3471.26%	3471.26%	3471.26%	3471.26%	3471.26%	3471.26%
12	0.02	0.04	SRS	1-0,1-3	20	160.37%	3791.97%	3430.08%	7435.53%	3430.08%	7435.53%	3791.97%
12	0.02	0.04	SRS	1-0,1-3	35	165.88%	1587.86%	1545.58%	4067.94%	1545.58%	4067.94%	1587.86%
12	0.02	0.04	SRS	1-0,1-3	50	160.51%	912.81%	944.23%	2694.55%	944.23%	2694.55%	912.81%
12	0.02	0.04	SRS	1-3	20	165.18%	2155.14%	2155.21%	8920.82%	2155.21%	8920.82%	2155.21%
12	0.02	0.04	SRS	1-3	35	160.96%	1170.13%	1170.13%	4980.52%	1170.13%	4980.52%	1170.13%
12	0.02	0.04	SRS	1-3	50	159.48%	784.42%	784.42%	3437.70%	784.42%	3437.70%	784.42%

Table B11: Relative Bias of the ANC Site-Level Slope Variance Estimate (shaded cells indicate > 5% or < -5% relative bias)

Years	Trend (p)	Subpop Trend	Sampling Design	Revisit Design	Sites	PO	PWIGLS A-only	PWIGLS A	PWIGLS AI	PWIGLS B	PWIGLS BI	PWIGLS C
12	0.02	0.04	StRS	1-0	20	-45.87%	837.36%	837.36%	837.36%	837.36%	837.36%	837.36%
12	0.02	0.04	StRS	1-0	35	-51.48%	370.83%	370.83%	370.83%	370.83%	370.83%	370.83%
12	0.02	0.04	StRS	1-0	50	-52.29%	192.57%	192.57%	192.57%	192.57%	192.57%	192.57%
12	0.02	0.04	StRS	1-0,1-3	20	-32.74%	338.87%	327.56%	873.81%	327.56%	873.81%	340.76%
12	0.02	0.04	StRS	1-0,1-3	35	-25.30%	55.65%	67.86%	256.25%	67.86%	256.25%	55.65%
12	0.02	0.04	StRS	1-0,1-3	50	-17.82%	-1.61%	20.24%	136.46%	20.24%	136.46%	-1.62%
12	0.02	0.04	StRS	1-3	20	16.72%	348.12%	348.03%	1692.13%	348.03%	1692.13%	348.03%
12	0.02	0.04	StRS	1-3	35	10.40%	127.91%	127.92%	811.42%	127.92%	811.42%	127.92%
12	0.02	0.04	StRS	1-3	50	4.85%	46.45%	46.45%	485.81%	46.45%	485.81%	46.45%
12	0.02	0.04	Unequal	1-0	20	265.93%	6305.68%	6305.68%	6305.68%	6305.68%	6305.68%	6305.68%
12	0.02	0.04	Unequal	1-0	35	262.62%	3694.81%	3694.81%	3694.81%	3694.81%	3694.81%	3694.81%
12	0.02	0.04	Unequal	1-0	50	260.56%	2429.94%	2429.94%	2429.94%	2429.94%	2429.94%	2429.94%
12	0.02	0.04	Unequal	1-0,1-3	20	254.84%	2488.27%	2233.96%	5507.71%	2233.96%	5507.71%	2488.27%
12	0.02	0.04	Unequal	1-0,1-3	35	261.71%	1010.33%	956.05%	2892.25%	956.05%	2892.25%	1010.33%
12	0.02	0.04	Unequal	1-0,1-3	50	260.89%	544.98%	547.99%	1832.84%	547.99%	1832.84%	544.98%
12	0.02	0.04	Unequal	1-3	20	267.80%	1273.43%	1273.43%	5393.74%	1273.43%	5393.74%	1273.43%
12	0.02	0.04	Unequal	1-3	35	259.00%	641.47%	641.47%	2865.89%	641.47%	2865.89%	641.47%
12	0.02	0.04	Unequal	1-3	50	262.88%	427.07%	427.07%	2008.27%	427.07%	2008.27%	427.07%
24	0.01	0	SRS	1-0	20	1.46%	3286.16%	3286.16%	3286.16%	3286.16%	3286.16%	3286.16%
24	0.01	0	SRS	1-0	35	0.48%	1840.97%	1840.97%	1840.97%	1840.97%	1840.97%	1840.97%
24	0.01	0	SRS	1-0	50	0.47%	1263.65%	1263.65%	1263.65%	1263.65%	1263.65%	1263.65%
24	0.01	0	SRS	1-0,1-3	20	2.23%	1307.66%	1285.57%	4052.02%	1285.57%	4052.02%	1307.66%
24	0.01	0	SRS	1-0,1-3	35	0.01%	523.42%	518.98%	2068.71%	518.98%	2068.71%	523.42%
24	0.01	0	SRS	1-0,1-3	50	0.40%	303.25%	302.42%	1377.36%	302.42%	1377.36%	303.25%
24	0.01	0	SRS	1-3	20	-0.72%	743.98%	743.98%	3275.92%	743.98%	3275.92%	743.98%
24	0.01	0	SRS	1-3	35	0.67%	389.88%	389.88%	1859.52%	389.88%	1859.52%	389.88%
24	0.01	0	SRS	1-3	50	0.08%	241.07%	241.07%	1264.29%	241.07%	1264.29%	241.07%
24	0.01	0	StRS	1-0	20	-1.76%	2611.96%	2611.96%	2611.96%	2611.96%	2611.96%	2611.96%
24	0.01	0	StRS	1-0	35	-1.68%	1464.51%	1464.51%	1464.51%	1464.51%	1464.51%	1464.51%
24	0.01	0	StRS	1-0	50	-2.77%	993.59%	993.59%	993.59%	993.59%	993.59%	993.59%
24	0.01	0	StRS	1-0,1-3	20	1.28%	888.56%	868.96%	2303.97%	868.96%	2303.97%	888.56%
24	0.01	0	StRS	1-0,1-3	35	-1.99%	310.64%	301.61%	1149.55%	301.61%	1149.55%	310.64%
24	0.01	0	StRS	1-0,1-3	50	-1.67%	158.58%	154.11%	756.46%	154.11%	756.46%	158.58%
24	0.01	0	StRS	1-3	20	-2.65%	430.72%	430.72%	2022.88%	430.72%	2022.88%	430.72%
24	0.01	0	StRS	1-3	35	-2.29%	199.07%	199.07%	1096.27%	199.07%	1096.27%	199.07%
24	0.01	0	StRS	1-3	50	-0.47%	113.06%	113.06%	752.25%	113.06%	752.25%	113.06%

Table B11: Relative Bias of the ANC Site-Level Slope Variance Estimate (shaded cells indicate > 5% or < -5% relative bias)

Years	Trend (p)	Subpop Trend	Sampling Design	Revisit Design	Sites	PO	PWIGLS A-only	PWIGLS A	PWIGLS AI	PWIGLS B	PWIGLS BI	PWIGLS C
24	0.01	0	Unequal	1-0	20	0.89%	2238.31%	2238.31%	2238.31%	2238.31%	2238.31%	2238.31%
24	0.01	0	Unequal	1-0	35	-0.23%	1334.46%	1334.46%	1334.46%	1334.46%	1334.46%	1334.46%
24	0.01	0	Unequal	1-0	50	-0.04%	876.29%	876.29%	876.29%	876.29%	876.29%	876.29%
24	0.01	0	Unequal	1-0,1-3	20	0.12%	750.41%	728.10%	2249.87%	728.10%	2249.87%	750.41%
24	0.01	0	Unequal	1-0,1-3	35	-0.05%	276.55%	274.74%	1141.24%	274.74%	1141.24%	276.55%
24	0.01	0	Unequal	1-0,1-3	50	0.85%	132.81%	133.38%	721.19%	133.38%	721.19%	132.81%
24	0.01	0	Unequal	1-3	20	0.77%	394.69%	394.69%	1878.76%	394.69%	1878.76%	394.69%
24	0.01	0	Unequal	1-3	35	-0.47%	173.76%	173.76%	995.05%	173.76%	995.05%	173.76%
24	0.01	0	Unequal	1-3	50	-0.77%	89.23%	89.23%	656.92%	89.23%	656.92%	89.23%
24	0.01	0.04	SRS	1-0	20	164.20%	8635.02%	8635.02%	8635.02%	8635.02%	8635.02%	8635.02%
24	0.01	0.04	SRS	1-0	35	169.85%	5085.47%	5085.47%	5085.47%	5085.47%	5085.47%	5085.47%
24	0.01	0.04	SRS	1-0	50	164.00%	3472.11%	3472.11%	3472.11%	3472.11%	3472.11%	3472.11%
24	0.01	0.04	SRS	1-0,1-3	20	161.98%	3466.68%	3442.30%	11319.55%	3442.30%	11319.55%	3466.68%
24	0.01	0.04	SRS	1-0,1-3	35	161.55%	1529.37%	1517.40%	5827.58%	1517.40%	5827.58%	1529.37%
24	0.01	0.04	SRS	1-0,1-3	50	163.76%	965.10%	956.71%	3909.35%	956.71%	3909.35%	965.10%
24	0.01	0.04	SRS	1-3	20	166.60%	2163.38%	2163.38%	8953.50%	2163.38%	8953.50%	2163.38%
24	0.01	0.04	SRS	1-3	35	162.83%	1178.18%	1178.18%	5012.71%	1178.18%	5012.71%	1178.18%
24	0.01	0.04	SRS	1-3	50	162.11%	792.92%	792.92%	3471.69%	792.92%	3471.69%	792.92%
24	0.01	0.04	StRS	1-0	20	-8.53%	2438.88%	2438.88%	2438.88%	2438.88%	2438.88%	2438.88%
24	0.01	0.04	StRS	1-0	35	-6.01%	1447.67%	1447.67%	1447.67%	1447.67%	1447.67%	1447.67%
24	0.01	0.04	StRS	1-0	50	-6.76%	988.87%	988.87%	988.87%	988.87%	988.87%	988.87%
24	0.01	0.04	StRS	1-0,1-3	20	-7.56%	784.17%	779.01%	1980.02%	779.01%	1980.02%	784.17%
24	0.01	0.04	StRS	1-0,1-3	35	-5.34%	295.50%	298.19%	1091.62%	298.19%	1091.62%	295.50%
24	0.01	0.04	StRS	1-0,1-3	50	-5.49%	149.75%	153.13%	726.44%	153.13%	726.44%	149.75%
24	0.01	0.04	StRS	1-3	20	-9.43%	381.77%	381.77%	1827.09%	381.77%	1827.09%	381.77%
24	0.01	0.04	StRS	1-3	35	-7.57%	185.76%	185.76%	1043.03%	185.76%	1043.03%	185.76%
24	0.01	0.04	StRS	1-3	50	-8.01%	99.71%	99.71%	698.85%	99.71%	698.85%	99.71%
24	0.01	0.04	Unequal	1-0	20	285.59%	8061.56%	8061.56%	8061.56%	8061.56%	8061.56%	8061.56%
24	0.01	0.04	Unequal	1-0	35	272.86%	4551.68%	4551.68%	4551.68%	4551.68%	4551.68%	4551.68%
24	0.01	0.04	Unequal	1-0	50	270.56%	3151.74%	3151.74%	3151.74%	3151.74%	3151.74%	3151.74%
24	0.01	0.04	Unequal	1-0,1-3	20	265.06%	2909.82%	2852.55%	9510.73%	2852.55%	9510.73%	2909.82%
24	0.01	0.04	Unequal	1-0,1-3	35	263.15%	1283.77%	1257.77%	4891.50%	1257.77%	4891.50%	1283.77%
24	0.01	0.04	Unequal	1-0,1-3	50	266.17%	783.12%	765.38%	3197.97%	765.38%	3197.97%	783.12%
24	0.01	0.04	Unequal	1-3	20	263.87%	1772.32%	1772.32%	7389.29%	1772.32%	7389.29%	1772.32%
24	0.01	0.04	Unequal	1-3	35	267.48%	956.96%	956.96%	4127.83%	956.96%	4127.83%	956.96%
24	0.01	0.04	Unequal	1-3	50	265.89%	632.82%	632.82%	2831.26%	632.82%	2831.26%	632.82%

Table B12: Relative Bias of the modified ANC Site-Level Slope Variance Estimate (shaded cells indicate > 5% or < -5% relative bias)

Years	Trend (p)	Subpop Trend	Sampling Design	Revisit Design	Sites	PO	PWIGLS A-only	PWIGLS A	PWIGLS AI	PWIGLS B	PWIGLS BI	PWIGLS C
12	0.02	0	SRS	1-0	20	-2.26%	2970.59%	2970.59%	2970.59%	2970.59%	2970.59%	2970.59%
12	0.02	0	SRS	1-0	35	-0.24%	1758.54%	1758.54%	1758.54%	1758.54%	1758.54%	1758.54%
12	0.02	0	SRS	1-0	50	-0.08%	1222.20%	1222.20%	1222.20%	1222.20%	1222.20%	1222.20%
12	0.02	0	SRS	1-0,1-3	20	5.44%	1264.40%	1251.81%	1867.95%	1251.81%	1867.95%	1264.40%
12	0.02	0	SRS	1-0,1-3	35	-1.38%	498.32%	491.99%	922.52%	491.99%	922.52%	498.32%
12	0.02	0	SRS	1-0,1-3	50	-4.54%	278.06%	274.90%	606.30%	274.90%	606.30%	278.13%
12	0.02	0	SRS	1-3	20	4.93%	759.43%	759.56%	3338.20%	759.56%	3338.20%	759.56%
12	0.02	0	SRS	1-3	35	1.02%	380.91%	380.83%	1823.74%	380.83%	1823.74%	380.83%
12	0.02	0	SRS	1-3	50	-0.48%	233.51%	233.49%	1234.14%	233.49%	1234.14%	233.49%
12	0.02	0	StRS	1-0	20	0.50%	2089.29%	2089.29%	2089.29%	2089.29%	2089.29%	2089.29%
12	0.02	0	StRS	1-0	35	-4.30%	1051.15%	1051.15%	1051.15%	1051.15%	1051.15%	1051.15%
12	0.02	0	StRS	1-0	50	0.79%	771.10%	771.10%	771.10%	771.10%	771.10%	771.10%
12	0.02	0	StRS	1-0,1-3	20	-0.27%	627.27%	620.90%	1260.53%	620.90%	1260.53%	627.28%
12	0.02	0	StRS	1-0,1-3	35	-2.91%	180.42%	177.94%	511.85%	177.94%	511.85%	180.45%
12	0.02	0	StRS	1-0,1-3	50	0.43%	76.65%	75.35%	334.11%	75.35%	334.11%	76.66%
12	0.02	0	StRS	1-3	20	3.51%	257.39%	257.42%	1329.68%	257.42%	1329.68%	257.42%
12	0.02	0	StRS	1-3	35	2.49%	82.23%	82.25%	629.09%	82.25%	629.09%	82.25%
12	0.02	0	StRS	1-3	50	0.10%	22.53%	22.53%	390.15%	22.53%	390.15%	22.53%
12	0.02	0	Unequal	1-0	20	1.79%	1728.33%	1728.33%	1728.33%	1728.33%	1728.33%	1728.33%
12	0.02	0	Unequal	1-0	35	2.61%	986.32%	986.32%	986.32%	986.32%	986.32%	986.32%
12	0.02	0	Unequal	1-0	50	-2.49%	592.95%	592.95%	592.95%	592.95%	592.95%	592.95%
12	0.02	0	Unequal	1-0,1-3	20	-1.02%	564.41%	557.84%	1089.71%	557.84%	1089.71%	564.41%
12	0.02	0	Unequal	1-0,1-3	35	2.06%	191.55%	188.47%	516.39%	188.47%	516.39%	191.55%
12	0.02	0	Unequal	1-0,1-3	50	1.34%	75.54%	74.36%	287.43%	74.36%	287.43%	75.56%
12	0.02	0	Unequal	1-3	20	0.03%	257.01%	257.12%	1328.35%	257.12%	1328.35%	257.12%
12	0.02	0	Unequal	1-3	35	3.23%	91.03%	91.02%	664.17%	91.02%	664.17%	91.02%
12	0.02	0	Unequal	1-3	50	1.63%	33.15%	33.13%	432.50%	33.13%	432.50%	33.13%
12	0.02	0.04	SRS	1-0	20	158.55%	8179.37%	8179.37%	8179.37%	8179.37%	8179.37%	8179.37%
12	0.02	0.04	SRS	1-0	35	154.32%	4686.82%	4686.82%	4686.82%	4686.82%	4686.82%	4686.82%
12	0.02	0.04	SRS	1-0	50	159.33%	3354.00%	3354.00%	3354.00%	3354.00%	3354.00%	3354.00%
12	0.02	0.04	SRS	1-0,1-3	20	168.42%	3443.07%	3425.76%	6695.39%	3425.76%	6695.39%	3443.07%
12	0.02	0.04	SRS	1-0,1-3	35	162.11%	1503.85%	1497.13%	4086.63%	1497.13%	4086.63%	1503.93%
12	0.02	0.04	SRS	1-0,1-3	50	166.44%	960.80%	957.19%	3109.16%	957.19%	3109.16%	960.91%
12	0.02	0.04	SRS	1-3	20	159.87%	2060.71%	2060.76%	8543.06%	2060.76%	8543.06%	2060.76%
12	0.02	0.04	SRS	1-3	35	164.96%	1173.17%	1173.25%	4993.04%	1173.25%	4993.04%	1173.25%
12	0.02	0.04	SRS	1-3	50	157.30%	769.18%	769.20%	3376.84%	769.20%	3376.84%	769.20%

Table B12: Relative Bias of the modified ANC Site-Level Slope Variance Estimate (shaded cells indicate > 5% or < -5% relative bias)

Years	Trend (p)	Subpop Trend	Sampling Design	Revisit Design	Sites	PO	PWIGLS A-only	PWIGLS A	PWIGLS AI	PWIGLS B	PWIGLS BI	PWIGLS C
12	0.02	0.04	StRS	1-0	20	1.59%	1864.93%	1864.93%	1864.93%	1864.93%	1864.93%	1864.93%
12	0.02	0.04	StRS	1-0	35	-1.12%	1034.17%	1034.17%	1034.17%	1034.17%	1034.17%	1034.17%
12	0.02	0.04	StRS	1-0	50	-0.10%	716.53%	716.53%	716.53%	716.53%	716.53%	716.53%
12	0.02	0.04	StRS	1-0,1-3	20	3.37%	603.61%	596.98%	1273.11%	596.98%	1273.11%	603.61%
12	0.02	0.04	StRS	1-0,1-3	35	3.97%	185.29%	182.29%	527.24%	182.29%	527.24%	185.31%
12	0.02	0.04	StRS	1-0,1-3	50	0.23%	67.05%	65.77%	311.10%	65.77%	311.10%	67.07%
12	0.02	0.04	StRS	1-3	20	8.43%	238.78%	238.82%	1255.27%	238.82%	1255.27%	238.82%
12	0.02	0.04	StRS	1-3	35	-1.84%	71.13%	71.14%	584.56%	71.14%	584.56%	71.14%
12	0.02	0.04	StRS	1-3	50	1.55%	18.15%	18.17%	372.70%	18.17%	372.70%	18.17%
12	0.02	0.04	Unequal	1-0	20	267.08%	6735.53%	6735.53%	6735.53%	6735.53%	6735.53%	6735.53%
12	0.02	0.04	Unequal	1-0	35	253.29%	3869.48%	3869.48%	3869.48%	3869.48%	3869.48%	3869.48%
12	0.02	0.04	Unequal	1-0	50	264.38%	2704.19%	2704.19%	2704.19%	2704.19%	2704.19%	2704.19%
12	0.02	0.04	Unequal	1-0,1-3	20	257.73%	2489.36%	2478.03%	5965.85%	2478.03%	5965.85%	2489.36%
12	0.02	0.04	Unequal	1-0,1-3	35	256.67%	1083.73%	1079.63%	3470.88%	1079.63%	3470.88%	1083.73%
12	0.02	0.04	Unequal	1-0,1-3	50	251.73%	624.99%	622.11%	2298.90%	622.11%	2298.90%	624.98%
12	0.02	0.04	Unequal	1-3	20	274.80%	1514.40%	1514.41%	6357.32%	1514.41%	6357.32%	1514.41%
12	0.02	0.04	Unequal	1-3	35	267.51%	781.45%	781.38%	3425.56%	781.38%	3425.56%	781.38%
12	0.02	0.04	Unequal	1-3	50	266.83%	518.78%	518.72%	2374.24%	518.72%	2374.24%	518.72%
24	0.01	0	SRS	1-0	20	-2.00%	3063.21%	3063.21%	3063.21%	3063.21%	3063.21%	3063.21%
24	0.01	0	SRS	1-0	35	-2.27%	1746.76%	1746.76%	1746.76%	1746.76%	1746.76%	1746.76%
24	0.01	0	SRS	1-0	50	2.16%	1264.15%	1264.15%	1264.15%	1264.15%	1264.15%	1264.15%
24	0.01	0	SRS	1-0,1-3	20	-0.63%	1222.24%	1221.43%	3818.45%	1221.43%	3818.45%	1222.25%
24	0.01	0	SRS	1-0,1-3	35	-0.01%	513.52%	512.95%	2042.91%	512.95%	2042.91%	513.53%
24	0.01	0	SRS	1-0,1-3	50	-0.50%	296.50%	296.32%	1360.71%	296.32%	1360.71%	296.50%
24	0.01	0	SRS	1-3	20	1.11%	748.13%	748.13%	3292.51%	748.13%	3292.51%	748.13%
24	0.01	0	SRS	1-3	35	0.60%	385.67%	385.69%	1842.70%	385.69%	1842.70%	385.69%
24	0.01	0	SRS	1-3	50	0.19%	239.58%	239.58%	1258.34%	239.58%	1258.34%	239.58%
24	0.01	0	StRS	1-0	20	-0.69%	2850.25%	2850.25%	2850.25%	2850.25%	2850.25%	2850.25%
24	0.01	0	StRS	1-0	35	-1.49%	1643.07%	1643.07%	1643.07%	1643.07%	1643.07%	1643.07%
24	0.01	0	StRS	1-0	50	1.99%	1169.97%	1169.97%	1169.97%	1169.97%	1169.97%	1169.97%
24	0.01	0	StRS	1-0,1-3	20	0.87%	1007.22%	1006.33%	2939.04%	1006.33%	2939.04%	1007.22%
24	0.01	0	StRS	1-0,1-3	35	0.01%	392.51%	392.33%	1532.51%	392.33%	1532.51%	392.51%
24	0.01	0	StRS	1-0,1-3	50	-0.65%	211.18%	211.11%	1003.16%	211.11%	1003.16%	211.18%
24	0.01	0	StRS	1-3	20	0.53%	550.02%	550.02%	2500.12%	550.02%	2500.12%	550.02%
24	0.01	0	StRS	1-3	35	-1.11%	265.91%	265.93%	1363.73%	265.93%	1363.73%	265.93%
24	0.01	0	StRS	1-3	50	0.73%	162.86%	162.87%	951.47%	162.87%	951.47%	162.87%

Table B12: Relative Bias of the modified ANC Site-Level Slope Variance Estimate (shaded cells indicate > 5% or < -5% relative bias)

Years	Trend (p)	Subpop Trend	Sampling Design	Revisit Design	Sites	PO	PWIGLS A-only	PWIGLS A	PWIGLS AI	PWIGLS B	PWIGLS BI	PWIGLS C
24	0.01	0	Unequal	1-0	20	0.85%	2297.03%	2297.03%	2297.03%	2297.03%	2297.03%	2297.03%
24	0.01	0	Unequal	1-0	35	1.62%	1425.65%	1425.65%	1425.65%	1425.65%	1425.65%	1425.65%
24	0.01	0	Unequal	1-0	50	0.48%	950.72%	950.72%	950.72%	950.72%	950.72%	950.72%
24	0.01	0	Unequal	1-0,1-3	20	0.06%	748.14%	747.47%	2304.67%	747.47%	2304.67%	748.14%
24	0.01	0	Unequal	1-0,1-3	35	0.31%	292.12%	291.89%	1225.57%	291.89%	1225.57%	292.13%
24	0.01	0	Unequal	1-0,1-3	50	0.15%	142.16%	142.02%	767.95%	142.02%	767.95%	142.16%
24	0.01	0	Unequal	1-3	20	-1.34%	396.68%	396.69%	1886.74%	396.69%	1886.74%	396.69%
24	0.01	0	Unequal	1-3	35	-0.45%	179.61%	179.61%	1018.43%	179.61%	1018.43%	179.61%
24	0.01	0	Unequal	1-3	50	-0.86%	96.88%	96.88%	687.53%	96.88%	687.53%	96.88%
24	0.01	0.04	SRS	1-0	20	171.83%	8694.88%	8694.88%	8694.88%	8694.88%	8694.88%	8694.88%
24	0.01	0.04	SRS	1-0	35	165.58%	4924.12%	4924.12%	4924.12%	4924.12%	4924.12%	4924.12%
24	0.01	0.04	SRS	1-0	50	161.83%	3398.64%	3398.64%	3398.64%	3398.64%	3398.64%	3398.64%
24	0.01	0.04	SRS	1-0,1-3	20	162.49%	3402.96%	3402.01%	11238.21%	3402.01%	11238.21%	3402.97%
24	0.01	0.04	SRS	1-0,1-3	35	162.53%	1511.86%	1511.56%	5784.29%	1511.56%	5784.29%	1511.86%
24	0.01	0.04	SRS	1-0,1-3	50	164.92%	956.16%	956.02%	3880.54%	956.02%	3880.54%	956.16%
24	0.01	0.04	SRS	1-3	20	166.32%	2138.95%	2138.99%	8855.93%	2138.99%	8855.93%	2138.99%
24	0.01	0.04	SRS	1-3	35	163.38%	1173.02%	1173.03%	4992.11%	1173.03%	4992.11%	1173.03%
24	0.01	0.04	SRS	1-3	50	162.98%	791.94%	791.94%	3467.76%	791.94%	3467.76%	791.94%
24	0.01	0.04	StRS	1-0	20	-0.87%	2765.15%	2765.15%	2765.15%	2765.15%	2765.15%	2765.15%
24	0.01	0.04	StRS	1-0	35	0.10%	1638.98%	1638.98%	1638.98%	1638.98%	1638.98%	1638.98%
24	0.01	0.04	StRS	1-0	50	1.12%	1161.56%	1161.56%	1161.56%	1161.56%	1161.56%	1161.56%
24	0.01	0.04	StRS	1-0,1-3	20	0.84%	1007.61%	1006.67%	2883.48%	1006.67%	2883.48%	1007.62%
24	0.01	0.04	StRS	1-0,1-3	35	0.05%	385.79%	385.64%	1503.75%	385.64%	1503.75%	385.79%
24	0.01	0.04	StRS	1-0,1-3	50	0.44%	214.62%	214.58%	1018.04%	214.58%	1018.04%	214.62%
24	0.01	0.04	StRS	1-3	20	-0.71%	533.67%	533.66%	2434.68%	533.66%	2434.68%	533.66%
24	0.01	0.04	StRS	1-3	35	-1.17%	263.27%	263.27%	1353.10%	263.27%	1353.10%	263.27%
24	0.01	0.04	StRS	1-3	50	-0.60%	156.30%	156.30%	925.20%	156.30%	925.20%	156.30%
24	0.01	0.04	Unequal	1-0	20	269.39%	7365.02%	7365.02%	7365.02%	7365.02%	7365.02%	7365.02%
24	0.01	0.04	Unequal	1-0	35	268.59%	4652.79%	4652.79%	4652.79%	4652.79%	4652.79%	4652.79%
24	0.01	0.04	Unequal	1-0	50	270.34%	3183.48%	3183.48%	3183.48%	3183.48%	3183.48%	3183.48%
24	0.01	0.04	Unequal	1-0,1-3	20	270.18%	3003.12%	3001.93%	9879.68%	3001.93%	9879.68%	3003.12%
24	0.01	0.04	Unequal	1-0,1-3	35	264.14%	1315.20%	1314.81%	5038.29%	1314.81%	5038.29%	1315.21%
24	0.01	0.04	Unequal	1-0,1-3	50	267.50%	813.66%	813.38%	3331.74%	813.38%	3331.74%	813.66%
24	0.01	0.04	Unequal	1-3	20	264.43%	1841.92%	1841.92%	7667.66%	1841.92%	7667.66%	1841.92%
24	0.01	0.04	Unequal	1-3	35	266.22%	1010.82%	1010.82%	4343.29%	1010.82%	4343.29%	1010.82%
24	0.01	0.04	Unequal	1-3	50	265.34%	673.53%	673.53%	2994.11%	673.53%	2994.11%	673.53%

Table B13: Relative Bias of the ANC Year-to-Year Variance Estimate (shaded cells indicate > 5% or < -5% relative bias)

Years	Trend (p)	Subpop Trend	Sampling Design	Revisit Design	Sites	PO	PWIGLS A-only	PWIGLS A	PWIGLS AI	PWIGLS B	PWIGLS BI	PWIGLS C
12	0.02	0	SRS	1-0	20	-2.38%	2900.08%	2900.08%	2900.08%	2900.08%	2900.08%	2900.08%
12	0.02	0	SRS	1-0	35	-1.40%	1623.96%	1623.96%	1623.96%	1623.96%	1623.96%	1623.96%
12	0.02	0	SRS	1-0	50	-1.67%	1099.04%	1099.04%	1099.04%	1099.04%	1099.04%	1099.04%
12	0.02	0	SRS	1-0,1-3	20	-3.57%	1453.71%	1070.78%	1479.42%	1070.78%	1479.42%	521.49%
12	0.02	0	SRS	1-0,1-3	35	-3.05%	772.33%	428.28%	787.98%	428.28%	787.98%	177.56%
12	0.02	0	SRS	1-0,1-3	50	-2.57%	549.65%	240.64%	558.85%	240.64%	558.85%	91.07%
12	0.02	0	SRS	1-3	20	3.91%	2890.17%	647.54%	2890.18%	647.54%	2890.18%	647.54%
12	0.02	0	SRS	1-3	35	2.71%	1581.67%	320.42%	1581.67%	320.42%	1581.67%	320.42%
12	0.02	0	SRS	1-3	50	3.89%	1085.66%	196.42%	1085.66%	196.42%	1085.66%	196.42%
12	0.02	0	StRS	1-0	20	-50.04%	807.95%	807.95%	807.95%	807.95%	807.95%	807.95%
12	0.02	0	StRS	1-0	35	-49.17%	533.84%	533.84%	533.84%	533.84%	533.84%	533.84%
12	0.02	0	StRS	1-0	50	-48.72%	376.48%	376.48%	376.48%	376.48%	376.48%	376.48%
12	0.02	0	StRS	1-0,1-3	20	-51.18%	313.80%	279.17%	316.97%	279.17%	316.97%	65.52%
12	0.02	0	StRS	1-0,1-3	35	-50.61%	162.47%	98.20%	168.92%	98.20%	168.92%	-16.49%
12	0.02	0	StRS	1-0,1-3	50	-50.33%	110.75%	37.08%	122.28%	37.08%	122.28%	-38.02%
12	0.02	0	StRS	1-3	20	-52.63%	1077.81%	195.14%	1077.81%	195.14%	1077.81%	195.14%
12	0.02	0	StRS	1-3	35	-51.59%	672.53%	93.16%	672.64%	93.16%	672.64%	93.16%
12	0.02	0	StRS	1-3	50	-51.94%	467.59%	41.90%	467.59%	41.90%	467.59%	41.90%
12	0.02	0	Unequal	1-0	20	-1.59%	1351.08%	1351.08%	1351.08%	1351.08%	1351.08%	1351.08%
12	0.02	0	Unequal	1-0	35	-1.98%	759.13%	759.13%	759.13%	759.13%	759.13%	759.13%
12	0.02	0	Unequal	1-0	50	-2.41%	471.32%	471.32%	471.32%	471.32%	471.32%	471.32%
12	0.02	0	Unequal	1-0,1-3	20	1.18%	693.78%	508.43%	701.13%	508.43%	701.13%	217.51%
12	0.02	0	Unequal	1-0,1-3	35	1.12%	356.81%	178.17%	360.45%	178.17%	360.45%	45.35%
12	0.02	0	Unequal	1-0,1-3	50	0.68%	232.51%	76.28%	234.22%	76.28%	234.22%	-2.20%
12	0.02	0	Unequal	1-3	20	0.94%	1375.80%	268.95%	1375.80%	268.95%	1375.80%	268.95%
12	0.02	0	Unequal	1-3	35	-1.08%	727.27%	106.82%	727.27%	106.82%	727.27%	106.82%
12	0.02	0	Unequal	1-3	50	-0.34%	486.32%	46.58%	486.32%	46.58%	486.32%	46.58%
12	0.02	0.04	SRS	1-0	20	-5.62%	2818.04%	2818.04%	2818.04%	2818.04%	2818.04%	2818.04%
12	0.02	0.04	SRS	1-0	35	-4.83%	1570.77%	1570.77%	1570.77%	1570.77%	1570.77%	1570.77%
12	0.02	0.04	SRS	1-0	50	-5.71%	1053.66%	1053.66%	1053.66%	1053.66%	1053.66%	1053.66%
12	0.02	0.04	SRS	1-0,1-3	20	-2.26%	1446.94%	1089.98%	1474.42%	1089.98%	1474.42%	518.78%
12	0.02	0.04	SRS	1-0,1-3	35	-2.35%	762.32%	432.76%	772.06%	432.76%	772.06%	174.38%
12	0.02	0.04	SRS	1-0,1-3	50	-2.94%	528.03%	239.65%	532.47%	239.65%	532.47%	84.71%
12	0.02	0.04	SRS	1-3	20	-1.48%	2734.51%	608.63%	2734.51%	608.63%	2734.51%	608.63%
12	0.02	0.04	SRS	1-3	35	-1.92%	1507.31%	301.83%	1507.31%	301.83%	1507.31%	301.83%
12	0.02	0.04	SRS	1-3	50	-1.69%	1024.27%	181.07%	1024.27%	181.07%	1024.27%	181.07%

Table B13: Relative Bias of the ANC Year-to-Year Variance Estimate (shaded cells indicate > 5% or < -5% relative bias)

Years	Trend (p)	Subpop Trend	Sampling Design	Revisit Design	Sites	PO	PWIGLS A-only	PWIGLS A	PWIGLS AI	PWIGLS B	PWIGLS BI	PWIGLS C
12	0.02	0.04	StRS	1-0	20	-50.24%	644.06%	644.06%	644.06%	644.06%	644.06%	644.06%
12	0.02	0.04	StRS	1-0	35	-49.42%	333.41%	333.41%	333.41%	333.41%	333.41%	333.41%
12	0.02	0.04	StRS	1-0	50	-48.99%	206.35%	206.35%	206.35%	206.35%	206.35%	206.35%
12	0.02	0.04	StRS	1-0,1-3	20	-52.64%	281.25%	187.48%	282.86%	187.48%	282.86%	52.50%
12	0.02	0.04	StRS	1-0,1-3	35	-50.83%	128.47%	35.00%	129.40%	35.00%	129.40%	-27.30%
12	0.02	0.04	StRS	1-0,1-3	50	-50.57%	72.67%	-11.38%	73.35%	-11.38%	73.35%	-49.22%
12	0.02	0.04	StRS	1-3	20	-49.31%	645.68%	86.53%	646.12%	86.53%	646.12%	86.53%
12	0.02	0.04	StRS	1-3	35	-48.65%	342.99%	10.75%	343.00%	10.75%	343.00%	10.75%
12	0.02	0.04	StRS	1-3	50	-47.55%	218.70%	-20.32%	218.70%	-20.32%	218.70%	-20.32%
12	0.02	0.04	Unequal	1-0	20	3.96%	1476.12%	1476.12%	1476.12%	1476.12%	1476.12%	1476.12%
12	0.02	0.04	Unequal	1-0	35	3.51%	854.71%	854.71%	854.71%	854.71%	854.71%	854.71%
12	0.02	0.04	Unequal	1-0	50	3.32%	551.55%	551.55%	551.55%	551.55%	551.55%	551.55%
12	0.02	0.04	Unequal	1-0,1-3	20	0.82%	672.10%	535.81%	683.31%	535.81%	683.31%	208.84%
12	0.02	0.04	Unequal	1-0,1-3	35	0.58%	362.26%	210.43%	376.69%	210.43%	376.69%	47.08%
12	0.02	0.04	Unequal	1-0,1-3	50	-0.03%	239.68%	96.28%	252.87%	96.28%	252.87%	-0.09%
12	0.02	0.04	Unequal	1-3	20	5.42%	1650.76%	337.69%	1650.76%	337.69%	1650.76%	337.69%
12	0.02	0.04	Unequal	1-3	35	5.68%	912.31%	153.08%	912.31%	153.08%	912.31%	153.08%
12	0.02	0.04	Unequal	1-3	50	5.73%	629.74%	82.44%	629.74%	82.44%	629.74%	82.44%
24	0.01	0	SRS	1-0	20	-1.99%	3119.09%	3119.09%	3119.09%	3119.09%	3119.09%	3119.09%
24	0.01	0	SRS	1-0	35	-1.55%	1740.21%	1740.21%	1740.21%	1740.21%	1740.21%	1740.21%
24	0.01	0	SRS	1-0	50	-1.70%	1182.83%	1182.83%	1182.83%	1182.83%	1182.83%	1182.83%
24	0.01	0	SRS	1-0,1-3	20	1.21%	1684.95%	1219.13%	1683.16%	1219.13%	1683.16%	613.98%
24	0.01	0	SRS	1-0,1-3	35	1.22%	939.17%	495.77%	933.74%	495.77%	933.74%	230.65%
24	0.01	0	SRS	1-0,1-3	50	1.52%	677.38%	285.17%	672.07%	285.17%	672.07%	128.64%
24	0.01	0	SRS	1-3	20	-1.85%	3059.62%	689.90%	3059.61%	689.90%	3059.61%	689.90%
24	0.01	0	SRS	1-3	35	-0.03%	1731.16%	357.79%	1731.16%	357.79%	1731.16%	357.79%
24	0.01	0	SRS	1-3	50	-0.91%	1167.29%	216.82%	1167.29%	216.82%	1167.29%	216.82%
24	0.01	0	StRS	1-0	20	-52.08%	688.90%	688.90%	688.90%	688.90%	688.90%	688.90%
24	0.01	0	StRS	1-0	35	-51.16%	363.93%	363.93%	363.93%	363.93%	363.93%	363.93%
24	0.01	0	StRS	1-0	50	-50.91%	228.83%	228.83%	228.83%	228.83%	228.83%	228.83%
24	0.01	0	StRS	1-0,1-3	20	-50.96%	332.55%	227.24%	334.71%	227.24%	334.71%	73.02%
24	0.01	0	StRS	1-0,1-3	35	-50.21%	164.17%	57.44%	166.59%	57.44%	166.59%	-15.95%
24	0.01	0	StRS	1-0,1-3	50	-49.47%	107.57%	7.89%	110.99%	7.89%	110.99%	-38.95%
24	0.01	0	StRS	1-3	20	-51.33%	748.73%	112.18%	748.73%	112.18%	748.73%	112.18%
24	0.01	0	StRS	1-3	35	-49.83%	433.17%	33.29%	433.17%	33.29%	433.17%	33.29%
24	0.01	0	StRS	1-3	50	-49.81%	286.21%	-3.45%	286.21%	-3.45%	286.21%	-3.45%

Table B13: Relative Bias of the ANC Year-to-Year Variance Estimate (shaded cells indicate > 5% or < -5% relative bias)

Years	Trend (p)	Subpop Trend	Sampling Design	Revisit Design	Sites	PO	PWIGLS A-only	PWIGLS A	PWIGLS AI	PWIGLS B	PWIGLS BI	PWIGLS C
24	0.01	0	Unequal	1-0	20	2.59%	1489.12%	1489.12%	1489.12%	1489.12%	1489.12%	1489.12%
24	0.01	0	Unequal	1-0	35	2.64%	845.54%	845.54%	845.54%	845.54%	845.54%	845.54%
24	0.01	0	Unequal	1-0	50	2.99%	541.11%	541.11%	541.11%	541.11%	541.11%	541.11%
24	0.01	0	Unequal	1-0,1-3	20	-1.48%	729.97%	522.56%	731.89%	522.56%	731.89%	231.99%
24	0.01	0	Unequal	1-0,1-3	35	-1.77%	394.11%	188.14%	396.03%	188.14%	396.03%	57.22%
24	0.01	0	Unequal	1-0,1-3	50	-1.74%	262.55%	83.68%	263.96%	83.68%	263.96%	6.63%
24	0.01	0	Unequal	1-3	20	-0.40%	1521.26%	305.32%	1521.26%	305.32%	1521.26%	305.32%
24	0.01	0	Unequal	1-3	35	0.37%	826.05%	131.51%	826.05%	131.51%	826.05%	131.51%
24	0.01	0	Unequal	1-3	50	0.17%	545.37%	61.34%	545.37%	61.34%	545.37%	61.34%
24	0.01	0.04	SRS	1-0	20	-0.67%	3188.04%	3188.04%	3188.04%	3188.04%	3188.04%	3188.04%
24	0.01	0.04	SRS	1-0	35	-0.55%	1771.52%	1771.52%	1771.52%	1771.52%	1771.52%	1771.52%
24	0.01	0.04	SRS	1-0	50	-0.68%	1203.35%	1203.35%	1203.35%	1203.35%	1203.35%	1203.35%
24	0.01	0.04	SRS	1-0,1-3	20	2.89%	1709.00%	1248.08%	1710.58%	1248.08%	1710.58%	623.60%
24	0.01	0.04	SRS	1-0,1-3	35	2.72%	940.10%	506.62%	936.95%	506.62%	936.95%	230.94%
24	0.01	0.04	SRS	1-0,1-3	50	2.30%	670.71%	289.07%	666.38%	289.07%	666.38%	126.68%
24	0.01	0.04	SRS	1-3	20	3.14%	3231.58%	732.90%	3231.58%	732.90%	3231.58%	732.90%
24	0.01	0.04	SRS	1-3	35	2.57%	1783.34%	370.83%	1783.34%	370.83%	1783.34%	370.83%
24	0.01	0.04	SRS	1-3	50	2.00%	1206.16%	226.54%	1206.16%	226.54%	1206.16%	226.54%
24	0.01	0.04	StRS	1-0	20	-50.97%	710.85%	710.85%	710.85%	710.85%	710.85%	710.85%
24	0.01	0.04	StRS	1-0	35	-49.72%	376.43%	376.43%	376.43%	376.43%	376.43%	376.43%
24	0.01	0.04	StRS	1-0	50	-49.24%	236.09%	236.09%	236.09%	236.09%	236.09%	236.09%
24	0.01	0.04	StRS	1-0,1-3	20	-50.41%	330.52%	221.29%	332.52%	221.29%	332.52%	72.21%
24	0.01	0.04	StRS	1-0,1-3	35	-49.15%	163.63%	51.27%	163.68%	51.27%	163.68%	-16.12%
24	0.01	0.04	StRS	1-0,1-3	50	-49.07%	100.20%	-0.51%	99.79%	-0.51%	99.79%	-41.12%
24	0.01	0.04	StRS	1-3	20	-50.04%	715.43%	103.86%	715.43%	103.86%	715.43%	103.86%
24	0.01	0.04	StRS	1-3	35	-49.11%	385.30%	21.33%	385.30%	21.33%	385.30%	21.33%
24	0.01	0.04	StRS	1-3	50	-48.48%	245.57%	-13.61%	245.57%	-13.61%	245.57%	-13.61%
24	0.01	0.04	Unequal	1-0	20	-1.75%	1425.57%	1425.57%	1425.57%	1425.57%	1425.57%	1425.57%
24	0.01	0.04	Unequal	1-0	35	-1.31%	826.32%	826.32%	826.32%	826.32%	826.32%	826.32%
24	0.01	0.04	Unequal	1-0	50	-1.50%	528.24%	528.24%	528.24%	528.24%	528.24%	528.24%
24	0.01	0.04	Unequal	1-0,1-3	20	0.98%	759.60%	551.06%	761.85%	551.06%	761.85%	243.84%
24	0.01	0.04	Unequal	1-0,1-3	35	1.08%	429.01%	218.82%	432.20%	218.82%	432.20%	68.32%
24	0.01	0.04	Unequal	1-0,1-3	50	1.23%	296.81%	110.10%	303.31%	110.10%	303.31%	16.71%
24	0.01	0.04	Unequal	1-3	20	0.08%	1639.75%	334.94%	1639.75%	334.94%	1639.75%	334.94%
24	0.01	0.04	Unequal	1-3	35	0.20%	946.94%	161.73%	946.94%	161.73%	946.94%	161.73%
24	0.01	0.04	Unequal	1-3	50	0.69%	673.48%	93.37%	673.48%	93.37%	673.48%	93.37%

Table B14: Relative Bias of the modified ANC Year-to-Year Variance Estimate (shaded cells indicate > 5% or < -5% relative bias)

Years	Trend (p)	Subpop Trend	Sampling Design	Revisit Design	Sites	PO	PWIGLS A-only	PWIGLS A	PWIGLS AI	PWIGLS B	PWIGLS BI	PWIGLS C
12	0.02	0	SRS	1-0	20	73.07%	4857.25%	4857.25%	4857.25%	4857.25%	4857.25%	4857.25%
12	0.02	0	SRS	1-0	35	30.35%	2082.21%	2082.21%	2082.21%	2082.21%	2082.21%	2082.21%
12	0.02	0	SRS	1-0	50	12.63%	1230.14%	1230.14%	1230.14%	1230.14%	1230.14%	1230.14%
12	0.02	0	SRS	1-0,1-3	20	62.53%	2424.00%	1603.05%	2256.36%	1603.05%	2256.36%	909.86%
12	0.02	0	SRS	1-0,1-3	35	29.08%	1150.97%	506.93%	1078.69%	506.93%	1078.69%	297.96%
12	0.02	0	SRS	1-0,1-3	50	22.49%	751.04%	279.73%	715.97%	279.73%	715.97%	152.48%
12	0.02	0	SRS	1-3	20	87.89%	4530.09%	1058.05%	4532.19%	1058.05%	4532.19%	1058.05%
12	0.02	0	SRS	1-3	35	51.54%	2064.78%	439.29%	2068.08%	439.29%	2068.08%	439.29%
12	0.02	0	SRS	1-3	50	21.29%	1118.80%	204.02%	1121.09%	204.02%	1121.09%	204.02%
12	0.02	0	StRS	1-0	20	37.03%	2016.02%	2016.02%	2016.02%	2016.02%	2016.02%	2016.02%
12	0.02	0	StRS	1-0	35	-10.69%	702.56%	702.56%	702.56%	702.56%	702.56%	702.56%
12	0.02	0	StRS	1-0	50	-25.10%	383.07%	383.07%	383.07%	383.07%	383.07%	383.07%
12	0.02	0	StRS	1-0,1-3	20	35.57%	1166.95%	742.36%	1133.75%	742.36%	1133.75%	407.01%
12	0.02	0	StRS	1-0,1-3	35	-1.40%	356.67%	136.73%	345.97%	136.73%	345.97%	45.77%
12	0.02	0	StRS	1-0,1-3	50	-17.88%	187.12%	22.30%	184.34%	22.30%	184.34%	-15.30%
12	0.02	0	StRS	1-3	20	47.75%	1925.18%	407.19%	1929.36%	407.19%	1929.36%	407.19%
12	0.02	0	StRS	1-3	35	15.32%	742.55%	111.11%	743.96%	111.11%	743.96%	111.11%
12	0.02	0	StRS	1-3	50	-14.92%	373.19%	18.66%	375.03%	18.66%	375.03%	18.66%
12	0.02	0	Unequal	1-0	20	64.36%	2223.54%	2223.54%	2223.54%	2223.54%	2223.54%	2223.54%
12	0.02	0	Unequal	1-0	35	29.19%	975.42%	975.42%	975.42%	975.42%	975.42%	975.42%
12	0.02	0	Unequal	1-0	50	5.37%	480.92%	480.92%	480.92%	480.92%	480.92%	480.92%
12	0.02	0	Unequal	1-0,1-3	20	99.46%	1399.90%	930.82%	1345.52%	930.82%	1345.52%	500.20%
12	0.02	0	Unequal	1-0,1-3	35	41.23%	532.29%	212.98%	506.82%	212.98%	506.82%	101.00%
12	0.02	0	Unequal	1-0,1-3	50	22.74%	293.86%	82.54%	279.46%	82.54%	279.46%	15.85%
12	0.02	0	Unequal	1-3	20	102.22%	2383.24%	525.02%	2399.29%	525.02%	2399.29%	525.02%
12	0.02	0	Unequal	1-3	35	37.65%	858.74%	140.14%	862.71%	140.14%	862.71%	140.14%
12	0.02	0	Unequal	1-3	50	17.67%	473.11%	43.62%	474.38%	43.62%	474.38%	43.62%
12	0.02	0.04	SRS	1-0	20	48.25%	4414.72%	4414.72%	4414.72%	4414.72%	4414.72%	4414.72%
12	0.02	0.04	SRS	1-0	35	28.17%	2172.24%	2172.24%	2172.24%	2172.24%	2172.24%	2172.24%
12	0.02	0.04	SRS	1-0	50	2.84%	1183.60%	1183.60%	1183.60%	1183.60%	1183.60%	1183.60%
12	0.02	0.04	SRS	1-0,1-3	20	91.27%	2979.93%	2081.28%	2756.34%	2081.28%	2756.34%	1131.93%
12	0.02	0.04	SRS	1-0,1-3	35	37.25%	1126.84%	587.80%	1064.30%	587.80%	1064.30%	291.83%
12	0.02	0.04	SRS	1-0,1-3	50	15.25%	688.66%	265.70%	668.96%	265.70%	668.96%	133.38%
12	0.02	0.04	SRS	1-3	20	84.87%	4509.37%	1053.72%	4515.04%	1053.72%	4515.04%	1053.72%
12	0.02	0.04	SRS	1-3	35	38.08%	1915.28%	405.90%	1922.58%	405.90%	1922.58%	405.90%
12	0.02	0.04	SRS	1-3	50	25.77%	1211.90%	228.84%	1216.06%	228.84%	1216.06%	228.84%

Table B14: Relative Bias of the modified ANC Year-to-Year Variance Estimate (shaded cells indicate > 5% or < -5% relative bias)

Years	Trend (p)	Subpop Trend	Sampling Design	Revisit Design	Sites	PO	PWIGLS A-only	PWIGLS A	PWIGLS AI	PWIGLS B	PWIGLS BI	PWIGLS C
12	0.02	0.04	StRS	1-0	20	18.04%	1668.30%	1668.30%	1668.30%	1668.30%	1668.30%	1668.30%
12	0.02	0.04	StRS	1-0	35	-18.35%	605.60%	605.60%	605.60%	605.60%	605.60%	605.60%
12	0.02	0.04	StRS	1-0	50	-23.74%	335.96%	335.96%	335.96%	335.96%	335.96%	335.96%
12	0.02	0.04	StRS	1-0,1-3	20	37.55%	1128.55%	647.66%	1111.36%	647.66%	1111.36%	391.93%
12	0.02	0.04	StRS	1-0,1-3	35	12.59%	415.45%	165.13%	411.19%	165.13%	411.19%	64.53%
12	0.02	0.04	StRS	1-0,1-3	50	-17.80%	176.41%	13.93%	172.19%	13.93%	172.19%	-18.43%
12	0.02	0.04	StRS	1-3	20	47.01%	1673.27%	344.47%	1678.55%	344.47%	1678.55%	344.47%
12	0.02	0.04	StRS	1-3	35	13.72%	734.12%	108.80%	735.60%	108.80%	735.60%	108.80%
12	0.02	0.04	StRS	1-3	50	-13.33%	320.79%	5.93%	323.62%	5.93%	323.62%	5.93%
12	0.02	0.04	Unequal	1-0	20	62.60%	2470.19%	2470.19%	2470.19%	2470.19%	2470.19%	2470.19%
12	0.02	0.04	Unequal	1-0	35	11.44%	931.47%	931.47%	931.47%	931.47%	931.47%	931.47%
12	0.02	0.04	Unequal	1-0	50	5.28%	552.57%	552.57%	552.57%	552.57%	552.57%	552.57%
12	0.02	0.04	Unequal	1-0,1-3	20	89.45%	1378.76%	1026.03%	1298.94%	1026.03%	1298.94%	491.46%
12	0.02	0.04	Unequal	1-0,1-3	35	38.48%	552.75%	273.97%	521.25%	273.97%	521.25%	107.68%
12	0.02	0.04	Unequal	1-0,1-3	50	17.71%	368.82%	136.94%	354.94%	136.94%	354.94%	37.70%
12	0.02	0.04	Unequal	1-3	20	75.58%	2456.78%	539.22%	2455.80%	539.22%	2455.80%	539.22%
12	0.02	0.04	Unequal	1-3	35	27.32%	1167.53%	218.83%	1176.31%	218.83%	1176.31%	218.83%
12	0.02	0.04	Unequal	1-3	50	21.85%	743.60%	119.81%	842.02%	119.81%	842.02%	119.81%
24	0.01	0	SRS	1-0	20	36.37%	4434.82%	4434.82%	4434.82%	4434.82%	4434.82%	4434.82%
24	0.01	0	SRS	1-0	35	16.27%	2117.46%	2117.46%	2117.46%	2117.46%	2117.46%	2117.46%
24	0.01	0	SRS	1-0	50	-7.50%	1138.10%	1138.10%	1138.10%	1138.10%	1138.10%	1138.10%
24	0.01	0	SRS	1-0,1-3	20	49.00%	2742.83%	1834.24%	2698.55%	1834.24%	2698.55%	1038.96%
24	0.01	0	SRS	1-0,1-3	35	15.00%	1156.28%	574.87%	1154.01%	574.87%	1154.01%	300.06%
24	0.01	0	SRS	1-0,1-3	50	5.88%	748.70%	301.71%	750.59%	301.71%	750.59%	150.30%
24	0.01	0	SRS	1-3	20	44.78%	4441.94%	1040.58%	4456.53%	1040.58%	4456.53%	1040.58%
24	0.01	0	SRS	1-3	35	16.19%	2002.77%	425.45%	2008.98%	425.45%	2008.98%	425.45%
24	0.01	0	SRS	1-3	50	6.53%	1261.14%	240.23%	1261.13%	240.23%	1261.13%	240.23%
24	0.01	0	StRS	1-0	20	-12.91%	1463.18%	1463.18%	1463.18%	1463.18%	1463.18%	1463.18%
24	0.01	0	StRS	1-0	35	-28.96%	648.04%	648.04%	648.04%	648.04%	648.04%	648.04%
24	0.01	0	StRS	1-0	50	-36.96%	358.23%	358.23%	358.23%	358.23%	358.23%	358.23%
24	0.01	0	StRS	1-0,1-3	20	11.60%	1086.92%	701.67%	1050.86%	701.67%	1050.86%	374.83%
24	0.01	0	StRS	1-0,1-3	35	-24.77%	350.85%	130.76%	341.57%	130.76%	341.57%	43.37%
24	0.01	0	StRS	1-0,1-3	50	-36.33%	176.76%	22.95%	173.06%	22.95%	173.06%	-18.57%
24	0.01	0	StRS	1-3	20	4.02%	1680.95%	345.81%	1685.51%	345.81%	1685.51%	345.81%
24	0.01	0	StRS	1-3	35	-26.71%	546.44%	63.34%	554.20%	63.34%	554.20%	63.34%
24	0.01	0	StRS	1-3	50	-28.76%	344.90%	11.72%	346.89%	11.72%	346.89%	11.72%

Table B14: Relative Bias of the modified ANC Year-to-Year Variance Estimate (shaded cells indicate > 5% or < -5% relative bias)

Years	Trend (p)	Subpop Trend	Sampling Design	Revisit Design	Sites	PO	PWIGLS A-only	PWIGLS A	PWIGLS AI	PWIGLS B	PWIGLS BI	PWIGLS C
24	0.01	0	Unequal	1-0	20	24.40%	1808.60%	1808.60%	1808.60%	1808.60%	1808.60%	1808.60%
24	0.01	0	Unequal	1-0	35	8.54%	895.10%	895.10%	895.10%	895.10%	895.10%	895.10%
24	0.01	0	Unequal	1-0	50	8.47%	578.42%	578.42%	578.42%	578.42%	578.42%	578.42%
24	0.01	0	Unequal	1-0,1-3	20	42.14%	1123.30%	752.39%	1106.33%	752.39%	1106.33%	389.48%
24	0.01	0	Unequal	1-0,1-3	35	9.82%	463.15%	204.51%	459.86%	204.51%	459.86%	79.37%
24	0.01	0	Unequal	1-0,1-3	50	11.70%	333.26%	97.59%	333.30%	97.59%	333.30%	27.65%
24	0.01	0	Unequal	1-3	20	32.35%	1789.26%	372.89%	1793.14%	372.89%	1793.14%	372.89%
24	0.01	0	Unequal	1-3	35	25.09%	930.76%	157.95%	931.70%	157.95%	931.70%	157.95%
24	0.01	0	Unequal	1-3	50	5.67%	525.97%	56.17%	526.18%	56.17%	526.18%	56.17%
24	0.01	0.04	SRS	1-0	20	35.73%	4476.27%	4476.27%	4476.27%	4476.27%	4476.27%	4476.27%
24	0.01	0.04	SRS	1-0	35	16.89%	2156.63%	2156.63%	2156.63%	2156.63%	2156.63%	2156.63%
24	0.01	0.04	SRS	1-0	50	1.67%	1274.60%	1274.60%	1274.60%	1274.60%	1274.60%	1274.60%
24	0.01	0.04	SRS	1-0,1-3	20	49.88%	2727.62%	1893.02%	2712.58%	1893.02%	2712.58%	1031.51%
24	0.01	0.04	SRS	1-0,1-3	35	17.93%	1185.90%	609.16%	1188.59%	609.16%	1188.59%	310.23%
24	0.01	0.04	SRS	1-0,1-3	50	1.26%	776.86%	294.21%	776.41%	294.21%	776.41%	157.79%
24	0.01	0.04	SRS	1-3	20	38.56%	4382.31%	1036.61%	4442.21%	1036.61%	4442.21%	1036.61%
24	0.01	0.04	SRS	1-3	35	11.69%	1988.12%	424.43%	1997.19%	424.43%	1997.19%	424.43%
24	0.01	0.04	SRS	1-3	50	4.28%	1271.08%	242.89%	1272.05%	242.89%	1272.05%	242.89%
24	0.01	0.04	StRS	1-0	20	-5.27%	1645.43%	1645.43%	1645.43%	1645.43%	1645.43%	1645.43%
24	0.01	0.04	StRS	1-0	35	-25.08%	652.82%	652.82%	652.82%	652.82%	652.82%	652.82%
24	0.01	0.04	StRS	1-0	50	-41.07%	339.40%	339.40%	339.40%	339.40%	339.40%	339.40%
24	0.01	0.04	StRS	1-0,1-3	20	6.16%	1102.42%	653.11%	1066.15%	653.11%	1066.15%	381.72%
24	0.01	0.04	StRS	1-0,1-3	35	-29.38%	327.18%	122.17%	318.80%	122.17%	318.80%	36.23%
24	0.01	0.04	StRS	1-0,1-3	50	-38.68%	180.38%	22.78%	176.93%	22.78%	176.93%	-17.38%
24	0.01	0.04	StRS	1-3	20	0.15%	1632.62%	332.06%	1632.60%	332.06%	1632.60%	332.06%
24	0.01	0.04	StRS	1-3	35	-26.15%	634.02%	83.97%	636.50%	83.97%	636.50%	83.97%
24	0.01	0.04	StRS	1-3	50	-39.91%	289.54%	-2.22%	291.51%	-2.22%	291.51%	-2.22%
24	0.01	0.04	Unequal	1-0	20	33.35%	2051.86%	2051.86%	2051.86%	2051.86%	2051.86%	2051.86%
24	0.01	0.04	Unequal	1-0	35	11.60%	949.24%	949.24%	949.24%	949.24%	949.24%	949.24%
24	0.01	0.04	Unequal	1-0	50	5.19%	561.32%	561.32%	561.32%	561.32%	561.32%	561.32%
24	0.01	0.04	Unequal	1-0,1-3	20	37.39%	1187.52%	808.18%	1178.88%	808.18%	1178.88%	415.02%
24	0.01	0.04	Unequal	1-0,1-3	35	21.32%	574.61%	258.50%	572.14%	258.50%	572.14%	114.91%
24	0.01	0.04	Unequal	1-0,1-3	50	6.07%	330.61%	102.40%	329.82%	102.40%	329.82%	26.95%
24	0.01	0.04	Unequal	1-3	20	57.36%	2496.77%	549.06%	2490.47%	549.06%	2490.47%	549.06%
24	0.01	0.04	Unequal	1-3	35	16.06%	999.66%	175.43%	1003.04%	175.43%	1003.04%	175.43%
24	0.01	0.04	Unequal	1-3	50	11.75%	642.70%	85.91%	643.57%	85.91%	643.57%	85.91%

Table B15: Relative Bias of the ANC Residual Variance Estimate (shaded cells indicate > 5% or < -5% relative bias)

Years	Trend (p)	Subpop Trend	Sampling Design	Revisit Design	Sites	PO	PWIGLS A-only	PWIGLS A	PWIGLS AI	PWIGLS B	PWIGLS BI	PWIGLS C
12	0.02	0	SRS	1-0	20	-0.13%	-0.10%	-0.10%	-0.10%	-0.10%	-0.10%	-0.10%
12	0.02	0	SRS	1-0	35	0.31%	0.32%	0.32%	0.32%	0.32%	0.32%	0.32%
12	0.02	0	SRS	1-0	50	-0.48%	-0.48%	-0.48%	-0.48%	-0.48%	-0.48%	-0.48%
12	0.02	0	SRS	1-0,1-3	20	0.30%	10.03%	0.35%	15.07%	0.35%	15.07%	10.03%
12	0.02	0	SRS	1-0,1-3	35	-0.34%	13.90%	-0.31%	19.33%	-0.31%	19.33%	13.90%
12	0.02	0	SRS	1-0,1-3	50	0.11%	14.29%	0.13%	19.28%	0.13%	19.28%	14.29%
12	0.02	0	SRS	1-3	20	-0.74%	-0.70%	-0.70%	-0.70%	-0.70%	-0.70%	-0.70%
12	0.02	0	SRS	1-3	35	-0.40%	-0.39%	-0.39%	-0.39%	-0.39%	-0.39%	-0.39%
12	0.02	0	SRS	1-3	50	-0.25%	-0.24%	-0.24%	-0.24%	-0.24%	-0.24%	-0.24%
12	0.02	0	StRS	1-0	20	75.16%	86.09%	86.09%	86.09%	86.09%	86.09%	86.09%
12	0.02	0	StRS	1-0	35	75.14%	86.49%	86.49%	86.49%	86.49%	86.49%	86.49%
12	0.02	0	StRS	1-0	50	73.96%	85.42%	85.42%	85.42%	85.42%	85.42%	85.42%
12	0.02	0	StRS	1-0,1-3	20	77.77%	96.98%	91.05%	99.84%	91.05%	99.84%	96.98%
12	0.02	0	StRS	1-0,1-3	35	76.34%	97.99%	90.47%	100.92%	90.47%	100.92%	97.99%
12	0.02	0	StRS	1-0,1-3	50	73.99%	96.91%	88.72%	98.65%	88.72%	98.65%	96.91%
12	0.02	0	StRS	1-3	20	67.84%	81.17%	81.19%	81.17%	81.19%	81.17%	81.19%
12	0.02	0	StRS	1-3	35	68.33%	82.03%	82.01%	82.01%	82.01%	82.01%	82.01%
12	0.02	0	StRS	1-3	50	66.82%	80.11%	80.11%	80.11%	80.11%	80.11%	80.11%
12	0.02	0	Unequal	1-0	20	0.54%	13.47%	13.47%	13.47%	13.47%	13.47%	13.47%
12	0.02	0	Unequal	1-0	35	-0.19%	13.82%	13.82%	13.82%	13.82%	13.82%	13.82%
12	0.02	0	Unequal	1-0	50	0.29%	14.41%	14.41%	14.41%	14.41%	14.41%	14.41%
12	0.02	0	Unequal	1-0,1-3	20	0.70%	27.94%	17.47%	31.27%	17.47%	31.27%	27.94%
12	0.02	0	Unequal	1-0,1-3	35	0.25%	32.86%	18.77%	35.09%	18.77%	35.09%	32.86%
12	0.02	0	Unequal	1-0,1-3	50	-0.30%	33.18%	19.23%	35.15%	19.23%	35.15%	33.18%
12	0.02	0	Unequal	1-3	20	-1.64%	18.40%	18.40%	18.40%	18.40%	18.40%	18.40%
12	0.02	0	Unequal	1-3	35	0.42%	21.14%	21.13%	21.13%	21.13%	21.13%	21.13%
12	0.02	0	Unequal	1-3	50	-0.49%	21.83%	21.83%	21.83%	21.83%	21.83%	21.83%
12	0.02	0.04	SRS	1-0	20	-0.51%	-0.48%	-0.48%	-0.48%	-0.48%	-0.48%	-0.48%
12	0.02	0.04	SRS	1-0	35	0.62%	0.63%	0.63%	0.63%	0.63%	0.63%	0.63%
12	0.02	0.04	SRS	1-0	50	0.26%	0.27%	0.27%	0.27%	0.27%	0.27%	0.27%
12	0.02	0.04	SRS	1-0,1-3	20	-1.14%	8.47%	-1.10%	15.57%	-1.10%	15.57%	8.47%
12	0.02	0.04	SRS	1-0,1-3	35	-0.29%	12.82%	-0.27%	20.13%	-0.27%	20.13%	12.82%
12	0.02	0.04	SRS	1-0,1-3	50	-0.16%	14.02%	-0.14%	20.38%	-0.14%	20.38%	14.02%
12	0.02	0.04	SRS	1-3	20	-0.64%	-0.59%	-0.59%	-0.59%	-0.59%	-0.59%	-0.59%
12	0.02	0.04	SRS	1-3	35	0.54%	0.56%	0.56%	0.56%	0.56%	0.56%	0.56%
12	0.02	0.04	SRS	1-3	50	0.04%	0.05%	0.05%	0.05%	0.05%	0.05%	0.05%

Table B15: Relative Bias of the ANC Residual Variance Estimate (shaded cells indicate > 5% or < -5% relative bias)

Years	Trend (p)	Subpop Trend	Sampling Design	Revisit Design	Sites	PO	PWIGLS A-only	PWIGLS A	PWIGLS AI	PWIGLS B	PWIGLS BI	PWIGLS C
12	0.02	0.04	StRS	1-0	20	79.15%	91.49%	91.49%	91.49%	91.49%	91.49%	91.49%
12	0.02	0.04	StRS	1-0	35	79.47%	92.40%	92.40%	92.40%	92.40%	92.40%	92.40%
12	0.02	0.04	StRS	1-0	50	77.36%	90.80%	90.80%	90.80%	90.80%	90.80%	90.80%
12	0.02	0.04	StRS	1-0,1-3	20	76.10%	93.12%	88.12%	94.36%	88.12%	94.36%	93.09%
12	0.02	0.04	StRS	1-0,1-3	35	71.62%	92.91%	86.53%	94.41%	86.53%	94.41%	92.91%
12	0.02	0.04	StRS	1-0,1-3	50	68.88%	90.95%	84.29%	92.53%	84.29%	92.53%	90.95%
12	0.02	0.04	StRS	1-3	20	63.80%	80.35%	80.31%	80.31%	80.31%	80.31%	80.31%
12	0.02	0.04	StRS	1-3	35	62.07%	79.25%	79.25%	79.25%	79.25%	79.25%	79.25%
12	0.02	0.04	StRS	1-3	50	63.34%	80.40%	80.40%	80.40%	80.40%	80.40%	80.40%
12	0.02	0.04	Unequal	1-0	20	-0.68%	13.46%	13.46%	13.46%	13.46%	13.46%	13.46%
12	0.02	0.04	Unequal	1-0	35	-0.08%	14.79%	14.79%	14.79%	14.79%	14.79%	14.79%
12	0.02	0.04	Unequal	1-0	50	0.01%	14.99%	14.99%	14.99%	14.99%	14.99%	14.99%
12	0.02	0.04	Unequal	1-0,1-3	20	-0.10%	26.88%	16.84%	33.45%	16.84%	33.45%	26.88%
12	0.02	0.04	Unequal	1-0,1-3	35	-0.66%	31.77%	18.51%	37.57%	18.51%	37.57%	31.77%
12	0.02	0.04	Unequal	1-0,1-3	50	-0.65%	33.51%	19.54%	38.14%	19.54%	38.14%	33.51%
12	0.02	0.04	Unequal	1-3	20	-1.53%	22.45%	22.45%	22.45%	22.45%	22.45%	22.45%
12	0.02	0.04	Unequal	1-3	35	-0.77%	23.82%	23.82%	23.82%	23.82%	23.82%	23.82%
12	0.02	0.04	Unequal	1-3	50	-0.26%	23.57%	23.57%	23.57%	23.57%	23.57%	23.57%
24	0.01	0	SRS	1-0	20	-0.09%	-0.08%	-0.08%	-0.08%	-0.08%	-0.08%	-0.08%
24	0.01	0	SRS	1-0	35	0.02%	0.02%	0.02%	0.02%	0.02%	0.02%	0.02%
24	0.01	0	SRS	1-0	50	-0.08%	-0.08%	-0.08%	-0.08%	-0.08%	-0.08%	-0.08%
24	0.01	0	SRS	1-0,1-3	20	0.32%	14.53%	0.33%	15.69%	0.33%	15.69%	14.53%
24	0.01	0	SRS	1-0,1-3	35	-0.18%	15.81%	-0.18%	16.51%	-0.18%	16.51%	15.81%
24	0.01	0	SRS	1-0,1-3	50	-0.12%	15.21%	-0.12%	15.69%	-0.12%	15.69%	15.21%
24	0.01	0	SRS	1-3	20	-0.31%	-0.30%	-0.30%	-0.30%	-0.30%	-0.30%	-0.30%
24	0.01	0	SRS	1-3	35	-0.08%	-0.08%	-0.08%	-0.08%	-0.08%	-0.08%	-0.08%
24	0.01	0	SRS	1-3	50	0.02%	0.02%	0.02%	0.02%	0.02%	0.02%	0.02%
24	0.01	0	StRS	1-0	20	78.98%	90.93%	90.93%	90.93%	90.93%	90.93%	90.93%
24	0.01	0	StRS	1-0	35	77.08%	88.73%	88.73%	88.73%	88.73%	88.73%	88.73%
24	0.01	0	StRS	1-0	50	77.18%	88.63%	88.63%	88.63%	88.63%	88.63%	88.63%
24	0.01	0	StRS	1-0,1-3	20	79.58%	100.00%	93.50%	103.21%	93.50%	103.21%	100.00%
24	0.01	0	StRS	1-0,1-3	35	78.66%	100.92%	93.61%	103.41%	93.61%	103.41%	100.92%
24	0.01	0	StRS	1-0,1-3	50	77.55%	100.19%	93.07%	102.09%	93.07%	102.09%	100.19%
24	0.01	0	StRS	1-3	20	78.26%	95.68%	95.68%	95.68%	95.68%	95.68%	95.68%
24	0.01	0	StRS	1-3	35	75.71%	92.59%	92.59%	92.59%	92.59%	92.59%	92.59%
24	0.01	0	StRS	1-3	50	74.86%	91.83%	91.83%	91.83%	91.83%	91.83%	91.83%

Table B15: Relative Bias of the ANC Residual Variance Estimate (shaded cells indicate > 5% or < -5% relative bias)

Years	Trend (p)	Subpop Trend	Sampling Design	Revisit Design	Sites	PO	PWIGLS A-only	PWIGLS A	PWIGLS AI	PWIGLS B	PWIGLS BI	PWIGLS C
24	0.01	0	Unequal	1-0	20	-0.12%	13.32%	13.32%	13.32%	13.32%	13.32%	13.32%
24	0.01	0	Unequal	1-0	35	-0.17%	14.16%	14.16%	14.16%	14.16%	14.16%	14.16%
24	0.01	0	Unequal	1-0	50	-0.21%	14.22%	14.22%	14.22%	14.22%	14.22%	14.22%
24	0.01	0	Unequal	1-0,1-3	20	0.81%	28.94%	16.73%	30.31%	16.73%	30.31%	28.94%
24	0.01	0	Unequal	1-0,1-3	35	-0.45%	31.40%	17.16%	32.36%	17.16%	32.36%	31.40%
24	0.01	0	Unequal	1-0,1-3	50	0.30%	31.10%	17.74%	31.74%	17.74%	31.74%	31.10%
24	0.01	0	Unequal	1-3	20	-0.87%	17.98%	17.98%	17.98%	17.98%	17.98%	17.98%
24	0.01	0	Unequal	1-3	35	-0.18%	18.85%	18.85%	18.85%	18.85%	18.85%	18.85%
24	0.01	0	Unequal	1-3	50	-0.45%	18.61%	18.61%	18.61%	18.61%	18.61%	18.61%
24	0.01	0.04	SRS	1-0	20	0.05%	0.06%	0.06%	0.06%	0.06%	0.06%	0.06%
24	0.01	0.04	SRS	1-0	35	0.45%	0.45%	0.45%	0.45%	0.45%	0.45%	0.45%
24	0.01	0.04	SRS	1-0	50	0.09%	0.09%	0.09%	0.09%	0.09%	0.09%	0.09%
24	0.01	0.04	SRS	1-0,1-3	20	-0.11%	14.15%	-0.10%	14.64%	-0.10%	14.64%	14.15%
24	0.01	0.04	SRS	1-0,1-3	35	-0.03%	16.23%	-0.03%	16.52%	-0.03%	16.52%	16.23%
24	0.01	0.04	SRS	1-0,1-3	50	0.36%	15.43%	0.36%	15.62%	0.36%	15.62%	15.43%
24	0.01	0.04	SRS	1-3	20	-0.34%	-0.33%	-0.33%	-0.33%	-0.33%	-0.33%	-0.33%
24	0.01	0.04	SRS	1-3	35	0.28%	0.29%	0.29%	0.29%	0.29%	0.29%	0.29%
24	0.01	0.04	SRS	1-3	50	-0.09%	-0.09%	-0.09%	-0.09%	-0.09%	-0.09%	-0.09%
24	0.01	0.04	StRS	1-0	20	76.70%	88.32%	88.32%	88.32%	88.32%	88.32%	88.32%
24	0.01	0.04	StRS	1-0	35	76.06%	87.53%	87.53%	87.53%	87.53%	87.53%	87.53%
24	0.01	0.04	StRS	1-0	50	74.58%	86.07%	86.07%	86.07%	86.07%	86.07%	86.07%
24	0.01	0.04	StRS	1-0,1-3	20	80.09%	101.54%	95.20%	104.54%	95.20%	104.54%	101.54%
24	0.01	0.04	StRS	1-0,1-3	35	77.66%	101.58%	94.07%	104.05%	94.07%	104.05%	101.58%
24	0.01	0.04	StRS	1-0,1-3	50	77.92%	101.83%	94.36%	103.80%	94.36%	103.80%	101.83%
24	0.01	0.04	StRS	1-3	20	78.52%	98.11%	98.11%	98.11%	98.11%	98.11%	98.11%
24	0.01	0.04	StRS	1-3	35	75.63%	94.59%	94.59%	94.59%	94.59%	94.59%	94.59%
24	0.01	0.04	StRS	1-3	50	74.92%	93.75%	93.75%	93.75%	93.75%	93.75%	93.75%
24	0.01	0.04	Unequal	1-0	20	0.30%	13.37%	13.37%	13.37%	13.37%	13.37%	13.37%
24	0.01	0.04	Unequal	1-0	35	-0.11%	13.40%	13.40%	13.40%	13.40%	13.40%	13.40%
24	0.01	0.04	Unequal	1-0	50	0.09%	13.84%	13.84%	13.84%	13.84%	13.84%	13.84%
24	0.01	0.04	Unequal	1-0,1-3	20	-0.09%	27.39%	14.97%	28.04%	14.97%	28.04%	27.39%
24	0.01	0.04	Unequal	1-0,1-3	35	-0.09%	30.38%	16.02%	30.82%	16.02%	30.82%	30.38%
24	0.01	0.04	Unequal	1-0,1-3	50	-0.06%	29.82%	16.38%	30.15%	16.38%	30.15%	29.82%
24	0.01	0.04	Unequal	1-3	20	-0.14%	16.75%	16.75%	16.75%	16.75%	16.75%	16.75%
24	0.01	0.04	Unequal	1-3	35	-0.46%	16.29%	16.29%	16.29%	16.29%	16.29%	16.29%
24	0.01	0.04	Unequal	1-3	50	0.14%	16.87%	16.87%	16.87%	16.87%	16.87%	16.87%

Table B16: Relative Bias of the modified ANC Residual Variance Estimate (shaded cells indicate > 5% or < -5% relative bias)

Years	Trend (p)	Subpop Trend	Sampling Design	Revisit Design	Sites	PO	PWIGLS A-only	PWIGLS A	PWIGLS AI	PWIGLS B	PWIGLS BI	PWIGLS C
12	0.02	0	SRS	1-0	20	-1.35%	-1.22%	-1.22%	-1.22%	-1.22%	-1.22%	-1.22%
12	0.02	0	SRS	1-0	35	-0.22%	-0.15%	-0.15%	-0.15%	-0.15%	-0.15%	-0.15%
12	0.02	0	SRS	1-0	50	-0.48%	-0.42%	-0.42%	-0.42%	-0.42%	-0.42%	-0.42%
12	0.02	0	SRS	1-0,1-3	20	-0.95%	-0.86%	-0.73%	2.93%	-0.73%	2.93%	-0.86%
12	0.02	0	SRS	1-0,1-3	35	0.02%	-0.03%	0.18%	4.81%	0.18%	4.81%	-0.03%
12	0.02	0	SRS	1-0,1-3	50	0.39%	0.40%	0.51%	5.44%	0.51%	5.44%	0.39%
12	0.02	0	SRS	1-3	20	-1.94%	-1.81%	-1.81%	-1.81%	-1.81%	-1.81%	-1.81%
12	0.02	0	SRS	1-3	35	-0.48%	-0.36%	-0.35%	-0.36%	-0.35%	-0.36%	-0.35%
12	0.02	0	SRS	1-3	50	0.24%	0.36%	0.36%	0.36%	0.36%	0.36%	0.36%
12	0.02	0	StRS	1-0	20	-0.78%	0.49%	0.49%	0.49%	0.49%	0.49%	0.49%
12	0.02	0	StRS	1-0	35	0.24%	1.63%	1.63%	1.63%	1.63%	1.63%	1.63%
12	0.02	0	StRS	1-0	50	0.21%	1.61%	1.61%	1.61%	1.61%	1.61%	1.61%
12	0.02	0	StRS	1-0,1-3	20	0.14%	2.51%	2.64%	4.42%	2.64%	4.42%	2.51%
12	0.02	0	StRS	1-0,1-3	35	0.71%	4.60%	4.69%	6.85%	4.69%	6.85%	4.60%
12	0.02	0	StRS	1-0,1-3	50	0.51%	5.19%	5.28%	7.26%	5.28%	7.26%	5.19%
12	0.02	0	StRS	1-3	20	-0.76%	5.30%	5.29%	5.29%	5.29%	5.29%	5.29%
12	0.02	0	StRS	1-3	35	-0.79%	6.15%	6.14%	6.14%	6.14%	6.14%	6.14%
12	0.02	0	StRS	1-3	50	0.03%	6.96%	6.96%	6.96%	6.96%	6.96%	6.96%
12	0.02	0	Unequal	1-0	20	-1.28%	-0.34%	-0.34%	-0.34%	-0.34%	-0.34%	-0.34%
12	0.02	0	Unequal	1-0	35	-0.12%	1.11%	1.11%	1.11%	1.11%	1.11%	1.11%
12	0.02	0	Unequal	1-0	50	-0.26%	0.96%	0.96%	0.96%	0.96%	0.96%	0.96%
12	0.02	0	Unequal	1-0,1-3	20	-0.60%	1.24%	1.36%	3.84%	1.36%	3.84%	1.24%
12	0.02	0	Unequal	1-0,1-3	35	-0.16%	3.26%	3.41%	6.43%	3.41%	6.43%	3.26%
12	0.02	0	Unequal	1-0,1-3	50	-0.75%	3.25%	3.29%	6.71%	3.29%	6.71%	3.25%
12	0.02	0	Unequal	1-3	20	-0.15%	4.66%	4.65%	4.65%	4.65%	4.65%	4.65%
12	0.02	0	Unequal	1-3	35	-0.74%	5.31%	5.31%	5.30%	5.31%	5.30%	5.31%
12	0.02	0	Unequal	1-3	50	-1.19%	4.62%	4.62%	4.62%	4.62%	4.62%	4.62%
12	0.02	0.04	SRS	1-0	20	-0.44%	-0.36%	-0.36%	-0.36%	-0.36%	-0.36%	-0.36%
12	0.02	0.04	SRS	1-0	35	-0.30%	-0.26%	-0.26%	-0.26%	-0.26%	-0.26%	-0.26%
12	0.02	0.04	SRS	1-0	50	0.35%	0.39%	0.39%	0.39%	0.39%	0.39%	0.39%
12	0.02	0.04	SRS	1-0,1-3	20	-0.46%	-0.39%	-0.28%	5.43%	-0.28%	5.43%	-0.39%
12	0.02	0.04	SRS	1-0,1-3	35	-1.00%	-0.95%	-0.88%	4.53%	-0.88%	4.53%	-0.95%
12	0.02	0.04	SRS	1-0,1-3	50	-0.15%	-0.14%	-0.05%	4.01%	-0.05%	4.01%	-0.15%
12	0.02	0.04	SRS	1-3	20	-0.51%	-0.32%	-0.32%	-0.32%	-0.32%	-0.32%	-0.32%
12	0.02	0.04	SRS	1-3	35	-0.92%	-0.79%	-0.80%	-0.80%	-0.80%	-0.80%	-0.80%
12	0.02	0.04	SRS	1-3	50	0.20%	0.30%	0.30%	0.30%	0.30%	0.30%	0.30%

Table B16: Relative Bias of the modified ANC Residual Variance Estimate (shaded cells indicate > 5% or < -5% relative bias)

Years	Trend (p)	Subpop Trend	Sampling Design	Revisit Design	Sites	PO	PWIGLS A-only	PWIGLS A	PWIGLS AI	PWIGLS B	PWIGLS BI	PWIGLS C
12	0.02	0.04	StRS	1-0	20	-0.34%	1.15%	1.15%	1.15%	1.15%	1.15%	1.15%
12	0.02	0.04	StRS	1-0	35	0.71%	2.26%	2.26%	2.26%	2.26%	2.26%	2.26%
12	0.02	0.04	StRS	1-0	50	0.23%	1.88%	1.88%	1.88%	1.88%	1.88%	1.88%
12	0.02	0.04	StRS	1-0,1-3	20	-1.15%	1.46%	1.67%	2.90%	1.67%	2.90%	1.45%
12	0.02	0.04	StRS	1-0,1-3	35	-0.60%	3.63%	3.74%	5.85%	3.74%	5.85%	3.63%
12	0.02	0.04	StRS	1-0,1-3	50	-0.11%	4.86%	4.96%	6.78%	4.96%	6.78%	4.85%
12	0.02	0.04	StRS	1-3	20	-1.68%	4.81%	4.81%	4.81%	4.81%	4.81%	4.81%
12	0.02	0.04	StRS	1-3	35	-0.32%	6.06%	6.06%	6.06%	6.06%	6.06%	6.06%
12	0.02	0.04	StRS	1-3	50	0.09%	7.25%	7.25%	7.25%	7.25%	7.25%	7.25%
12	0.02	0.04	Unequal	1-0	20	-0.45%	-0.07%	-0.07%	-0.07%	-0.07%	-0.07%	-0.07%
12	0.02	0.04	Unequal	1-0	35	0.36%	0.94%	0.94%	0.94%	0.94%	0.94%	0.94%
12	0.02	0.04	Unequal	1-0	50	0.09%	0.84%	0.84%	0.84%	0.84%	0.84%	0.84%
12	0.02	0.04	Unequal	1-0,1-3	20	-0.53%	1.71%	1.72%	6.12%	1.72%	6.12%	1.71%
12	0.02	0.04	Unequal	1-0,1-3	35	-0.28%	3.04%	3.11%	6.42%	3.11%	6.42%	3.04%
12	0.02	0.04	Unequal	1-0,1-3	50	0.35%	4.31%	4.39%	6.84%	4.39%	6.84%	4.31%
12	0.02	0.04	Unequal	1-3	20	-1.41%	4.12%	4.12%	4.12%	4.12%	4.12%	4.12%
12	0.02	0.04	Unequal	1-3	35	-0.86%	5.69%	5.69%	5.69%	5.69%	5.69%	5.69%
12	0.02	0.04	Unequal	1-3	50	-0.17%	6.18%	6.17%	6.17%	6.17%	6.17%	6.17%
24	0.01	0	SRS	1-0	20	-0.44%	-0.42%	-0.42%	-0.42%	-0.42%	-0.42%	-0.42%
24	0.01	0	SRS	1-0	35	0.28%	0.30%	0.30%	0.30%	0.30%	0.30%	0.30%
24	0.01	0	SRS	1-0	50	-0.09%	-0.08%	-0.08%	-0.08%	-0.08%	-0.08%	-0.08%
24	0.01	0	SRS	1-0,1-3	20	-0.54%	-0.49%	-0.50%	0.43%	-0.50%	0.43%	-0.49%
24	0.01	0	SRS	1-0,1-3	35	-0.21%	-0.13%	-0.18%	0.37%	-0.18%	0.37%	-0.13%
24	0.01	0	SRS	1-0,1-3	50	0.05%	0.15%	0.07%	0.49%	0.07%	0.49%	0.15%
24	0.01	0	SRS	1-3	20	0.01%	0.07%	0.06%	0.07%	0.06%	0.07%	0.06%
24	0.01	0	SRS	1-3	35	-0.09%	-0.05%	-0.05%	-0.05%	-0.05%	-0.05%	-0.05%
24	0.01	0	SRS	1-3	50	-0.23%	-0.20%	-0.20%	-0.20%	-0.20%	-0.20%	-0.20%
24	0.01	0	StRS	1-0	20	0.30%	0.53%	0.53%	0.53%	0.53%	0.53%	0.53%
24	0.01	0	StRS	1-0	35	0.27%	0.46%	0.46%	0.46%	0.46%	0.46%	0.46%
24	0.01	0	StRS	1-0	50	0.21%	0.41%	0.41%	0.41%	0.41%	0.41%	0.41%
24	0.01	0	StRS	1-0,1-3	20	-0.18%	1.03%	1.04%	2.41%	1.04%	2.41%	1.03%
24	0.01	0	StRS	1-0,1-3	35	0.16%	2.03%	2.03%	3.00%	2.03%	3.00%	2.03%
24	0.01	0	StRS	1-0,1-3	50	0.45%	2.58%	2.57%	3.28%	2.57%	3.28%	2.58%
24	0.01	0	StRS	1-3	20	-0.66%	2.27%	2.27%	2.27%	2.27%	2.27%	2.27%
24	0.01	0	StRS	1-3	35	0.74%	3.80%	3.80%	3.80%	3.80%	3.80%	3.80%
24	0.01	0	StRS	1-3	50	0.23%	3.22%	3.22%	3.22%	3.22%	3.22%	3.22%

Table B16: Relative Bias of the modified ANC Residual Variance Estimate (shaded cells indicate > 5% or < -5% relative bias)

Years	Trend (p)	Subpop Trend	Sampling Design	Revisit Design	Sites	PO	PWIGLS A-only	PWIGLS A	PWIGLS AI	PWIGLS B	PWIGLS BI	PWIGLS C
24	0.01	0	Unequal	1-0	20	-0.64%	-0.31%	-0.31%	-0.31%	-0.31%	-0.31%	-0.31%
24	0.01	0	Unequal	1-0	35	-0.35%	0.07%	0.07%	0.07%	0.07%	0.07%	0.07%
24	0.01	0	Unequal	1-0	50	-0.14%	0.41%	0.41%	0.41%	0.41%	0.41%	0.41%
24	0.01	0	Unequal	1-0,1-3	20	-0.40%	1.45%	1.41%	2.36%	1.41%	2.36%	1.45%
24	0.01	0	Unequal	1-0,1-3	35	-0.15%	2.59%	2.53%	3.20%	2.53%	3.20%	2.59%
24	0.01	0	Unequal	1-0,1-3	50	-0.20%	3.09%	3.04%	3.47%	3.04%	3.47%	3.09%
24	0.01	0	Unequal	1-3	20	-0.12%	4.07%	4.06%	4.06%	4.06%	4.06%	4.06%
24	0.01	0	Unequal	1-3	35	-0.04%	4.14%	4.13%	4.13%	4.13%	4.13%	4.13%
24	0.01	0	Unequal	1-3	50	-0.29%	4.05%	4.05%	4.05%	4.05%	4.05%	4.05%
24	0.01	0.04	SRS	1-0	20	-0.57%	-0.57%	-0.57%	-0.57%	-0.57%	-0.57%	-0.57%
24	0.01	0.04	SRS	1-0	35	-0.27%	-0.26%	-0.26%	-0.26%	-0.26%	-0.26%	-0.26%
24	0.01	0.04	SRS	1-0	50	-0.25%	-0.24%	-0.24%	-0.24%	-0.24%	-0.24%	-0.24%
24	0.01	0.04	SRS	1-0,1-3	20	-0.15%	-0.08%	-0.13%	0.29%	-0.13%	0.29%	-0.08%
24	0.01	0.04	SRS	1-0,1-3	35	-0.16%	-0.06%	-0.14%	0.14%	-0.14%	0.14%	-0.06%
24	0.01	0.04	SRS	1-0,1-3	50	-0.58%	-0.54%	-0.56%	-0.40%	-0.56%	-0.40%	-0.54%
24	0.01	0.04	SRS	1-3	20	-0.28%	-0.24%	-0.26%	-0.26%	-0.26%	-0.26%	-0.26%
24	0.01	0.04	SRS	1-3	35	-0.11%	-0.09%	-0.09%	-0.09%	-0.09%	-0.09%	-0.09%
24	0.01	0.04	SRS	1-3	50	0.08%	0.10%	0.10%	0.10%	0.10%	0.10%	0.10%
24	0.01	0.04	StRS	1-0	20	0.34%	0.57%	0.57%	0.57%	0.57%	0.57%	0.57%
24	0.01	0.04	StRS	1-0	35	0.17%	0.40%	0.40%	0.40%	0.40%	0.40%	0.40%
24	0.01	0.04	StRS	1-0	50	0.68%	0.86%	0.86%	0.86%	0.86%	0.86%	0.86%
24	0.01	0.04	StRS	1-0,1-3	20	-0.05%	1.13%	1.21%	2.53%	1.21%	2.53%	1.12%
24	0.01	0.04	StRS	1-0,1-3	35	0.18%	2.02%	2.01%	2.99%	2.01%	2.99%	2.02%
24	0.01	0.04	StRS	1-0,1-3	50	0.28%	2.40%	2.40%	3.09%	2.40%	3.09%	2.40%
24	0.01	0.04	StRS	1-3	20	-0.36%	2.67%	2.67%	2.67%	2.67%	2.67%	2.67%
24	0.01	0.04	StRS	1-3	35	0.27%	3.29%	3.29%	3.29%	3.29%	3.29%	3.29%
24	0.01	0.04	StRS	1-3	50	0.60%	3.64%	3.63%	3.63%	3.63%	3.63%	3.63%
24	0.01	0.04	Unequal	1-0	20	-0.75%	-0.62%	-0.62%	-0.62%	-0.62%	-0.62%	-0.62%
24	0.01	0.04	Unequal	1-0	35	0.35%	0.52%	0.52%	0.52%	0.52%	0.52%	0.52%
24	0.01	0.04	Unequal	1-0	50	0.18%	0.38%	0.38%	0.38%	0.38%	0.38%	0.38%
24	0.01	0.04	Unequal	1-0,1-3	20	-0.28%	0.55%	0.49%	0.94%	0.49%	0.94%	0.55%
24	0.01	0.04	Unequal	1-0,1-3	35	-0.24%	1.03%	0.99%	1.30%	0.99%	1.30%	1.03%
24	0.01	0.04	Unequal	1-0,1-3	50	-0.18%	1.50%	1.44%	1.71%	1.44%	1.71%	1.50%
24	0.01	0.04	Unequal	1-3	20	0.08%	1.96%	1.96%	1.96%	1.96%	1.96%	1.96%
24	0.01	0.04	Unequal	1-3	35	0.20%	2.32%	2.32%	2.32%	2.32%	2.32%	2.32%
24	0.01	0.04	Unequal	1-3	50	-0.28%	1.83%	1.83%	1.83%	1.83%	1.83%	1.83%

Table B16: Relative Bias of the modified ANC Residual Variance Estimate (shaded cells indicate > 5% or < -5% relative bias)
